# The phytosanitary risks posed by seeds for sowing trade networks

**DOI:** 10.1371/journal.pone.0259912

**Published:** 2021-11-30

**Authors:** Christopher E. Buddenhagen, Jesse M. Rubenstein, John G. Hampton, M. Philip Rolston

**Affiliations:** 1 AgResearch Ltd., Hamilton, New Zealand; 2 Bio-Protection Research Centre, Lincoln University, Lincoln, New Zealand; 3 Seed Research Centre, Lincoln University, Lincoln, New Zealand; 4 Foundation for Arable Research, Christchurch, New Zealand; Canakkale Onsekiz Mart University, TURKEY

## Abstract

When successful, the operation of local and international networks of crop seed distribution or “seed systems” ensures farmer access to seed and impacts rural livelihoods and food security. Farmers are both consumers and producers in seed systems and benefit from access to global markets. However, phytosanitary measures and seed purity tests are also needed to maintain seed quality and prevent the spread of costly weeds, pests and diseases, in some countries regulatory controls have been in place since the 1800s. Nevertheless, seed contaminants are internationally implicated in between 7% and 37% of the invasive plant species and many of the agricultural pests and diseases. We assess biosecurity risk across international seed trade networks of forage crops using models of contaminant spread that integrate network connectivity and trade volume. To stochastically model hypothetical contaminants through global seed trade networks, realistic dispersal probabilities were estimated from quarantine weed seed detections and incursions from border security interception data in New Zealand. For our test case we use contaminants linked to the global trade of ryegrass and clover seed. Between 2014 and 2018 only four quarantine weed species (222 species and several genera are on the quarantine schedule) warranting risk mitigation were detected at the border. Quarantine weeds were rare considering that average import volumes were over 190 tonnes for ryegrass and clover, but 105 unregulated contaminant species were allowed in. Ryegrass and clover seed imports each led to one post-border weed incursion response over 20 years. Trade reports revealed complex global seed trade networks spanning >134 (ryegrass) and >110 (clover) countries. Simulations showed contaminants could disperse to as many as 50 (clover) or 80 (ryegrass) countries within 10 time-steps. Risk assessed via network models differed 18% (ryegrass) or 48% (clover) of the time compared to risk assessed on trade volumes. We conclude that biosecurity risk is driven by network position, the number of trading connections and trade volume. Risk mitigation measures could involve the use of more comprehensive lists of regulated species, comprehensive inspection protocols, or the addition of field surveillance at farms where seed is planted.

## 1. Introduction

Seeds are arguably a farm’s most important input (and often also a major output). Networks of crop seed distribution operate regionally, and internationally, driving the success and profitability of agricultural systems [1]. Such networks, or “seed systems”, encompass biophysical and societal elements that provide benefits to stakeholders while mitigating risks. For example: plant breeders have put in place processes and rules to safeguard a return on any investments made, i.e. plant variety rights as intellectual property [2]; and best practices (e.g. seed cleaning), and regulatory frameworks are put in place regionally and internationally to certify seed is of good quality (is viable) [3,4] and not contaminated with pests, weeds, or diseases [5]. Here we are interested in seed for sowing (as opposed to seed for human or animal consumption) that is moved across international borders. This can include germplasm for varietal development and research [6], seed for multiplication, which may or may not be certified or named varieties. The actors in these seed systems may involve individual gardeners, farmers, merchants, agronomists, small businesses, or large seed companies that increasingly operate internationally, as well as governmental regulatory and international bodies like the International Plant Protection Convention (IPPC) and research institutions. Seed producers often take advantage alternating seasons to produce fresh seed in Northern and Southern hemispheres thus creating surprisingly distant connections. Seed systems represent a key component in global food security, and support farmers and rural areas economically [1]. New Zealand benefits greatly from its participation in the international seed system which directly contributes between 300 to 600 million (NZD) annually to the gross domestic product (GDP) and approximately ten times that indirectly [7,8]. Globally, it is a leading seed producer of crops like ryegrass, radish, white clover, and carrots [7].

Imported commodities such as seed for sowing can result in the introduction and unintended establishment of invasive species, *via* direct release, or escape from cultivation [9,10]. Accidental introductions of commodity contaminants, including seed for sowing are believed to account for the establishment of an important proportion of invasive plant species in various jurisdictions e.g., 14% British Isles, 15% Galapagos, 7% United States, 25% New Zealand, ca. 20% Australia, and 13 to 37% in Europe [11–17]. Until 1950, most naturalized plants in New Zealand entered as crop seed contaminants, but since then deliberately introduced ornamental escapes have become increasingly important [12,18,19]. Even at low levels of contamination, thousands of weed seeds may be planted along with a crops and pastures, and facilitate weed establishment [3,20,21]. New Zealand began regulating weed and invasive species introductions early after European colonization, starting with regional laws including the Control of Thistles Acts of 1854 (Wellington) and 1857 (Auckland), followed by national laws in The Noxious Weeds Act of 1900, and culminating with the Biosecurity Act of 1993 (since amended) [22]. New Zealand, like other countries, regulates a limited number of species and follows international standards under the World Trade Organization’s Agreement on the Application of Sanitary and Phytosanitary Measures [23]. In New Zealand regulations involve official “black” and “white” species lists inferring relative risk is high or low, but such systems have their limits [12,24]. As we will show, seed companies, farmers (and farms) and the ecological systems these operate in are interacting with analogous regulatory agencies but still share propagules from a long list of unregulated species throughout a global trade network. Border inspection is just one of the steps designed to lower the likelihood of a quarantine pest entering a country. Governmental plant protection officials aim for appropriate level of protection where biosecurity risks are brought to a low level but not zero. Even with biosecurity measures the total number of introduced plant species in 2020 within New Zealand is likely close to 30,000 [12,25], and naturalization rates are still high [22]. At least 1798 introduced species are known to be well established in the wild with a further 1043 species beginning to establish [19].

By way of demonstration, we focus on biosecurity risks from regulated non-crop seed contaminants (e.g., weed seeds) associated with imported ryegrass (*Lolium* spp., *L*. *perenne*, *L*. *multiflorum*) and clover (*Trifolium* spp., *T*. *repens* L., *T*. *pratense* L., and rarely *T*. *alexandrinum* L.). These species are internationally important high-quality forages and are key elements of most pasture systems in New Zealand, where pastures occupy ca. 37% of the land area [26]. Ryegrass and clover are also common in pastures of Europe, southeastern Australia, northwestern, and southeastern United States, Argentina, Chile, and Uruguay. Free trade in ryegrass and clover seed provides important benefits, but the biosecurity risks need to be managed on the seed for sowing pathway [27]. Biosecurity efforts typically involve the official inspection of imported seed lots at the border to detect regulated species, which if found may result in shipments being destroyed, reshipped, or recleaned. Seed sampling methods [5] focus on taking a representative and random sample of the seed lot for inspection. Reciprocally, most countries would not issue a phytosanitary certificate for export if their export assurance systems identified a quarantine weed in a seed lot destined for a market where it is regulated. For seed certification within New Zealand usually 0.5% to 1% weed seed is allowed [28,29] but for regulated quarantine weeds New Zealand border inspection protocols require 95% confidence that the maximum pest limit of 0.01% of quarantine weed seed contamination is not exceeded in any imported seed lot, notably all seed lots are inspected at five times the International Seed Testing Association’s recommended standard [5,30–32]. In this article, considering New Zealand is itself a major trader of ryegrass and clover, and trades with the other major traders, contaminants detected there provide insights into the movement of weed seed contaminants of most seed producing countries [27].

We set out to stochastically model the spread contaminants through ryegrass and clover trade networks. Treating invasive species, alleles, disease and pest spread as an epidemiological problem is an emerging area of research [33] Network models are an insightful means to understand biosecurity risk in complex plant trade networks, locally or regionally [6,10,34–41].We briefly examine the types of weeds that are transported in shipments of ryegrass and clover seed. We then use information about rare border detections of quarantine weeds documented by Rubenstein et al. [27], and weed incursions responses of regulated species in New Zealand as a basis for setting the probability of dispersal between nodes (countries) in order to model the spread of hypothetical contaminants across the international trade network. This type of analysis can be used to alert farmers, government agencies or seed industry stakeholders about high-risk trading partners, and pathways.

## 2. Materials and methods

We bring two datasets together for our modelling exercise, one is a publicly available dataset about international trade in a number of different crops from the United Nations Comtrade Database [42] and the other delves into the relevant border detection data for seed contaminants in New Zealand reported on by Rubenstein et al [27] to estimate realistic rates of spread for quarantine weeds through the network.

### 2.1 International trade networks for ryegrass and clover

**Fig 1:**
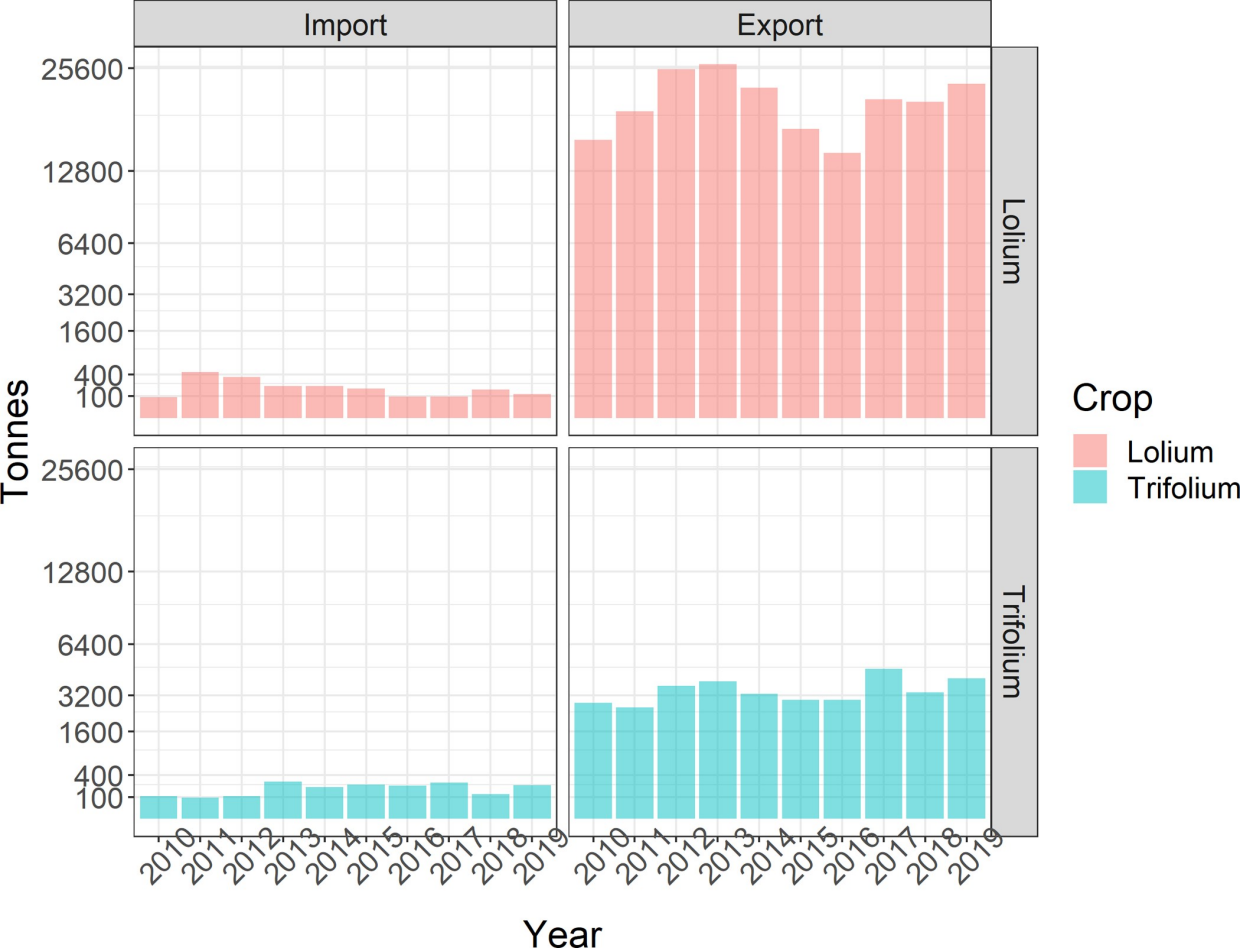
Tonnes of *Trifolium* spp. (clover) and *Lolium* spp. (ryegrass) traded with New Zealand based on the United Nations Comtrade Database.

We used the comtradr package for R [43] to download data from the United Nations Comtrade Database [42]. We collected data about trade volumes (in kilograms) for the forages ryegrass and clover under the commodity types with the descriptor “seeds, of a kind used for sowing” traded internationally for ten years (2010–2019). For subsequent modelling we used the annualized mean, but amounts varied from year-to-year e.g., Fig 1. We favoured data reported by New Zealand wherever the same (duplicate) trade transaction for a given year was reported with New Zealand but by another country as we could see no way to reconcile which was more accurate. Then we downloaded data for trades reported by New Zealand’s direct trading partners. If the total volume of trade reported was less than 1000 kgs over ten years we disregarded the trade amount as minor. Outside of the New Zealand reports, where more than one country reported on a trade relationship, we took the higher reported quantity as the true value. Some trade entities in the Comtrade database did not refer to country names, (e.g., “World”, “Special Categories”) and we did not use those in the network. The trade network was graphed using the R package ggraph [44] which uses a lot of the functions from the igraph package [45]. Trade volumes into and out of New Zealand are summarized from these data. The network edges were weighted by the mean annual volume of seed traded (from the ten-year dataset).

### 2.2 Border authority inspection data for weed seed contaminants arriving in New Zealand

In our efforts to understand seed contaminants, and model their spread, we examined weed seed contaminants that were detected moving through the network into New Zealand. Data from phytosanitary inspections of seed for sowing importations were obtained from the Ministry for Primary Industries in New Zealand from their “QuanCargo” database. We focus on a subset of data from the broader analysis carried out by Rubenstein et al [27], and provide some additional detail related to seed contaminants coming on the seed-for-sowing pathway for ryegrass and clover. These include graphs made in R using the tidyverse suite of packages related to the contamination rates for the countries New Zealand imports from [46,47].

### 2.3 A stochastic model of contaminant spread across the network

Utilizing the above-described network of ryegrass and clover seed trade (trade volumes in an average year) we created a stochastic time, network, susceptible-infected Markov chain model of contaminant spread, using our own R code, adapted from Andersen et al. [34]; the code is provided in the supplemental files. This involves deriving a matrix of probabilities, P, that a dispersal event/contaminant moves between trading partners in the network based on the average annual trade volume between the countries. For our model, the probability that a contaminant could move between nodes was calculated as 1-(1-contamination events/tonnes imported) for each trade event between any two countries from the Comtrade database [42]. (see S2 Fig in S1 File). The probability that a dispersal event/contaminant moves between any two trading partners (country nodes) i to j is p_ij_ the element in row i and column j of P. To set the probability of a dispersal between nodes we estimated the number of incursions and detections of regulated quarantine weeds at the border and estimated number of tonnes imported (using the average annualized volume of 199 and 193 tonnes imported for ryegrass and clover, respectively). Contamination events related to detections of seeds at the border and incursions into New Zealand are explained further below.

There was one detection of a regulated quarantine weed taxon for clover and three for ryegrass which we obtained from the QuanCargo data. For the incursions, we are referring to cases where weeds or seeds that had been detected in New Zealand after shipments had passed official checks and approvals at the border (see S2 Fig in S1 File). There was one incursion in 20 years for both ryegrass and clover. One incursion involved ryegrass seed contaminated with the weed blackgrass (*Alopecurus myosuroides* Huds) detected in seed destined for export from New Zealand in 2015 and again in 2016. From this information this problematic species was able to be traced to two seed lots imported from the United Kingdom in early 2007. In mid-2007, black-grass was removed from the Plant Biosecurity Index [this meant detections from then on were denied entry]. The detection of these contaminants forms part of an incursion response at a few Canterbury farms (unpublished data Ministry for Primary Industries). Also, a dodder, *Cuscuta pedicellata* Ledeb., not known to present in New Zealand, arrived in 2018 with a five-tonne shipment of berseem clover (*Trifolium alexandrinum* L.) and was sold for use in regenerative agriculture seed mixes, and then recalled [48], the problem species was not detected during border inspections, but by the seed company.

These detection and incursion rates served to set realistic generalized probability of dispersal for incursions and detections, related to trade volumes. We did not model fluctuations in volume, though our use of a stochastic model could mimic such patterns. A random uniform function (runif function in R) was used to generate a matrix N_t_ (numbers between zero and one), the network adjacency matrix at time t, of the same dimensions as the matrix describing the directed trade network, was compared against the probability matrix, to stochastically determine if a dispersal event occurs at each time step. A “starting state” row vector the same size as the number of rows of the network adjacency matrix tracks the infection status (1 for infected, 0 for uninfected), and each node is set as a starting point for simulations (the state of node j is in column j).). This vector s_t_ is modified by matrix multiplication s_t_N_t_ for each time step and then we track infected nodes, with the larger dimension matrix allowing us to track dispersal a similar process was described by Andersen Onofre et al. [35]. The state at the next time step is:

$$s_{t+1}<-\;as.numeric\left(s_{t}N_{t}>0\right).$$

This replaces positive values of s_t_N_t_ by 1, as s_t_N_t_ can have elements that are greater than 1 and leaves the zeros unchanged so that s_t+1_ is a row vector of 0s and 1s. Each node in the network was set as an “infected” starting point in the directed network, and 200 simulations run for ten time-steps. In practice 72 of 134 nodes (ryegrass) and 66 of 110 nodes (clover) had some outward trade and could be a source of spread.

We used the model to address the following questions: (i) Are biosecurity risk metrics that consider trade network topology and volume, different from risk metrics based on trade volume alone? To examine this, we ranked risk based on trade volume alone (deciles), and then compared these to deciles derived from counted dispersal events between nodes across the network for all simulations. Simulated spread between nodes should depend on node position and connectivity, not just volume traded. (ii) What is the risk posed to the countries in the ryegrass and clover seed trade network, when any given country is infected with a transmissible contaminant? Here we counted the number of countries infected per simulation. (iii) If a country node is “infected” with a contaminant what proportion of the risk is direct versus indirect from the point of view of a single country e.g., New Zealand? Here we counted dispersal events from simulation start-nodes direct to New Zealand, versus indirectly from shared trading partners.

## 3. Results

### 3.1 International trade networks for ryegrass and clover

**Fig 2:**
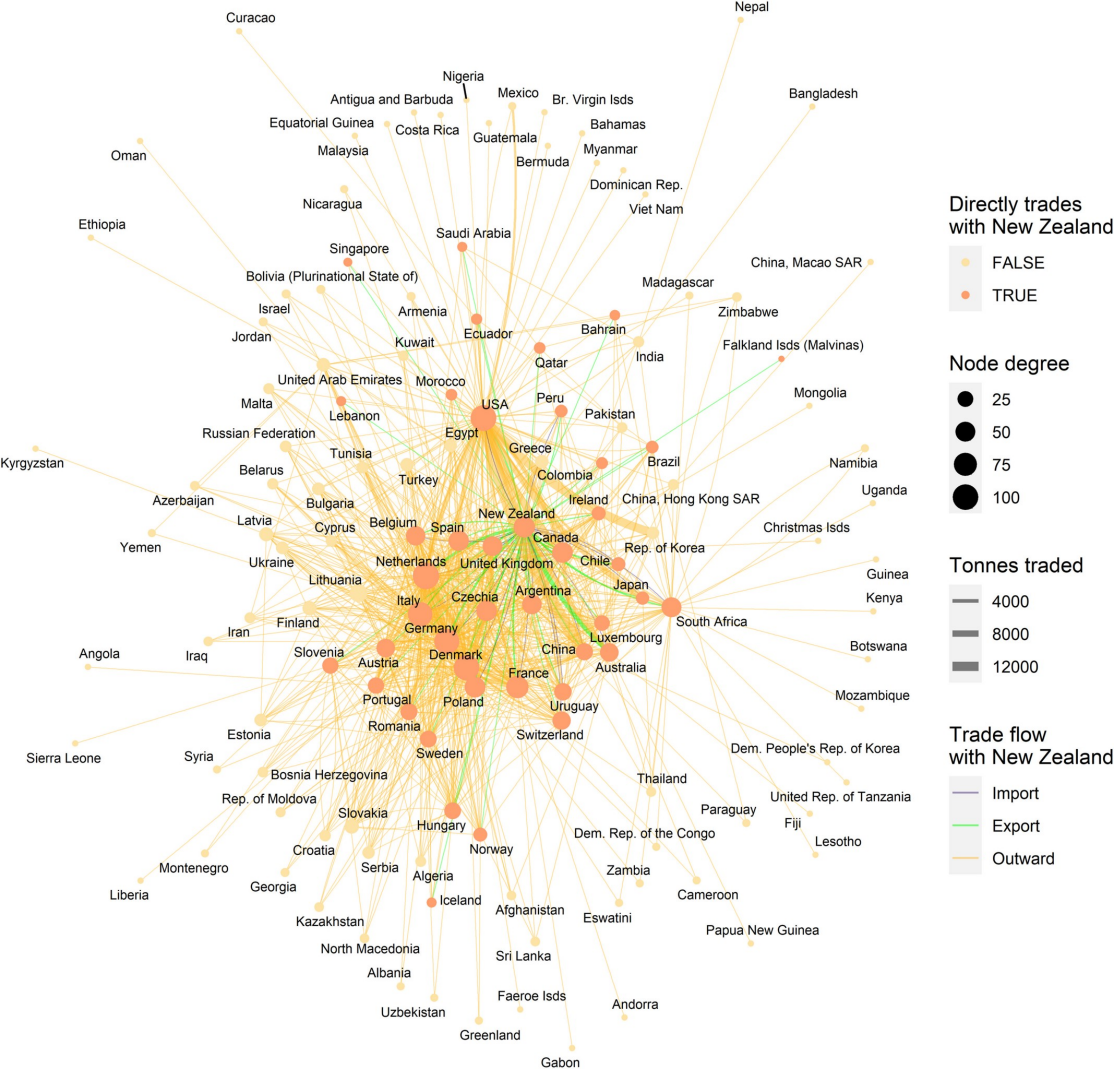
A trade network of ryegrass species traded as seed for sowing. This includes trades reported by New Zealand and those reported by its trading partners from the United Nations Comtrade Database. Node degree indicates the number of links per node. Link colours indicate imports (blue) and exports (green) to New Zealand while “outward” (orange) links indicate trades between other countries, width indicates trade volume in tonnes. Layout is a “stress” layout (ggraph R package). Node colours vary depending on whether the country trades directly with New Zealand.

**Fig 3:**
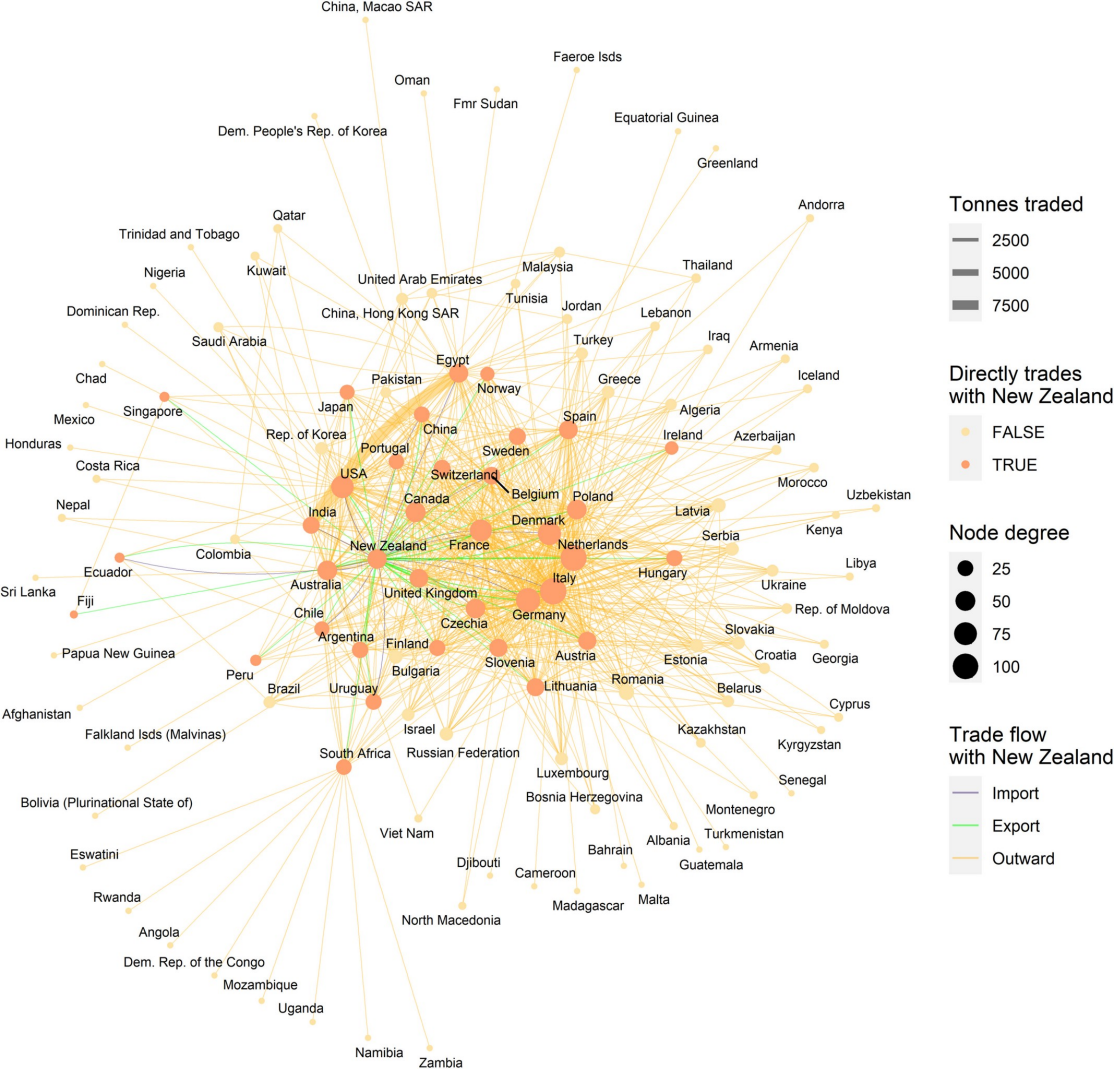
A trade network of clover species traded as seed for sowing. This includes trades reported by New Zealand and those reported by its trading partners from the United Nations Comtrade Database. Node degree indicates the number of links per node. Link colours indicate imports (blue) and exports (green) to New Zealand while “outward” (orange) links indicate trades between other countries, width indicates trade volume in tonnes. Layout is a “stress” layout (ggraph R package). Node colours vary depending on whether the country trades directly with New Zealand.

New Zealand imported an average of 199 tonnes (sd ±116) of ryegrass and 193 tonnes (sd ±75.6) of clover seed per annum (Fig 1) from a total of 23 and 16 countries, respectively (Figs 2 and 3). Trade networks of ryegrass and clover show average annual volumes for trade links where ≥1000 kgs were traded between any two countries in the period from 2010 to 2019 (Figs 2 and 3). In turn, New Zealand exported an average of 20,820 (sd ±3,838) tonnes of ryegrass (Figs 1 and 2) and 3,444 (sd ±671) tonnes of clover seed per annum to a total of 44 and 36 countries, respectively (Figs 1 and 3). The top five ryegrass exporting countries were the USA (71,599 tonnes per annum), Denmark (44,274), Netherlands (30,042), Germany (22,916) and New Zealand was fifth (over the same ten-year period). For clover, the top five exporters of were Egypt (11,789 tonnes), Italy (9,284 tonnes) USA (4,249), Canada (4,039), and 3,494 (Germany) only slightly ahead of New Zealand at sixth. The network shows a high-volume connection between India and Egypt–they trade mostly in Berseem clover (*Trifolium alexandrinum*). For ryegrass, New Zealand had 42 direct trading partners, with those trading partners’ connections bringing the network up to 134 countries (indirect trading partners). For direct traders the mean network indegree (number of inward connections) was 16.2, sd ±9.15 and out degree of 20.6 (sd ±21.3). For indirect traders (those not linked directly with New Zealand), the mean indegree was 3.3 (sd ±3.5), and outdegree of 1.3 (sd ±2.7), a network density of 0.086 and a graph diameter of 4. Sixty-two of the countries in the ryegrass network only had importations (0 outdegree) and so could not be a source of spread in stochastic simulations of contaminants. For clover, New Zealand had 35 direct trading partners, with their trading partners’ connections bringing the network nodes to 110 countries. For direct traders the mean indegree was 16.7 (sd ±10) and outdegree of 20.7 (sd ±16.2). For indirect traders, the mean indegree was 3.4(sd ±3.3) and outdegree of 1.5 (sd ±2.5), a network density of 0.105 and graph diameter of 5. There were 44 countries in the clover network which only had importations (0 outdegree) and so could not be a source of spread in subsequent spread models.

### 3.2 Border authority inspection data for weed seed contaminants arriving in New Zealand

**Fig 4:**
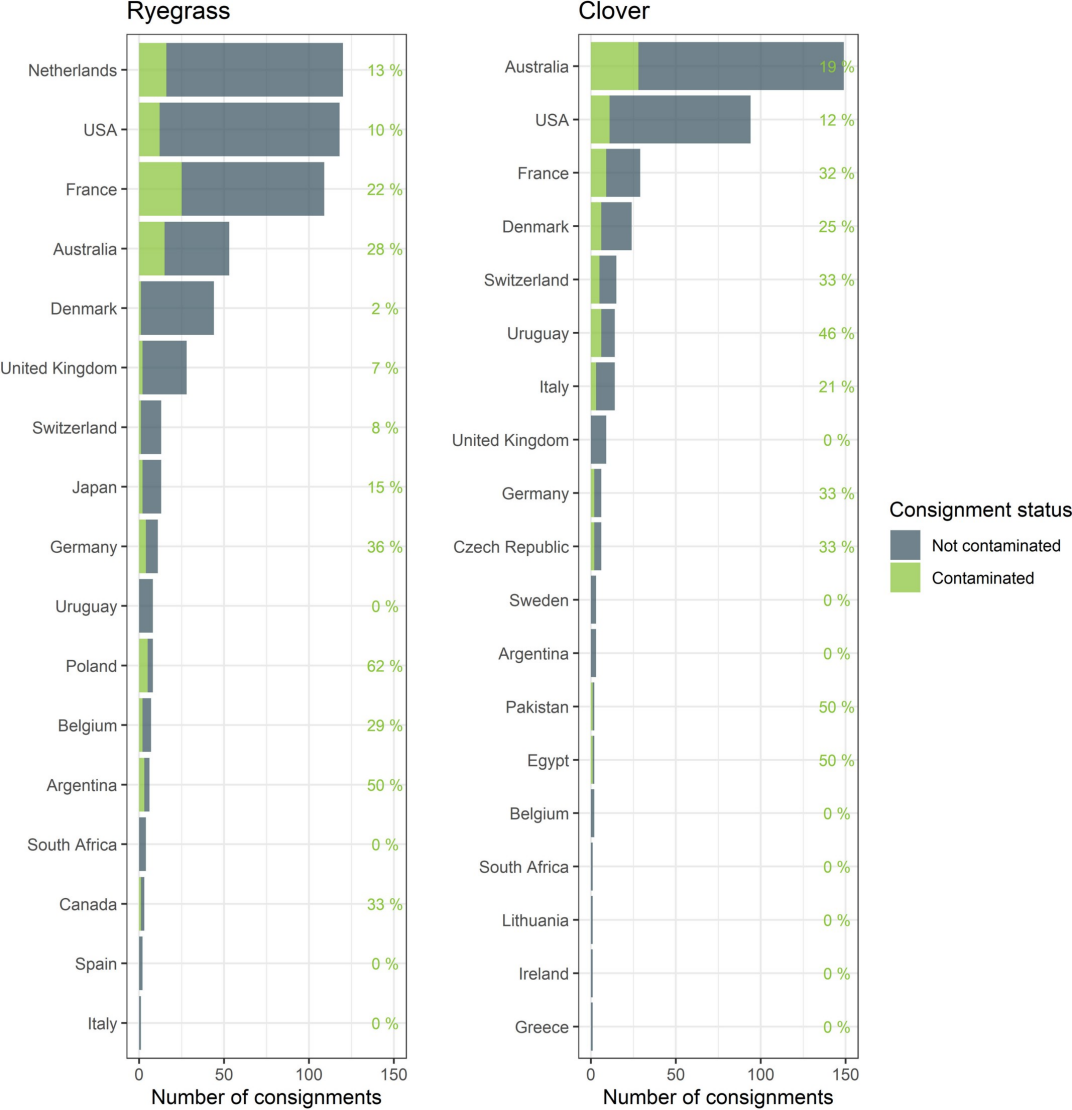
Percent contamination in seed lots of ryegrass and clover seed entering New Zealand from each importing country. This shows the number of seed lots of ryegrass and clover (n = 570 and 374, respectively), with percentage of lots contaminated per country (the overall contamination rate was 16 and 20%, respectively).

To put our modelling efforts into perspective we examined ryegrass and clover seed contaminants moving through the seed networks by looking at weed seed detections or incursions found in seed lots imported into New Zealand. Between 2014 and 2018, contaminants of both regulated and unregulated weed seeds for ryegrass were present in 15.9% (64 species) of 560 seed lots and 19.8% (60 species) of 374 clover seed lots, respectively. Only 20 of the species are shared between ryegrass and clover seed lots; with a total 105 taxa identified to species (S1 Table in S1 File). Ryegrass exporting countries with high contamination rates (and ≥10 seed lots) included Germany (36% of n = 11 had 7 species), Australia (28% of n = 54 seed lots contained 15 species in total), and France (22% of n = 114 had 35 species). Meanwhile for clover exporters Uruguay (46% of n = 13 had 22 contaminant species), Switzerland (33% of n = 10 had 9 species) and France (32% of n = 28 had 21 species). Despite a high number of importations, contamination rates were low for ryegrass from the Netherlands (13% of n = 120, 29 species) and the United States (10% of n = 121, 8 species) and for clover the United States (12% of n = 93, with 23 species), Fig 4. However, quarantine weeds were rarely detected. Over the period of 2014 to 2018 quarantine weeds were detected (and stopped at the border) in three separate shipments of ryegrass including *Alopecurus myosuroides* from France and Netherlands in 2018 and *Arctium minus* (Hill) Bernh. in 2018 from Poland, while in clover only *Carduus crispus* L. was detected in a shipment from Uruguay in 2017. The top five New Zealand importers accounted for 61% (ryegrass) and 55% (clover) of the imported seed lots, and four of these are top five importers for both clover and ryegrass (S1 Fig in S1 File).

### 3.3 A stochastic model of contaminant spread across the network

We carried out 200 simulations (each of which uses stochastic element for dispersal between nodes, in all 10 time-steps) to examine the spread of a hypothetical contaminant from every country in the network (only those with exports of ryegrass (72 countries) or clover (66 countries) could be a source. Here the probability of dispersal is based on the rate of incursions or detections per tonne of imported goods, as indicated by the number of incursion responses or detections of quarantine weeds from seed in New Zealand (S2 Fig in S1 File). We suggest that the incursion number represents a minimal estimate of the number of cases that might be expected to arrive and become problematic despite stringent border biosecurity measures that New Zealand implements. Simulations using the detection data should indicate the approximate number of quarantine weed incursions that might be prevented using the inspection standards that New Zealand has in place, this is because each detection resulted in border officials requesting mitigations in the form of seed cleaning, destruction or reshipment. The incursion data is a lower bound for the number of quarantine or high-risk species that are identified after passing through these inspections. These simulations run for ten time-steps and nominally represent ten years of potential spread (see caveats in the discussion). Examples of dispersal events for a single simulation are provided in S4 and S5 Figs in S1 File.

**Fig 5:**
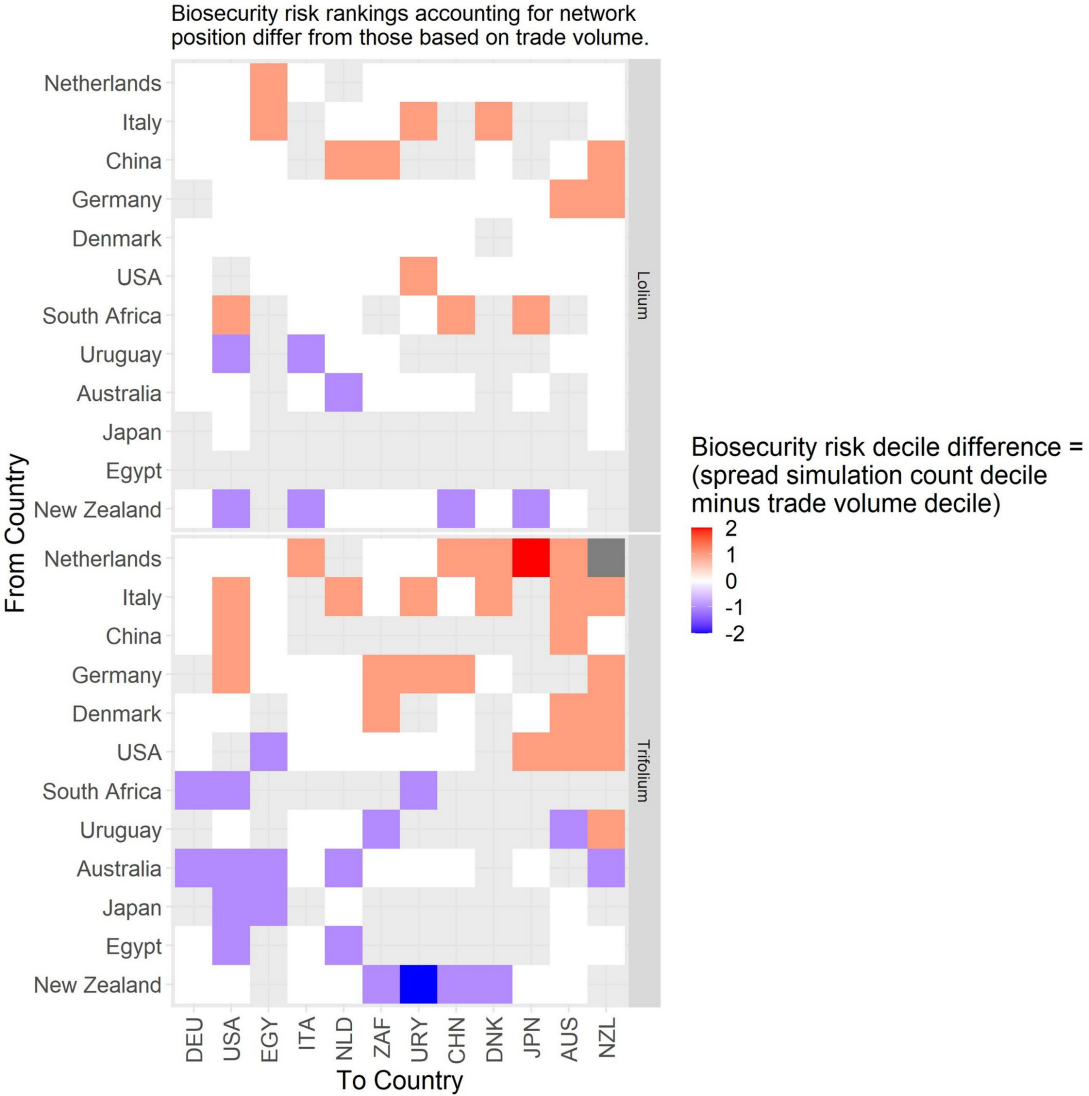
Biosecurity risk differences from network simulation versus trade volume assessments. We use decile rank differences for trade links from countries (y axis) to countries x axis) for a subset of countries derived from simulated dispersal events in the network model, as compared to risk estimated directly from trade volumes reported on the UN Comtrade Database. A grey background indicates there is no trade. Panels indicate the crop type. A similar figure for all the countries in the networks is available (S3 Fig in S1 File).

**Fig 6:**
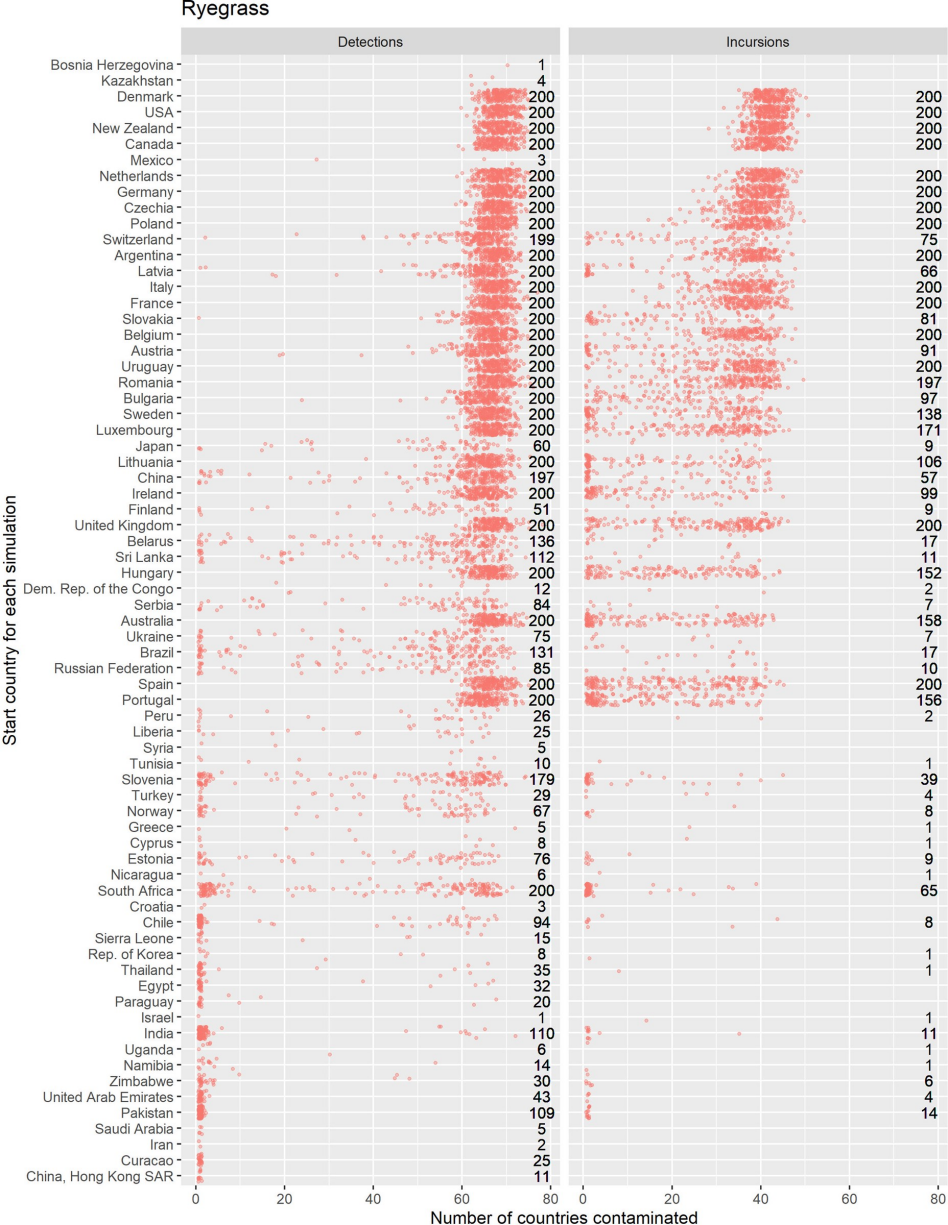
The number of countries contaminated after ten annual time-steps (x axis) using a stochastic model of spread for a hypothetical contaminant of ryegrass originating at the start country (displayed vertically). Jittered points are raw data from each simulation. The numbers to the right of each panel show the total number of simulations that resulted in dispersal out of a possible 200, each with 10 time-steps. The probability of spread is derived from the number of detections or incursions of quarantine weeds seen at the border in New Zealand (panel labels).

**Fig 7:**
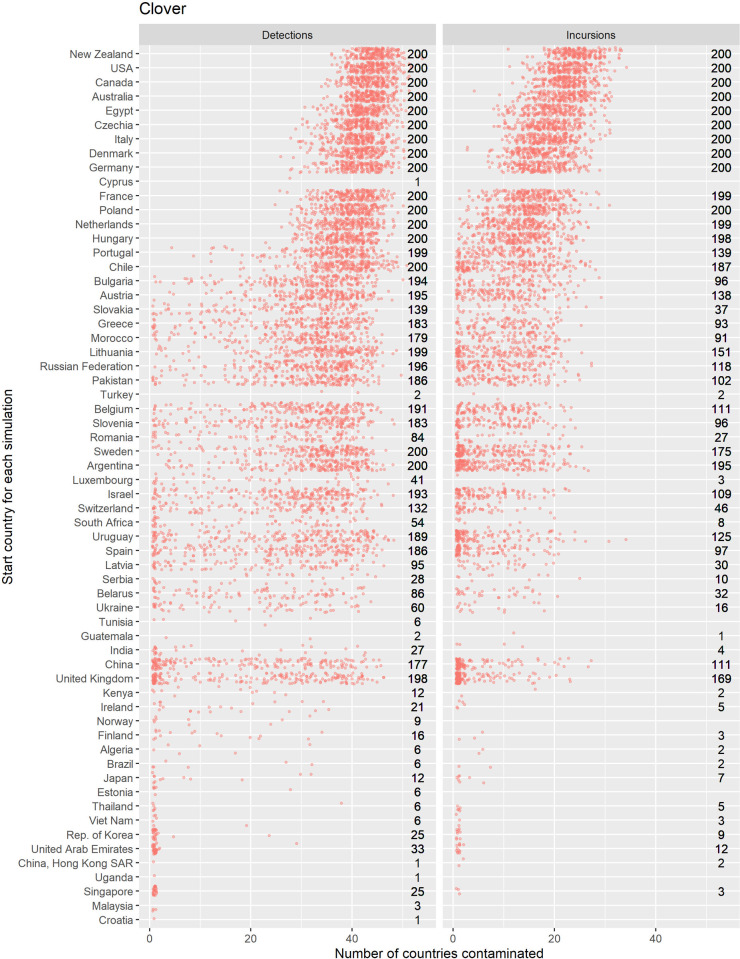
The number of countries contaminated after ten annual time-steps (x axis) using a stochastic model of spread for a hypothetical contaminant of clover originating at the start country (displayed vertically). Jittered points are raw data from each simulation. The numbers to the right of each panel show the total number of simulations that resulted in dispersal out of a possible 200, each with 10 time-steps. The probability of spread is derived from the number of detections or incursions of quarantine weeds seen at the border in New Zealand (panel labels).

**Fig 8:**
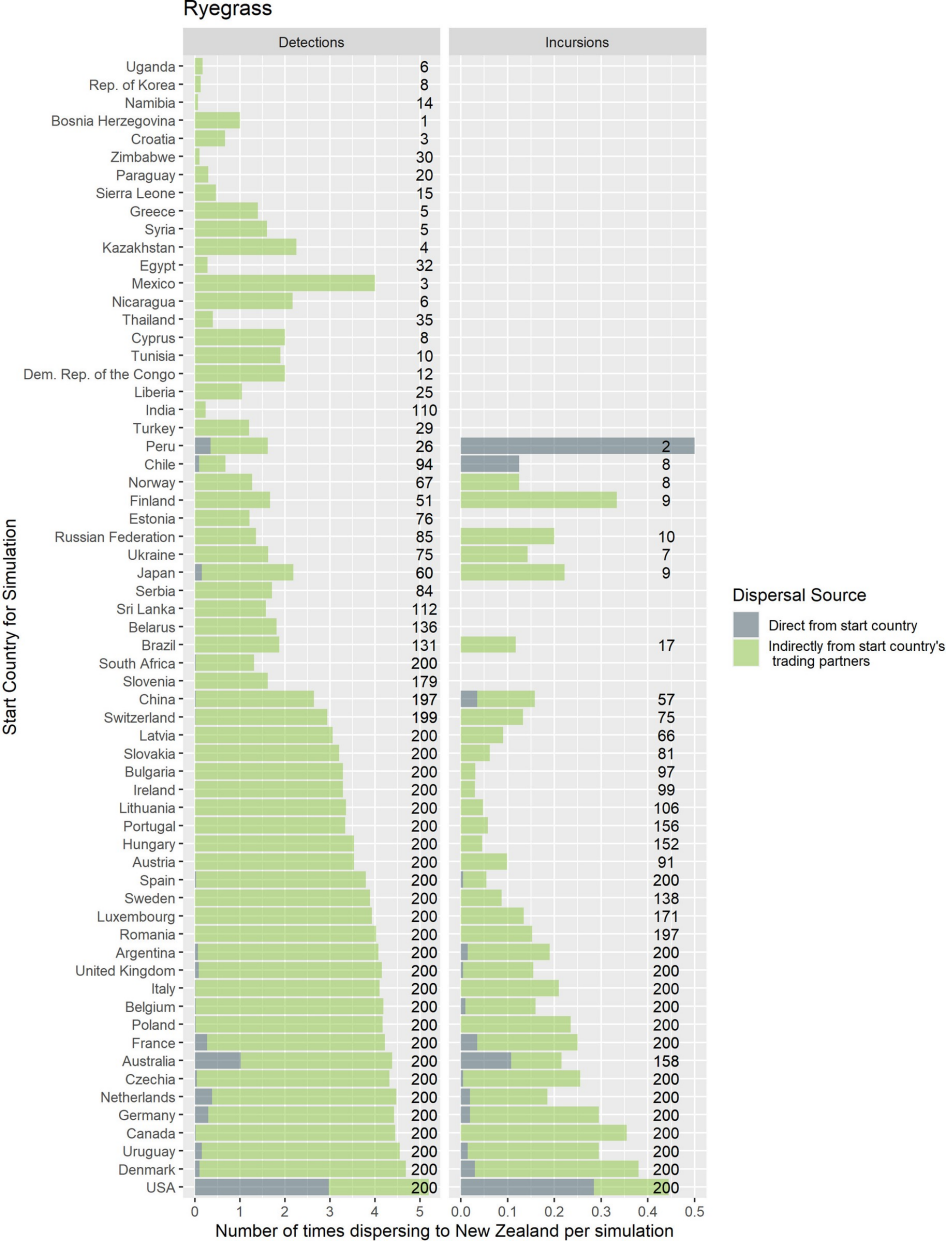
The number of times that a hypothetical contaminant of ryegrass arrives to New Zealand, after starting in each country. We distinguish cases where it occurs via a direct link from the country or indirectly via another country. Each simulation relies on a probability of spread derived from the number of detections or incursions of quarantine weeds seen at the border in New Zealand and the volume of trade between any two nodes (panel labels). The numbers (to the right of the stacked bars) show the total number of simulations that resulted in dispersal out of a possible 200.

**Fig 9:**
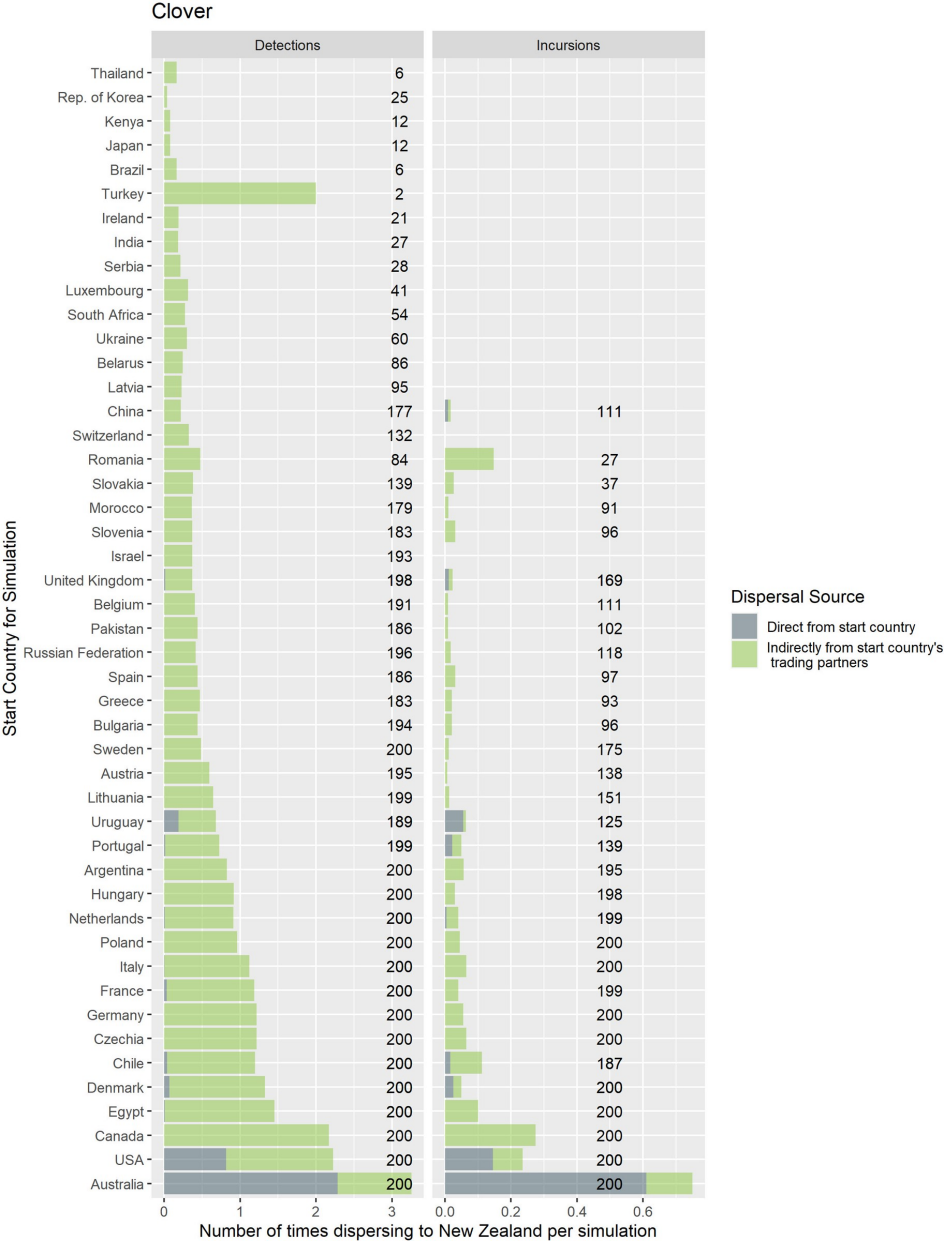
The number of times that a hypothetical contaminant of clover arrives to New Zealand, after starting in each country. We distinguish cases where it occurs via a direct link from the country or indirectly via another country. The probability of spread is derived from the number of detections or incursions of quarantine weeds seen at the border in New Zealand. The numbers (to the right of the stacked bars) show the total number of simulations that resulted in dispersal out of a possible 200.

For the questions we set out to answer with the simulations:

- Counts of the modelled dispersals from each country node were used as a metric for biosecurity risk it poses to its trade partners (where there were 200 simulations with 10 time-steps initiated for each exporting country). This means that for all the simulations where contamination was initiated in each starting country the minimum number of chances for a dispersal from that starting node was 2000, the upper bound depended stochastically on the number of countries contaminated at each time step. Deciles ranks of dispersal events were compared to the decile rank risk predicted from trade volume alone. For ryegrass and clover networks, respectively they differed 18% and 48% of the time, 10% and 28% were higher, while 8% and 20% were lower. The difference was rarely more than one decile difference (e.g., Fig 5 and S3 Fig in S1 File), but ranged from 1 to -4 for ryegrass and 3 to—4 for clover. We attribute differences to network topology and node position effects, not statistical or random effects. This pattern is well illustrated with a subset of countries for both the ryegrass and clover networks (Fig 5). The risk ranking (deciles) from simulations using the clover trade network varied more from the volume-based ranks for ryegrass, e.g., as much as 30% more for seed moving from New Zealand to Argentina and from France to New Zealand. Whereas for the same trade links for ryegrass, the simulated risk was in the same decile as volume-based risk estimates.
- A good summary of the per country biosecurity risk is provided in Fig 6 for ryegrass and Fig 7 for clover. This shows number of countries that become contaminated per simulation, as well as the number of successful simulations (out of the 200 we ran for each start country, running for ten time-steps) that resulted in dispersal events (these vary stochastically). The number of dispersal events is one basic measure of risk. For countries that are highly connected and with adequate volumes every simulation of ten time-steps results in the movement of a contaminant even at the low probability seen for incursions, e.g., for ryegrass Canada and USA, and for clover Australia, and Egypt. We see that New Zealand’s main trading partners are also major traders worldwide (Figs 2–4) and due to their many connections and high volumes, the spread of hypothetical contaminants from these countries will by chance reach a range of different countries. In the ryegrass example, when simulations start in New Zealand, the median number of countries contaminated per simulation ranged from 68 (detection rates) vs 42 (incursion rates), where the 90% quantile was 72 vs 45, the 10% quantile 65 vs 38 and the number of successful simulations was 200 in both the detection and incursion examples. Taking a different ryegrass case, where simulations start in Australia, the median number of countries contaminated per simulation when using the rates for detection versus incursions was 67 vs 22, while the 90% quantile was 70 vs 38, and the 10% quantile was 64 vs 1 and the number of successful simulations was 200 (detection rate) versus 145 (incursion rate).
- Taking a New Zealand-centric example, we see that many of the simulations starting in countries other than New Zealand could result in indirect dispersal of a contaminant to New Zealand (Figs 8 and 9). Here we have expressed this as the number of dispersal events per simulation (each of which had ten time-steps) averaged across all 200 of the simulations initiated. We also counted cases that were direct from the start country versus indirect and arrive from the start country’s trading partner. Indirect connections from a larger number of mostly European countries resulted in more dispersal events for ryegrass compared to clover. It is notable that the number of simulated incursions is roughly one tenth the number of detections for ryegrass (the x-axis range is different for incursions versus detections for Figs 8 and 9). This is expected given the eleven-fold difference in detections per tonne of seed versus incursions per tonne of seed imported. A similar pattern is seen for clover.

A detailed illustration of the number of countries contaminated and the potential for indirect spread can also be seen with a close look at single simulation (with ten time-steps, S4 & S5 Figs in S1 File). In an example of spread on the clover trade network, a contaminant is initiated in Lithuania and simulations show it arriving to 19 countries over ten time-steps (S4 Fig in S1 File). Then we see the role of indirect spread to New Zealand (NZ) where the contaminant moves from Lithuania to Germany (time-step 2), Germany to USA (time-step 6) then USA to NZ (time-step 7). In later steps it arrives in NZ from Ireland, Germany and Canada. A comparable example is provided for ryegrass (S5 Fig in S1 File).

## 4. Discussion

Quarantine weeds were rarely detected in ryegrass and clover seed lots imported into New Zealand [27]. Most seed lots had no non-crop seed contaminants detected, i.e., 84% of ryegrass and 80% of clover seed lots had no contaminants detected. This should be regarded as a positive, as it suggests seed production methods and cleaning efforts are keeping contaminant rates low. Given that sample sizes for inspection are five times higher in New Zealand than the ISTA rules require [5] detection rates should be proportionally higher than in countries relying on a single inspection at point of origin for any shipment. In practice, assuming random sampling and homogeneity, the amount of seed inspected per seed lot of any size would be approximately 150,000 seeds. Thus, for each inspection, under random sampling where no weed seeds were detected, authorities can be 95% certain that the contaminant rate is less than 1:50,000 seeds [30,49]. With the variability we see in contamination rates for countries exporting to New Zealand (Fig 4) we can assume that specific production and cleaning procedures could account for some of the differences [27]. Importers should work to identify and adopt the best performing seed production and cleaning technologies (as part of their own quality assurance system) to minimize contamination rates and weed pressure on their crops.

New Zealand had only one incursion response over the last 20 years for regulated weeds linked to each of ryegrass and clover seed imports. Further cases of introduction and/or establishment could possibly have occurred but still not be known. Presumably the establishment of new weeds post-border could be reported via the farmer a pest and disease hotline. New Zealand’s incursion responses for the weeds dodder (in clover seed) and blackgrass (in ryegrass seed) were put into motion after post-border seed inspections of seed lots destined for local use, or for export. For any shipment where no contaminant seeds are detected, there still could be quarantine species be present in the shipment, because by chance, they were not in the working sample. Bear in mind that inspected sample sizes for ryegrass and clover are the same for shipments from less than one kilo and on up to ten tonnes [5]. Hypothetically, if undetected weed seeds occurred at the difficult-to-detect rate of one contaminant per 100,000 seeds of the crop, more than 22% of the random samples of 150,000 seeds (this five times the ISTA recommended rate used in NZ) would be expected to be contaminant free. Such a rate could be quite consequential given the thousand seed weight of ryegrass (2 g) and clover (0.64 g), respectively [28,50]. Here, a ten-tonne shipment could contain as many as 50,000 (ryegrass) or 156,250 (clover) contaminants. At this contamination rate, and given the planting rates for pasture are 20 kgs (ryegrass) and 4 kgs (clover) per hectare, there is potentially non-negligible weed establishment since 100, or 62 seed contaminants per hectare, could be “sown” with the ryegrass and clover crops respectively [21]. Where seed is sown for multiplication, the sowing rates are similar. We could find no experiments about the rate at which regulated weed seeds are missed when they are present (e.g., after being experimentally added to a seed lot for inspection). This represents a poorly documented aspect of the risks inherent in the system.

While we model the role of incursions of rarely imported quarantine weeds, we note that there is a continuous flow of unregulated taxa (inferred to be low risk) through the network. Given the limited lists of quarantine weed species New Zealand and elsewhere the flow of common contaminant species between all the countries in the network must be significant. In just five years we see more than one hundred taxa arrived on this seed for sowing pathway for ryegrass and clover. It is unclear what the flow of common weeds and the concomitant exchange of genetic diversity could imply for invasion risks. It is possible that repeated introductions from across a species native range could increase an invasive species allelic diversity in its introduced range and that this could improve plant fitness so that invasive species impacts could increase over time [51]. In addition, specific alleles present overseas may be introduced in seed contaminants that are known to produce specifically known undesirable phenotypes. For example, it seems unavoidable that both ryegrass seed lots themselves and any contaminant weed species will contain herbicide resistant biotypes, a growing concern for the arable industry in New Zealand and elsewhere [52–57].

The rate of contamination in our is study is based on quarantine weed contaminants detected in NZ. Clearly the identity and number of species on the quarantine weed species list is an important consideration when modelling biosecurity risks, and these differ from country to country. As noted by previous authors almost half of the regulated species already occur in NZ [33]; we calculate that 47% (198 of the 419) of the taxa listed at the species level on the quarantine schedule are listed as naturalized in NZ [19]. However, a few of the taxa on the quarantine schedule [32] are listed at the level of genus or family (Viscaceae) and contain many species. In total, when the latter are included the New Zealand quarantine schedule potentially represents about ca. 1700 species. A large proportion of these quarantine species are unlikely to ever be contaminants in ryegrass or clover seed, or indeed any imported agricultural seed. It is unclear how often seed contaminants missed at border and later establish in New Zealand. Only a few species could easily be recognized as an incursion given the large number of introduced plants species and quarantine weeds known to occur in New Zealand. Our incursion rate estimate must therefore be an underestimate of the rate. For comparison in the United States there is a relatively small list of 112 species on the Federal Noxious Weeds list [58], Canada regulates just 18 species and two genera [59], and in Australia 15 taxa are listed at species, 32 at the level of genera but many have quite high contaminant tolerances e.g. *Melinis* spp. are restricted weeds where 250 seeds per kilogram of crop seed are tolerated [60].

We think our model’s value is in understanding infrequent human mediated dispersal of high biosecurity risk organisms, since common contaminants may be expected to flow through the network all the time. The stochastic network modelling approach is also useful for understanding the roles of direct and indirect risk of contaminant spread in the seed-for-sowing pathway, where trade volumes are known. Similar analyses could be applied to other taxonomic groups, pathways and commodities [15,61–63]. Our simulations showed that contaminants often spread to New Zealand indirectly, via shared trading partners as opposed to directly. Indirect spread was important even when simulation started with a trading partner that trades with us directly. It is surprising how complex the network is given that some might consider our example crops to be minor globally, and that a large proportion of the market must be controlled by relatively few seed companies. If managing biosecurity risk is a shared goal of the governments implementing phytosanitary risk management, then good international communication between agencies would provide benefits to all actors in the network.

We believe our model of spread of hypothetical contaminants across the ryegrass and clover trade networks serves to capture differences in risk between countries that specifically relate to trade volume, the number of trading connections and their position in the trade-network. This consideration of network topology allows us to imagine risks not simply as a bilateral problem, but as a multilateral issue, where agricultural producers can view themselves as members of a global community with shared concerns. However, some caveats need to be acknowledged with respect to the simplifications we used for the model. Stochastic dispersal between nodes is implemented at each time step. We suggest that each step could represent a year; we approximate this by using average annual trade volumes for the base network. In practice trade volumes vary from year to year for seed companies and countries. Considering time-steps to represent a year may be reasonable for ryegrass and clover seed multiplication because their export involves summer harvest and processing that is distinctly seasonal. But we also assume that once a contaminant arrives in a country it is permanently established and can disperse instantaneously, i.e., we do not consider metapopulation dynamics, or long-drawn-out establishment phases. We also used a single base detection and incursion rate linked to volumes to calculate the probability of dispersal between nodes across the whole network. This is derived from data related to one or more regulated species detected in New Zealand. In practice rates of contamination must vary temporally, and spatially for individual weed seed taxa or other pests. Also, the rate (and our probability of dispersal) reflects one or more contamination events for an overall volume of trade between countries, so that stochastically simulated dispersal events between nodes (in a time step) represent “at least” one event. This could only be improved if we modelled individual seed lots (along with temporal patterns in volume and numbers). However, we had to work with annual volume data for the trade network from the UN Comtrade Database. We do end up with potentially realistic spatial and temporal variation in detections and incursions via the stochastic node to node dispersal at each time step. For our purposes, i.e., assessing node risk, a single detection rate that captures multiple species of regulated weed seen during inspections in New Zealand is still informative. This simplification does still allow us to claim that we are modelling reasonably realistic frequencies for rare quarantine weeds generally but tells us nothing specific about any individual species of concern. We see the model as a means of identifying high-risk nodes and pathways for rare biosecurity threats that might originate anywhere in the trade network.

Follow up research could involve further scenario analyses using the existing network data such as those described as “impact network analyses” [64]. A per-seed lot model could be useful, since effectively the expected numbers of incursions could be counted. This network could be extended to link to in-country networks of seed movement, including destination farms, and consider certified and uncertified seed lots. In this way the network models could be extended to efficiently allocate monitoring resources for post-border detection of problem species [65]. It could be useful to experimentally test how often known regulated seeds are missed by seed inspectors in samples of high-risk crops. Detection rates, detectability and slippage are not well understood since current seed inspection literature seems to emphasize the likelihood that different sampling strategies will provide samples that *contain* contaminants [5,66]. This could inform possible rates of post-border incursion and direct our monitoring efforts. Risk management measures could be altered significantly if weed seed bank composition for seed multipliers was intermittently tested on farm (offshore). Potentially this could allow shipments sourced from sites free of regulated weed species to be shipped with lower inspection and mitigation measures. Investigations into the international movement of herbicide resistant biotypes through the seed for sowing pathway are also needed.

## 5. Conclusion

International seed systems are a high-risk pathway for the introduction of weeds [27,48,67], pests [68,69] and diseases [37]. However, even with stringent inspection efforts, only 16–20% of imported ryegrass and clover seed lots had any seed contaminants. Additionally, quarantine weeds were detected very infrequently coming into New Zealand. Of the 105 contaminant seed species detected, most would be allowed into the country because they are not listed on the quarantine schedule. Phytosanitary measures for seed lots in most countries focus on keeping out a small number of pests diseases and weed species but in the case of weeds many known weeds are allowed through. Multiple non-crop and crop seeds are dispersed through trade, and populations must share genes regularly throughout their introduced range (globally). This may release commonly imported weeds from genetic constraints and make them more invasive [51]. The effectiveness of on-farm production methods and seed cleaning must be a key factor in determining which species are dispersed through the network. Introducing international standards for seed production and cleaning, and tracking the effectiveness of different methods, could shed light on the risks for incursion and reduce contaminant seed spread across the system. There appears to be little information about the number of regulated or unregulated seeds/species that could be coming through the pathway undetected despite the inspections (slippage). Our network simulations of contaminant movements allowed us to identify nodes (countries) with higher and lower biosecurity risk than would be expected from the amount of seed traded (this should be relevant to pests and diseases of ryegrass and clover in addition to weeds). We also showed that indirect spread poses a significant biosecurity risk via shared trading partners e.g., European countries that do not directly trade with New Zealand.

## Supporting information

S1 File Supplementary figures and R markdown code. Supplementary Figures S1 to S5 and Supplementary Table 1 are provided here. A separate section provides an overview of the R code and markdown outputs.
(DOCX)

S2 File Anonymized clover and ryegrass seed lot inspection data from the Quancargo database. This is data from phytosanitary inspections of seed for sowing importations that were obtained from the Ministry for Primary Industries in New Zealand from their “QuanCargo” database. Filename used in R code: Ryegrass_and_clover_inspection.csv.
(CSV)

S3 File Data downloaded from the UN Comtrade database documenting trade in seed for sowing for ryegrass and clover. These data were used to generate the trade networks. Filename used in R code: From_to_super.csv.
(CSV)
