# Supplementary material for: The phytosanitary risks posed by seeds for sowing trade networks
Source: PLoS One. 2021 Nov 30;16(11):e0259912. doi: 10.1371/journal.pone.0259912 (PMC8631629; doi:10.1371/journal.pone.0259912)
Supplement: S1 File — Supplementary Figures S1 to S5 and Supplementary Table 1 are provided here. A separate section provides an overview of the R code and markdown outputs. (DOCX) [file pone.0259912.s001.docx]

# Supplementary Material

## Supplementary Data

**R-code markdown outputs follow the Supplemental Tables and Figures**

**Data files:**

Anonymized clover and ryegrass seed lot inspection data. Filename: Ryegrass_and_clover_inspection.csv

Data from the UN Comtrade database documenting trade in seed for sowing for various forages including ryegrass and clover. Filename: from_to_super.csv

## Supplemental Figures and Tables

**Supplementary Figures**


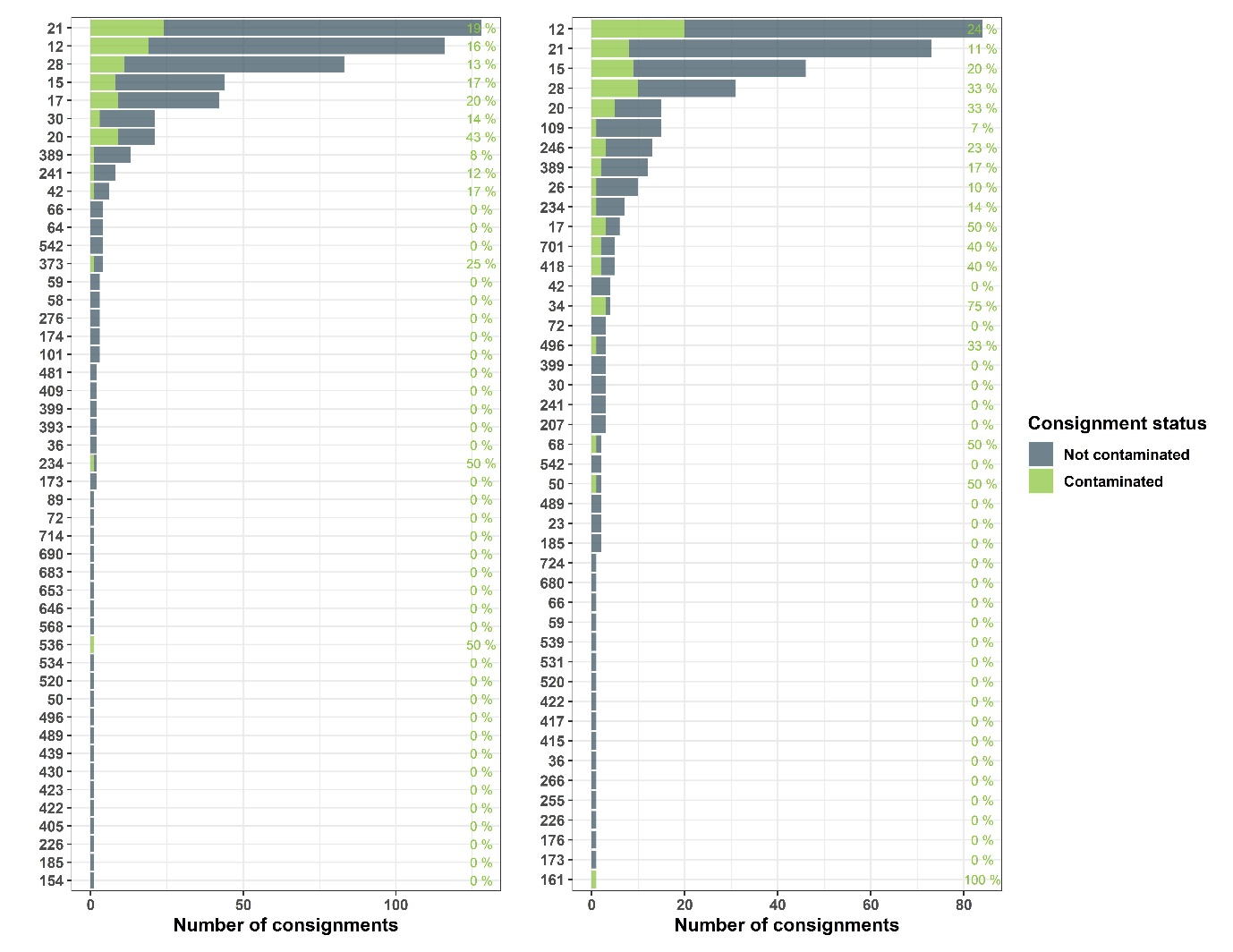


**Fig. S1.** For ryegrass (left) and clover (right) the number of consignments imported into New Zealand (n=570 and 374 respectively) and the percentage of consignments contaminated per importer (codes are shown); the overall contamination rate was 16 and 20% respectively.


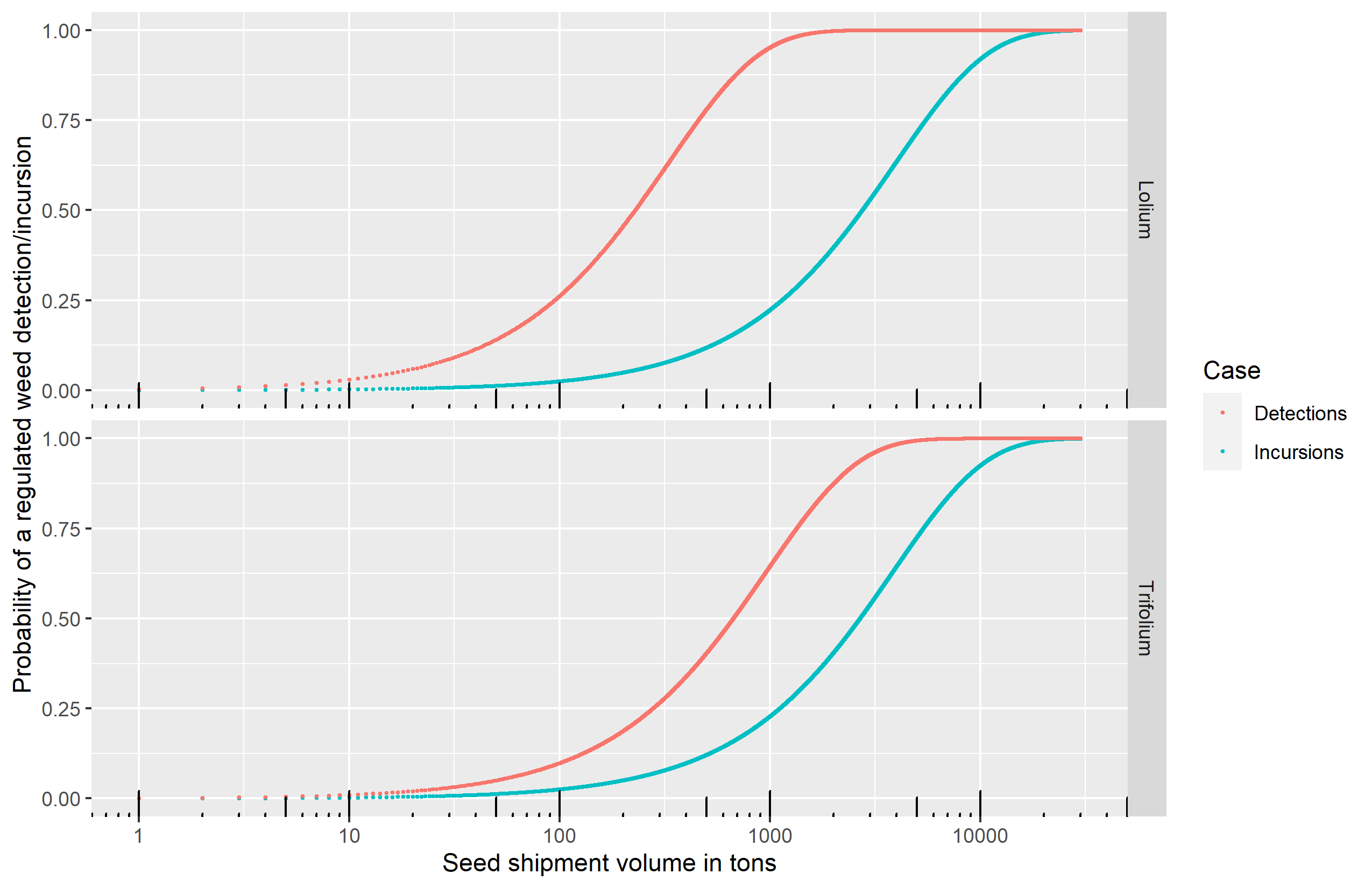


**Fig. S2**. For our model, the probability that a contaminant could move between nodes was calculated as 1-(1-contamination events/tonnes imported) ^tonnes imported^. Panels refer to the imported seed type. Clover (*Trifolium*) 1 detection over 5 years (2014-2018), 1 incursion over 20 years, average tonnes imported per year 193; ryegrass (*Lolium*) 3 detections over 5 years, 1 incursion over 20 years, average tonnes imported per year 199.


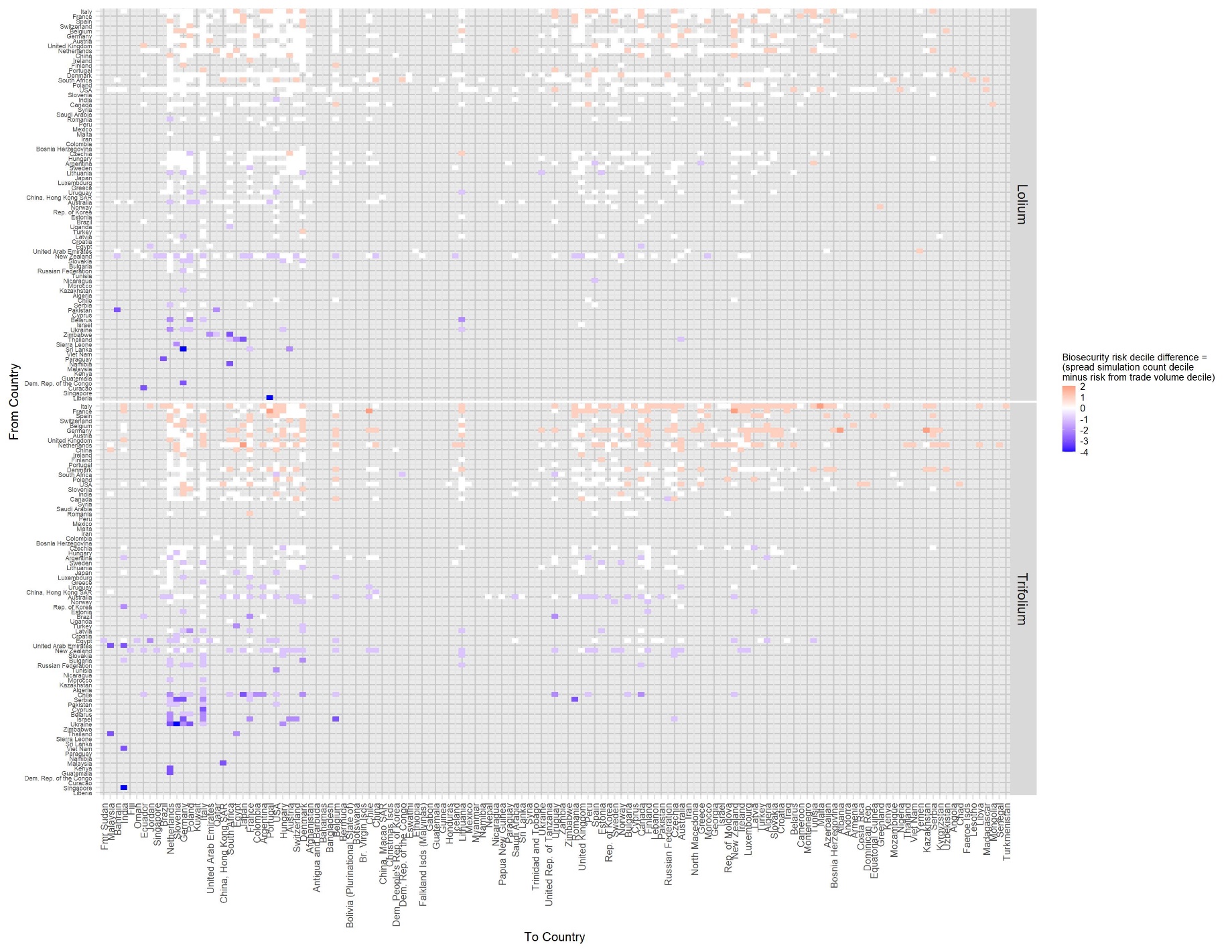


**Fig. S3.** Biosecurity risk rank differences for links between all of countries derived from simulated network dispersal events versus risk estimated directly from trade volumes reported on the UN Comtrade database.


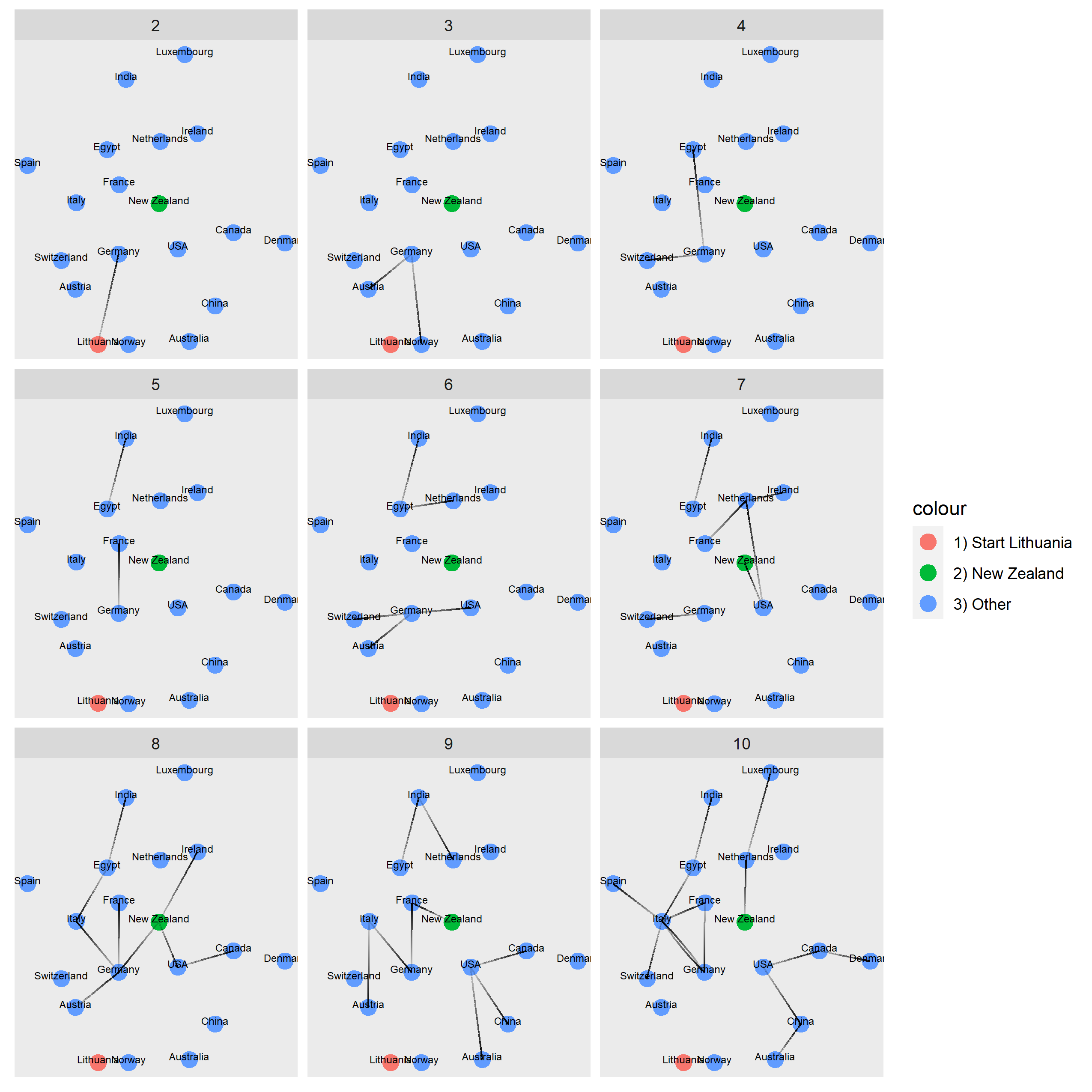


**Fig. S4.** An example of contaminant movements in a single simulation for clover incursions. Direction of spread is indicated from light to dark on the link. Every node stays infected once it arrives.


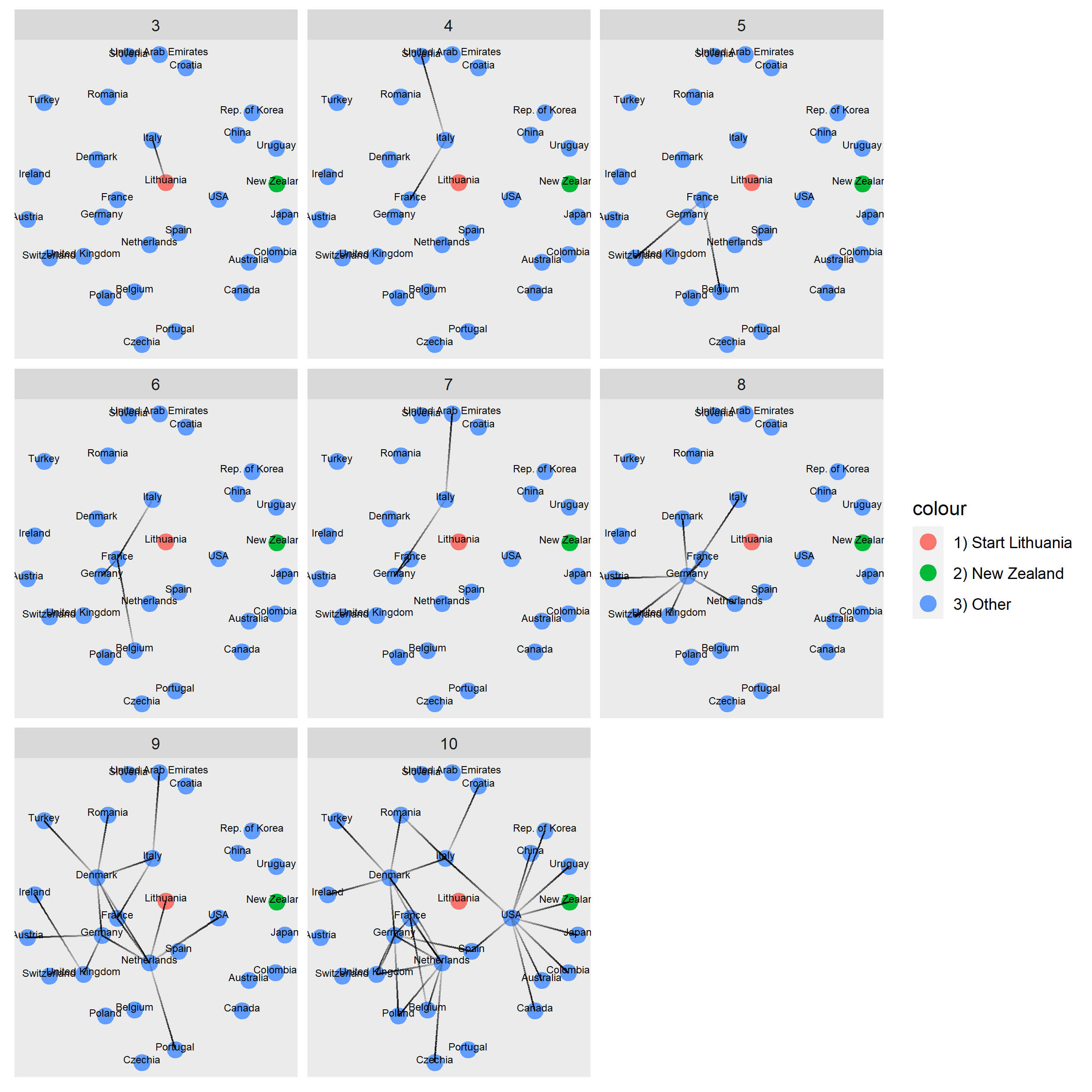


**Fig. S5.** An example of contaminant movements in a single simulation where the contaminant is initially found in Lithuania for ryegrass incursions.

**Supplementary Tables**

**Supplemental Table 1**. The number of times a contaminant species was found in ryegrass (*Lolium* spp.) and clover (*Trifolium* spp.) seed consignments of imported into New Zealand between 2014 and 2018 out of 560 ryegrass and 374 clover consignments. The Weed Regulation Status is from the Plant Biosecurity Index.

| No. | Contaminant Species | Weed Regulation Status | Lolium | Trifolium |
| --- | --- | --- | --- | --- |
| 1 | *Aira caryophyllea* | Basic | 2 | 0 |
| 2 | *Amaranthus retroflexus* | Basic | 0 | 4 |
| 3 | *Ammi majus* | Basic | 0 | 4 |
| 4 | *Anagallis arvensis* | Basic | 0 | 1 |
| 5 | *Anthemis arvensis* | Basic | 2 | 0 |
| 6 | *Anthoxanthum odoratum* | Basic | 1 | 0 |
| 7 | *Atriplex patula* | Basic | 1 | 0 |
| 8 | *Centaurea cyanus* | Basic | 1 | 1 |
| 9 | *Cerastium glomeratum* | Basic | 1 | 0 |
| 10 | *Chenopodium album* | Basic | 6 | 15 |
| 11 | *Cichorium intybus* | Basic | 0 | 2 |
| 12 | *Cirsium arvense* | Basic | 2 | 0 |
| 13 | *Cirsium vulgare* | Basic | 0 | 5 |
| 14 | *Cynosurus echinatus* | Basic | 1 | 0 |
| 15 | *Dracocephalum parviflorum* | Basic | 1 | 0 |
| 16 | *Eleusine indica* | Basic | 0 | 1 |
| 17 | *Fagopyrum esculentum* | Basic | 0 | 1 |
| 18 | *Fallopia convolvulus* | Basic | 2 | 0 |
| 19 | *Galium aparine* | Basic | 5 | 0 |
| 20 | *Geranium dissectum* | Basic | 2 | 0 |
| 21 | *Geranium pusillum* | Basic | 0 | 1 |
| 22 | *Glyceria fluitans* | Basic | 1 | 0 |
| 23 | *Helminthotheca echioides* | Basic | 0 | 2 |
| 24 | *Lamium purpureum* | Basic | 2 | 0 |
| 25 | *Lapsana communis* | Basic | 1 | 1 |
| 26 | *Lithospermum arvense* | Basic | 1 | 0 |
| 27 | *Lythrum hyssopifolia* | Basic | 0 | 2 |
| 28 | *Marrubium vulgare* | Basic | 1 | 0 |
| 29 | *Melilotus albus* | Basic | 1 | 0 |
| 30 | *Myosotis arvensis* | Basic | 0 | 1 |
| 31 | *Persicaria lapathifolia* | Basic | 5 | 3 |
| 32 | *Persicaria maculosa* | Basic | 5 | 2 |
| 33 | *Phacelia tancetifolia* | Basic | 1 | 0 |
| 34 | *Picris hieracioides* | Basic | 0 | 1 |
| 35 | *Plantago lanceolata* | Basic | 0 | 9 |
| 36 | *Polygonum aviculare* | Basic | 19 | 6 |
| 37 | *Ranunculus parviflorus* | Basic | 5 | 0 |
| 38 | *Raphanus sativus* | Basic | 0 | 1 |
| 39 | *Reseda lutea* | Basic | 0 | 1 |
| 40 | *Rumex acetosella* | Basic | 1 | 0 |
| 41 | *Rumex crispus* | Basic | 3 | 5 |
| 42 | *Rumex obtusifolius* | Basic | 3 | 1 |
| 43 | *Sherardia arvensis* | Basic | 4 | 1 |
| 44 | *Silene gallica* | Basic | 1 | 0 |
| 45 | *Silene latifolia* | Basic | 0 | 1 |
| 46 | *Sinapis alba* | Basic | 1 | 0 |
| 47 | *Sonchus arvensis* | Basic | 1 | 0 |
| 48 | *Spinacia oleracea* | Basic | 0 | 1 |
| 49 | *Stellaria media* | Basic | 6 | 3 |
| 50 | *Taraxacum officinale* | Basic | 1 | 0 |
| 51 | *Thlaspi arvense* | Basic | 0 | 1 |
| 52 | *Trifolium glomeratum* | Basic | 0 | 1 |
| 53 | *Trifolium hybridum* | Basic | 0 | 2 |
| 54 | *Trifolium pratense* | Basic | 0 | 5 |
| 55 | *Trifolium repens* | Basic | 5 | 1 |
| 56 | *Trifolium resupinatum* | Basic | 0 | 4 |
| 57 | *Trifolium striatum* | Basic | 0 | 1 |
| 58 | *Trifolium subterraneum* | Basic | 0 | 1 |
| 59 | *Tripleurospermum inodorum* | Basic | 2 | 0 |
| 60 | *Veronica persica* | Basic | 1 | 0 |
| 61 | *Alopecurus myosuroides* | Entry prohibited | 2 | 0 |
| 62 | *Arctium minus* | Entry prohibited | 1 | 0 |
| 63 | *Carduus crispus* | Entry prohibited | 0 | 1 |
| 64 | *Apera spica-venti* | Not Listed | 1 | 0 |
| 65 | *Silene media* | Not Listed | 0 | 1 |
| 66 | *Alopecurus geniculatus* | Phytosanitary Requirement | 4 | 0 |
| 67 | *Anthriscus caucalis* | Phytosanitary Requirement | 1 | 0 |
| 68 | *Avena fatua* | Phytosanitary Requirement | 1 | 0 |
| 69 | *Avena sativa* | Phytosanitary Requirement | 1 | 1 |
| 70 | *Bromus catharticus* | Phytosanitary Requirement | 0 | 1 |
| 71 | *Bromus hordeaceus* | Phytosanitary Requirement | 6 | 0 |
| 72 | *Bromus sterilis* | Phytosanitary Requirement | 3 | 0 |
| 73 | *Coriandrum sativum* | Phytosanitary Requirement | 1 | 0 |
| 74 | *Dactylis glomerata* | Phytosanitary Requirement | 1 | 0 |
| 75 | *Echinochloa crus-galli* | Phytosanitary Requirement | 9 | 1 |
| 76 | *Festuca arundinacea* | Phytosanitary Requirement | 0 | 2 |
| 77 | *Festuca rubra* | Phytosanitary Requirement | 2 | 3 |
| 78 | *Holcus lanatus* | Phytosanitary Requirement | 1 | 0 |
| 79 | *Hordeum murinum* | Phytosanitary Requirement | 0 | 1 |
| 80 | *Linum usitatissimum* | Phytosanitary Requirement | 3 | 0 |
| 81 | *Lolium perenne* | Phytosanitary Requirement | 0 | 1 |
| 82 | *Lotus corniculatus* | Phytosanitary Requirement | 0 | 1 |
| 83 | *Medicago lupulina* | Phytosanitary Requirement | 0 | 1 |
| 84 | *Medicago polymorpha* | Phytosanitary Requirement | 0 | 1 |
| 85 | *Medicago sativa* | Phytosanitary Requirement | 5 | 2 |
| 86 | *Panicum capillare* | Phytosanitary Requirement | 0 | 5 |
| 87 | *Panicum miliaceum* | Phytosanitary Requirement | 2 | 0 |
| 88 | *Phalaris aquatica* | Phytosanitary Requirement | 1 | 1 |
| 89 | *Phalaris arundinacea* | Phytosanitary Requirement | 0 | 1 |
| 90 | *Phalaris minor* | Phytosanitary Requirement | 0 | 1 |
| 91 | *Phalaris paradoxa* | Phytosanitary Requirement | 0 | 7 |
| 92 | *Phleum pratense* | Phytosanitary Requirement | 3 | 1 |
| 93 | *Poa annua* | Phytosanitary Requirement | 10 | 1 |
| 94 | *Poa pratensis* | Phytosanitary Requirement | 4 | 4 |
| 95 | *Poa trivialis* | Phytosanitary Requirement | 4 | 0 |
| 96 | *Rubus fruticosus* | Phytosanitary Requirement | 1 | 0 |
| 97 | *Secale cereale* | Phytosanitary Requirement | 0 | 1 |
| 98 | *Setaria viridis* | Phytosanitary Requirement | 1 | 0 |
| 99 | *Trigonella foenumgraecum* | Phytosanitary Requirement | 0 | 1 |
| 100 | *Triticum aestivum* | Phytosanitary Requirement | 2 | 0 |
| 101 | *Vicia sativa* | Phytosanitary Requirement | 2 | 0 |
| 102 | *Vulpia bromoides* | Phytosanitary Requirement | 3 | 1 |
| 103 | *xTriticosecale* | Phytosanitary Requirement | 0 | 1 |
| 104 | *Solanum nigrum* | Requires Assessment | 1 | 0 |
| 105 | *Agropyron sp.* | Genus or Family Level ID | 0 | 1 |
| 106 | *Amaranthus sp.* | Genus or Family Level ID | 1 | 4 |
| 107 | *Apiaceae sp.* | Genus or Family Level ID | 1 | 1 |
| 108 | *Asteraceae sp.* | Genus or Family Level ID | 0 | 2 |
| 109 | *Avena sp.* | Genus or Family Level ID | 0 | 1 |
| 110 | *Betulaceae sp.* | Genus or Family Level ID | 1 | 0 |
| 111 | *Brassica sp.* | Genus or Family Level ID | 4 | 4 |
| 112 | *Brassicaceae sp.* | Genus or Family Level ID | 1 | 0 |
| 113 | *Bromus sp.* | Genus or Family Level ID | 0 | 1 |
| 114 | *Chenopodium sp.* | Genus or Family Level ID | 2 | 2 |
| 115 | *Echinochloa sp.* | Genus or Family Level ID | 0 | 2 |
| 116 | *Elytrigia sp.* | Genus or Family Level ID | 1 | 0 |
| 117 | *Festuca sp.* | Genus or Family Level ID | 1 | 0 |
| 118 | *Galium sp.* | Genus or Family Level ID | 0 | 2 |
| 119 | *Glyceria sp.* | Genus or Family Level ID | 0 | 1 |
| 120 | *Limnanthes sp.* | Genus or Family Level ID | 1 | 0 |
| 121 | *Lobularia sp.* | Genus or Family Level ID | 1 | 0 |
| 122 | *Lolium sp.* | Genus or Family Level ID | 3 | 17 |
| 123 | *Lupinus sp.* | Genus or Family Level ID | 1 | 0 |
| 124 | *Medicago sp.* | Genus or Family Level ID | 0 | 2 |
| 125 | *Panicum sp.* | Genus or Family Level ID | 0 | 4 |
| 126 | *Phalaris sp.* | Genus or Family Level ID | 1 | 1 |
| 127 | *Poa sp.* | Genus or Family Level ID | 0 | 2 |
| 128 | *Rosaceae sp.* | Genus or Family Level ID | 1 | 0 |
| 129 | *Rumex sp.* | Genus or Family Level ID | 0 | 1 |
| 130 | *Silene sp.* | Genus or Family Level ID | 0 | 1 |
| 131 | *Sorghum sp.* | Genus or Family Level ID | 0 | 1 |
| 132 | *Unknown species* | Genus or Family Level ID | 0 | 1 |
| 133 | *Veronica sp.* | Genus or Family Level ID | 1 | 0 |
| 134 | *Vicia sp.* | Genus or Family Level ID | 2 | 1 |
| 135 | *Viola sp.* | Genus or Family Level ID | 1 | 1 |

## Overview of R-code analysis steps.

R markdown files were created to illustrate the main steps involved in the analysis. Outputs from those follow this brief description of the steps involved.

### 1) UN Comtrade data analysis

Comtrader database summary of traded volumes for ryegrass and clover, as well as the generation of trade network figures and adjacency matrices for later use in simulations. (Generates Figs 1-3) Requires the provided S3 supplementary file “from_to_super.csv”. Generates files (“forNet_ryegrass.csv”, "forNet_clover.csv" , “Lolium _node_stats.csv”, “Trifolium _node_stats.csv”, “net_adj_ryegrass.csv”, “net_adj_ryegrass.rds”, “net_adj_clover.csv", “net_adj_clover.rds", “trifolium_df2.rds”, “lolium_df2.rds”).

### 2) Analyze NZ border inspection contaminant data for ryegrass and clover

Examination of five years of New Zealand Border inspection data (generates Fig 4). Requires the provided S2 supplementary file “Ryegrass_and_clover_inspection.csv”. Generates files: “ContaminantSppCountries.csv”, “ContaminantSpp.csv”, “ContaminantSppCountries.csv”).

### 3) Ryegrass hypothetical contaminant spread simulations.

Visualize the probability relationship between biosecurity detections and incursions (one or more instance) and the annual trade volume. Run simulations. Every simulation will be different due to the stochastic elements of the model. They take a while (20-30 minutes in this case) with large networks and produce large csv files of all the simulations. Requires the files generated in step 1 from the Comtrade data (“net_adj_ryegrass.rds”, “lolium_df2.rds”). Generates files (“ryegrass_sim_incursion.csv”, “ryegrass_graph_list_incursion.rds”, “ryegrass_detection_sim.csv”, “ryegrass_graph_list_detection.rds”). The output files I generated are too big to provide and would be unique for each simulation.

### 4) Clover hypothetical contaminant spread simulations.

Visualize the probability relationship between biosecurity detections and incursions (one or more instance) and the annual trade volume. Run simulations. Requires the files generated in step 1 from the Comtrade data (“net_adj_clover.rds", “trifolium_df2.rds”). Generates (“clover_incursions_sim.csv”, “clover_graph_list_incursions.rds”, “clover_detection_sim.csv”, “clover_detection_graph_list.rds”). The output files I generated are too big to provide and would be unique for each simulation.

### 5) Heat map comparing simulation outputs to expected risk from trade volumes

Heat map data preparation and figure (Figure 5 plus supplementary Fig S3). Requires the previously generated simulation and Comtrade output files (“ryegrass_sim_incursion.csv”, “ryegrass_detection_sim.csv”,“Lolium _node_stats.csv”, “clover_incursions_sim.csv”, “clover_detection_sim.csv”, “Trifolium _node_stats.csv”)

### 6) Other outputs from the simulations

Generates extra figures from simulation outputs, Figs 6-9. Summarize outputs from the simulations. Requires the previously generated (“ryegrass_sim_incursion.csv”, “ryegrass_detection_sim.csv”,“Lolium _node_stats.csv”, “clover_incursions_sim.csv”, “clover_detection_sim.csv”, “Trifolium _node_stats.csv”)

## R code for the steps

### UN Comtrade data analysis


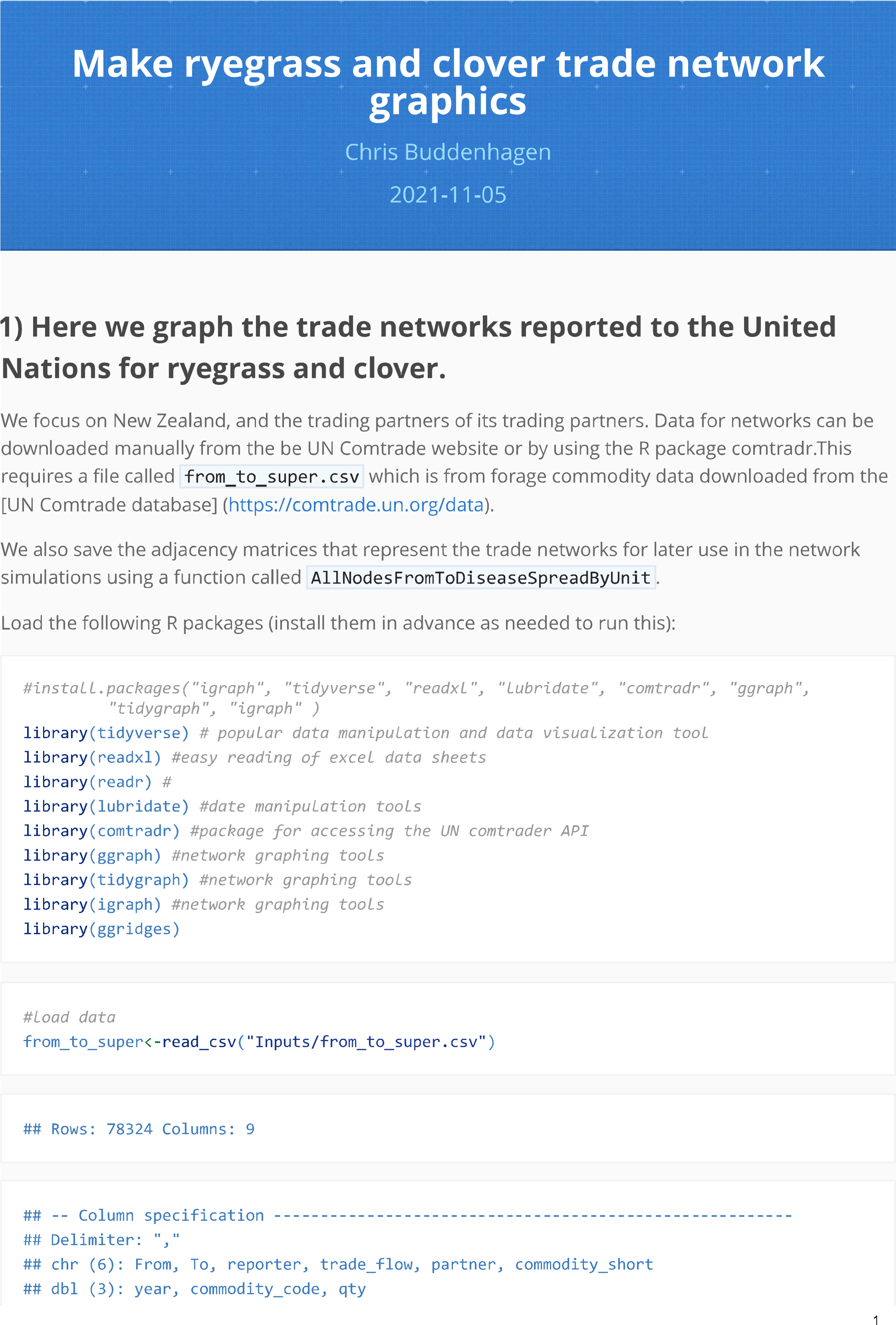


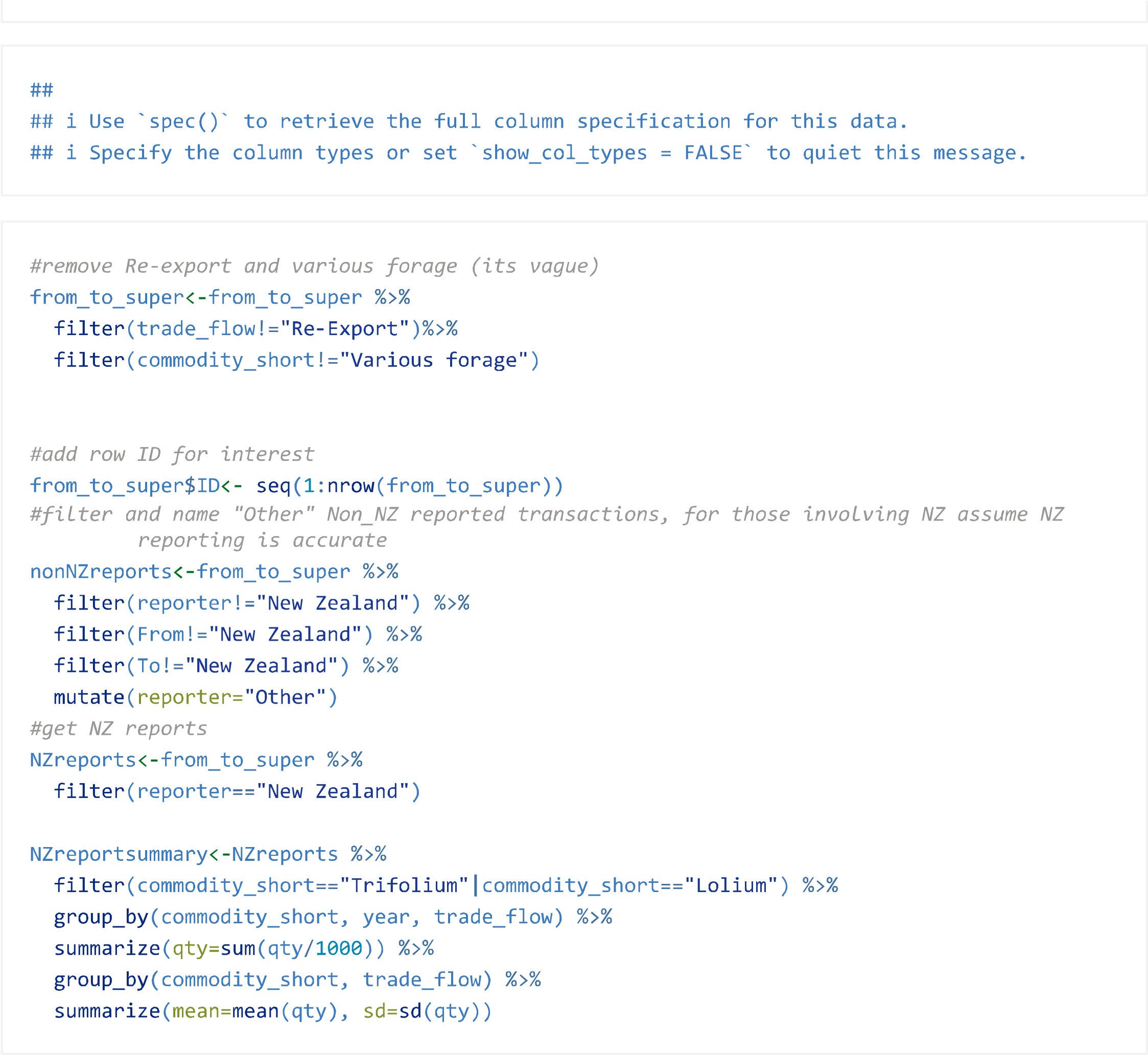


|  | | |
| --- | --- | --- |
|  | 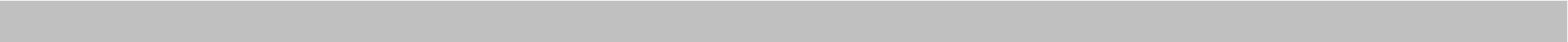 |  |
|  | | |
|  | 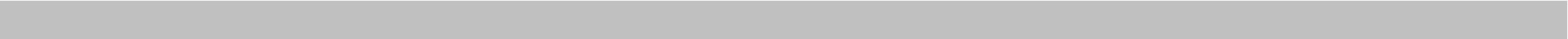 |  |


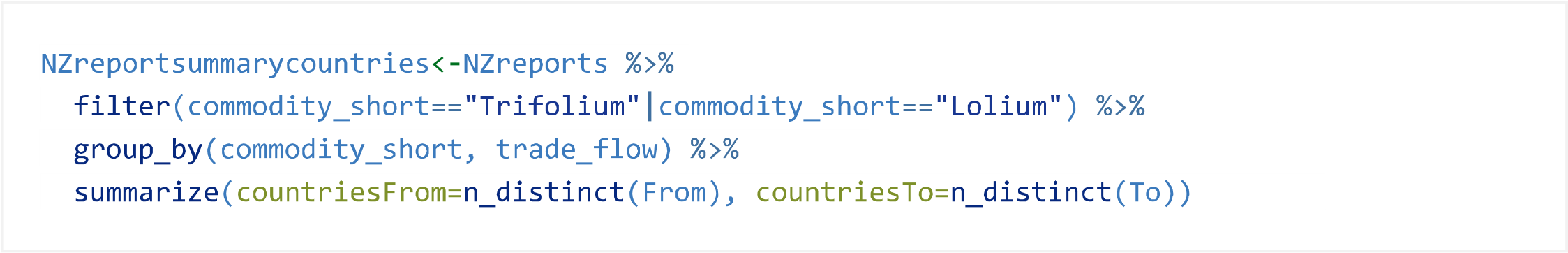


|  |
| --- |
| 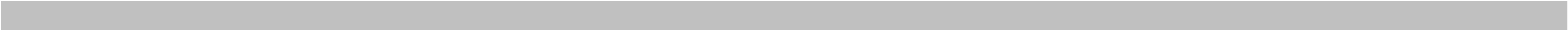 |


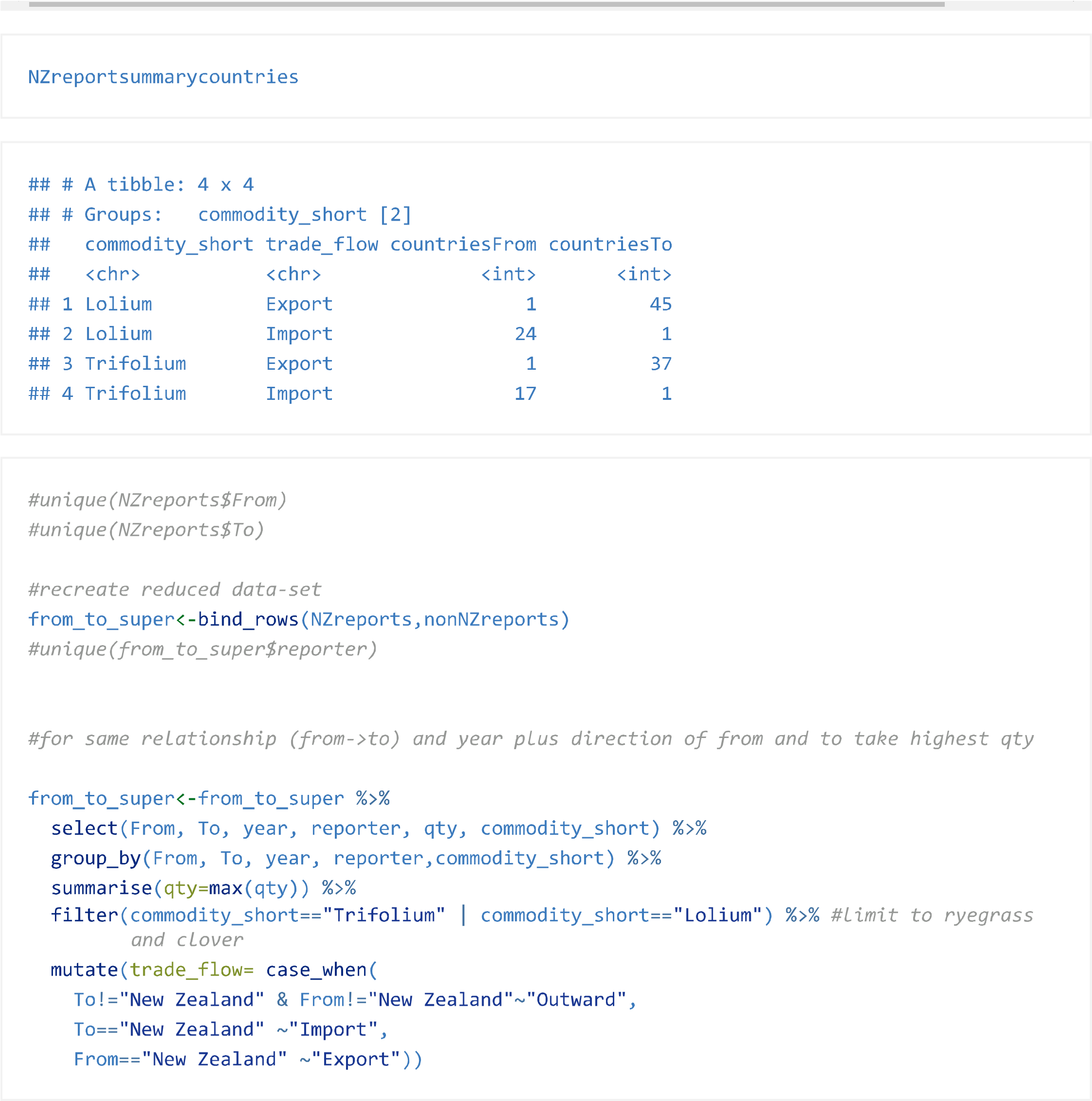


|  | | |
| --- | --- | --- |
|  | 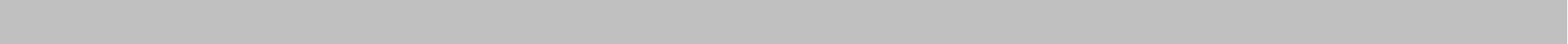 |  |


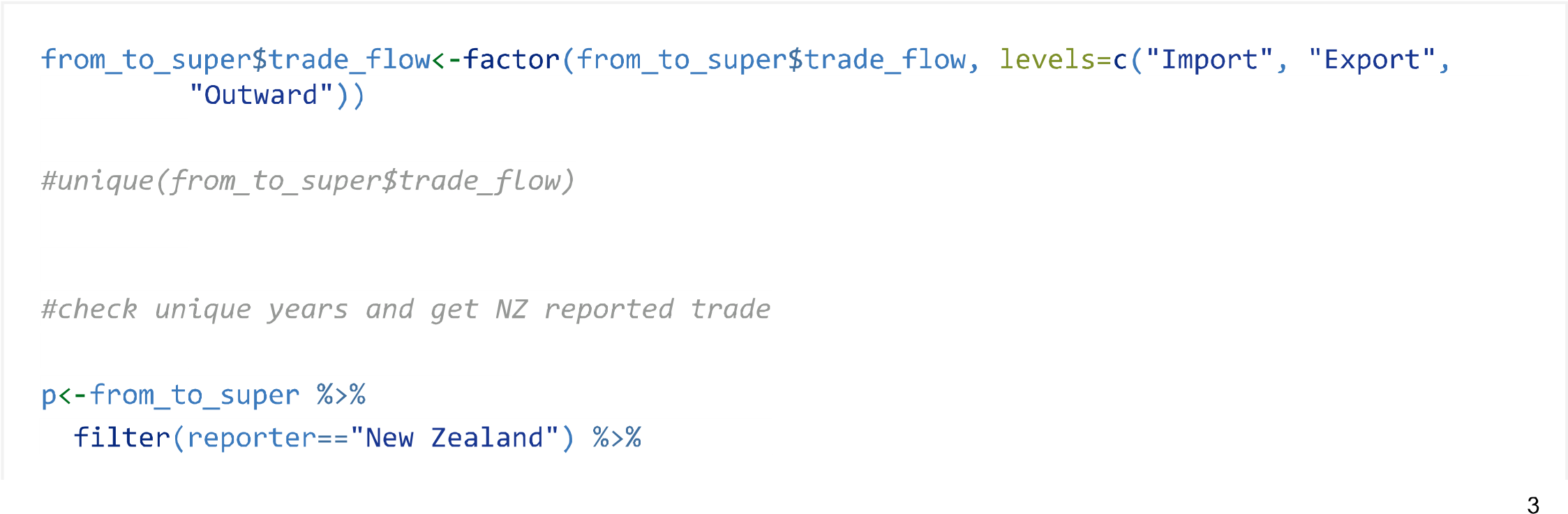


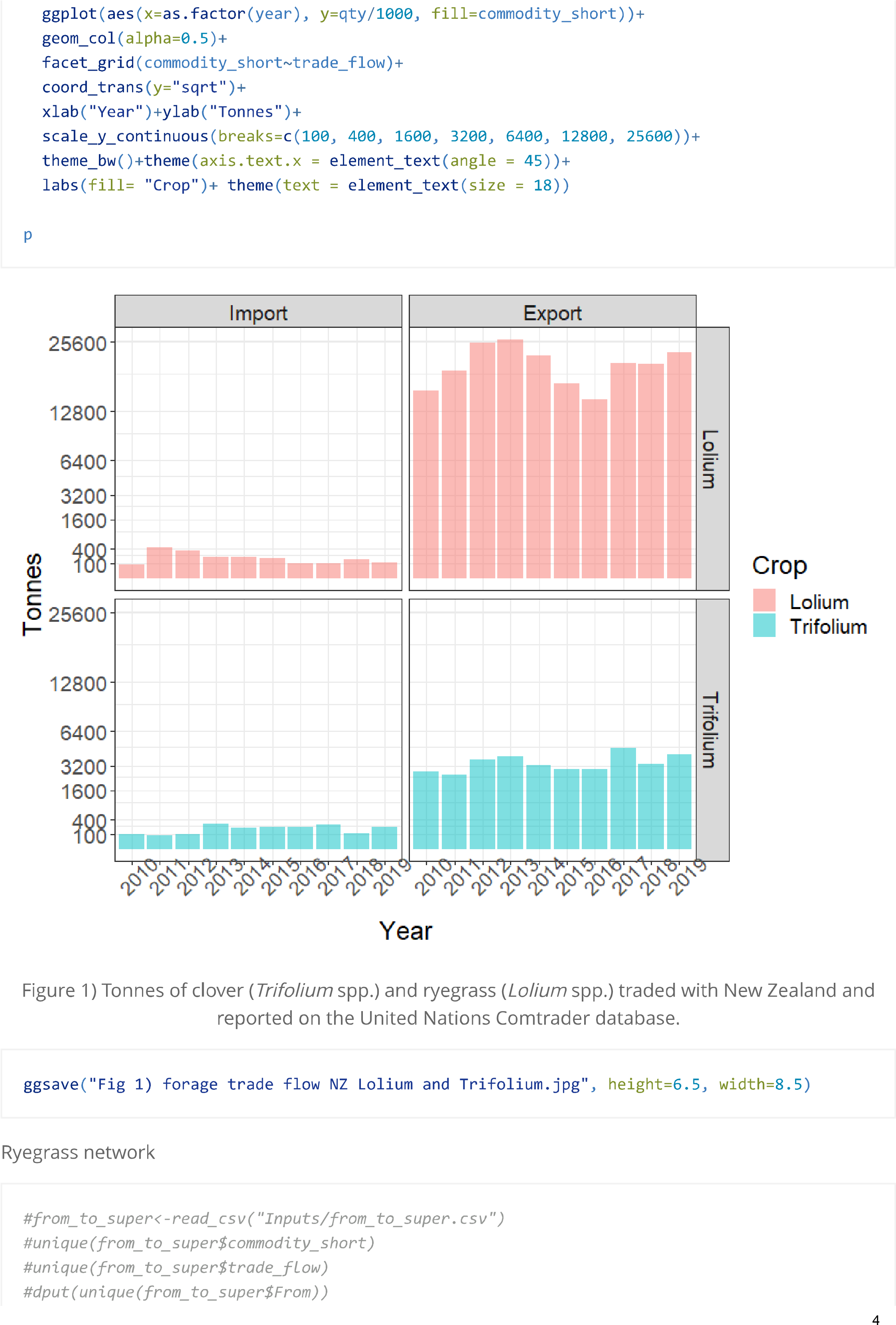


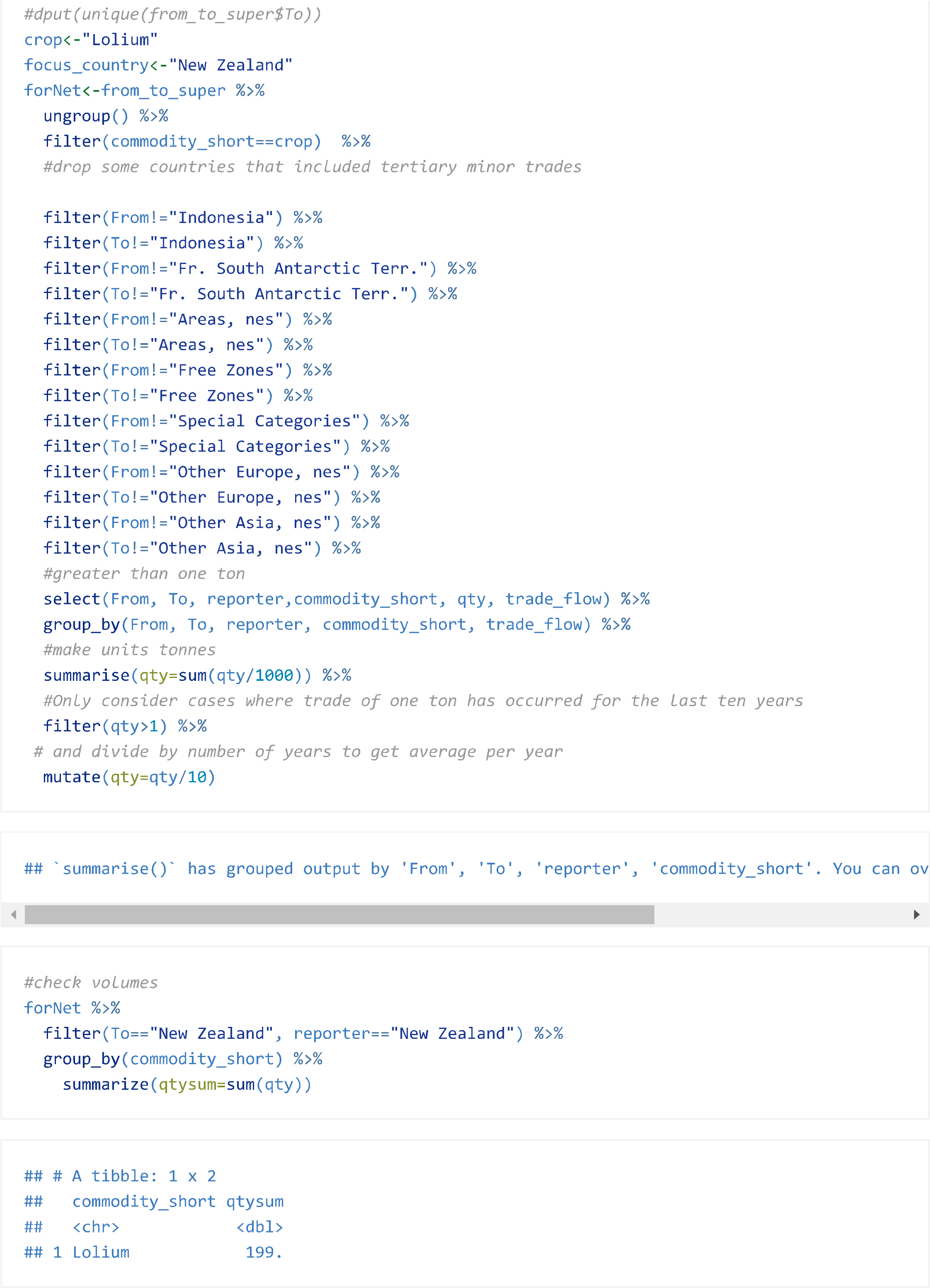


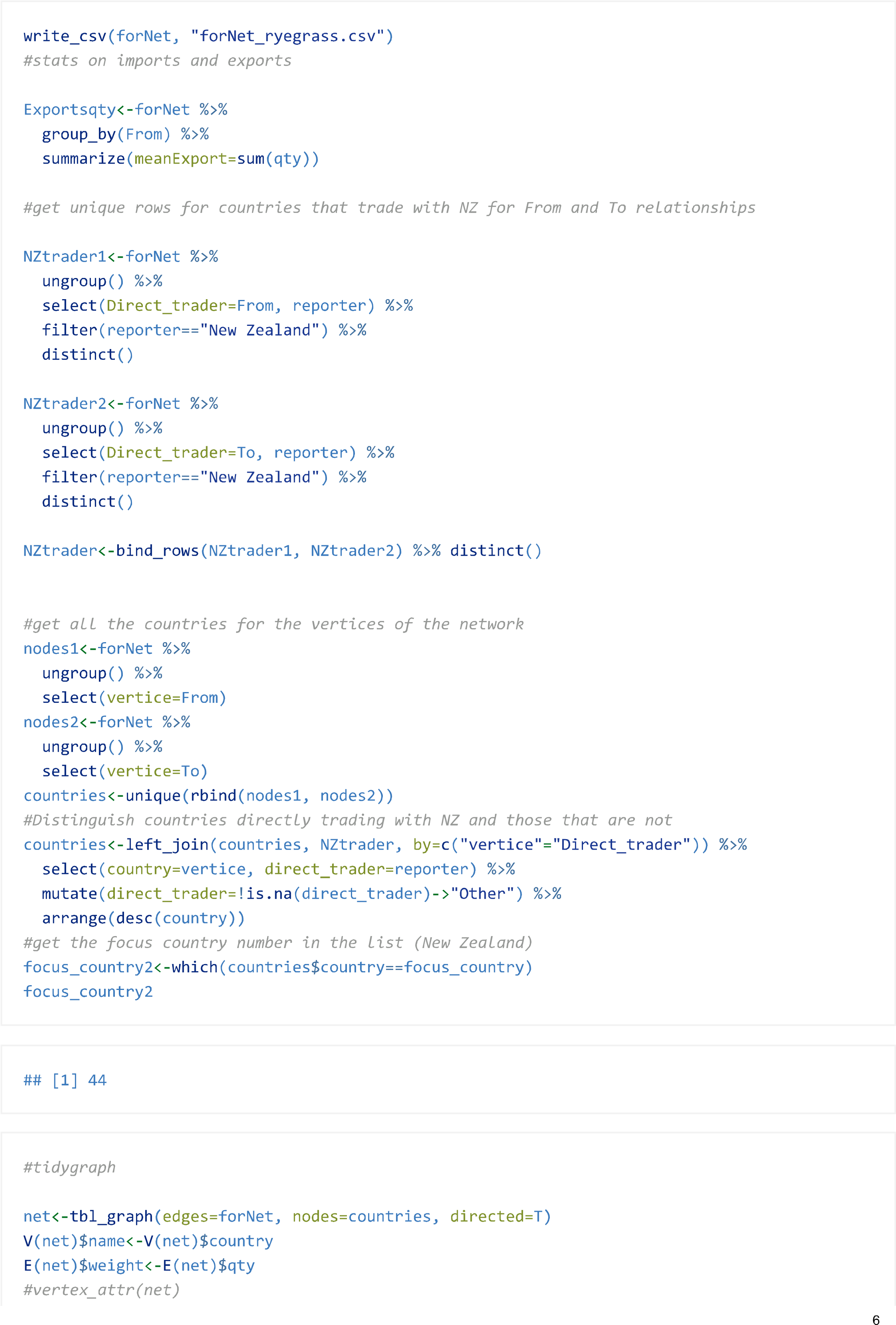


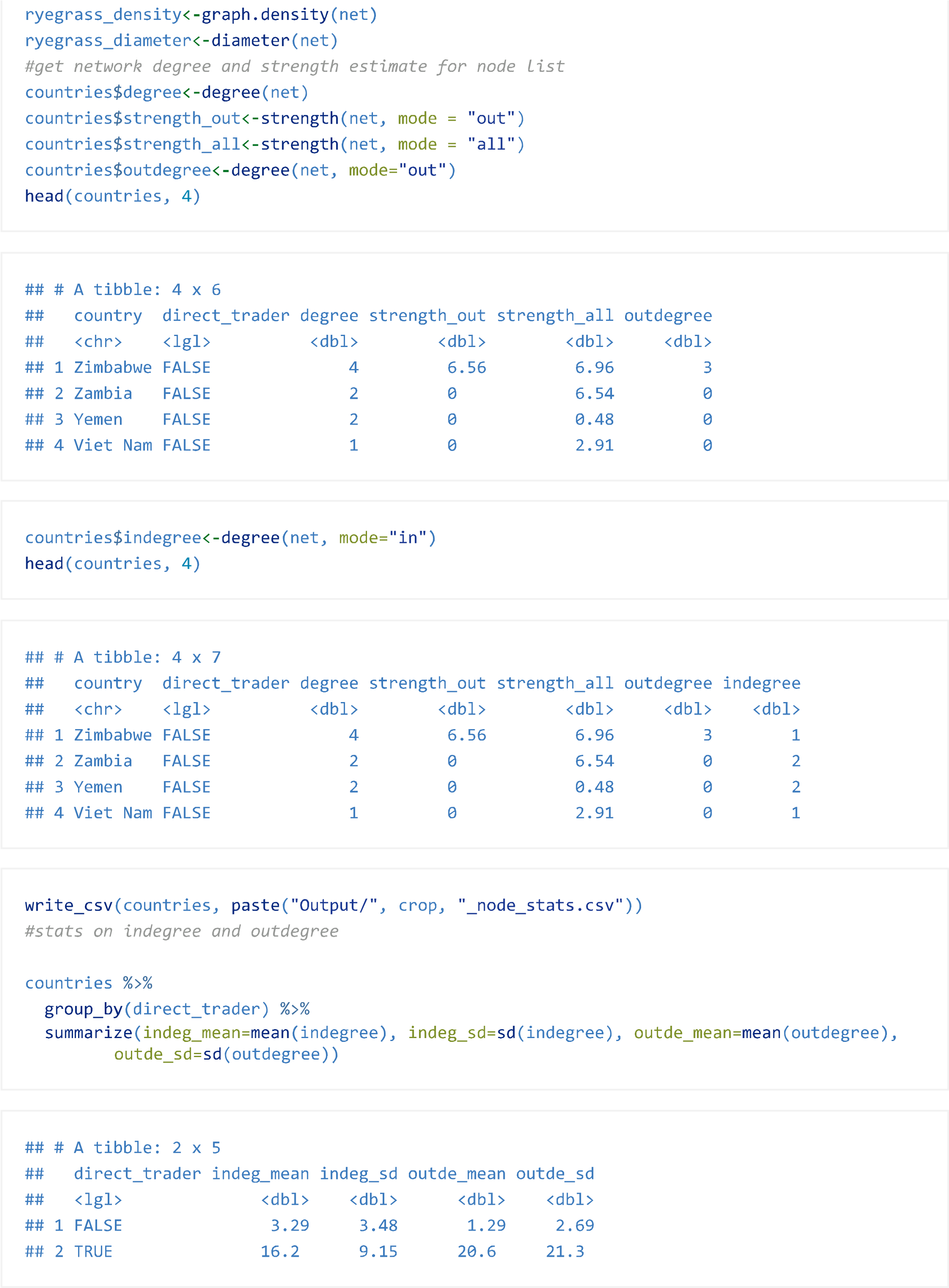


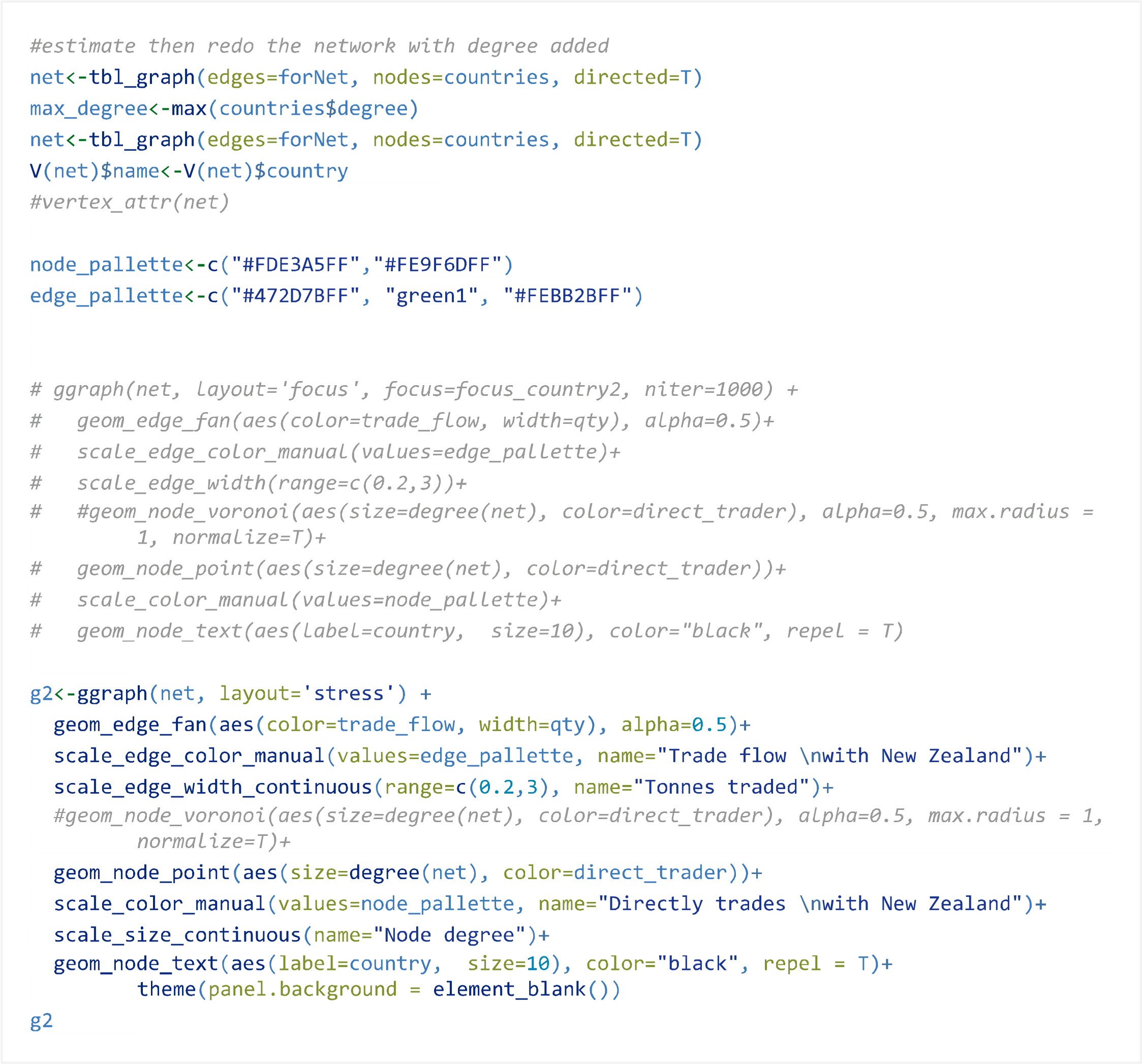


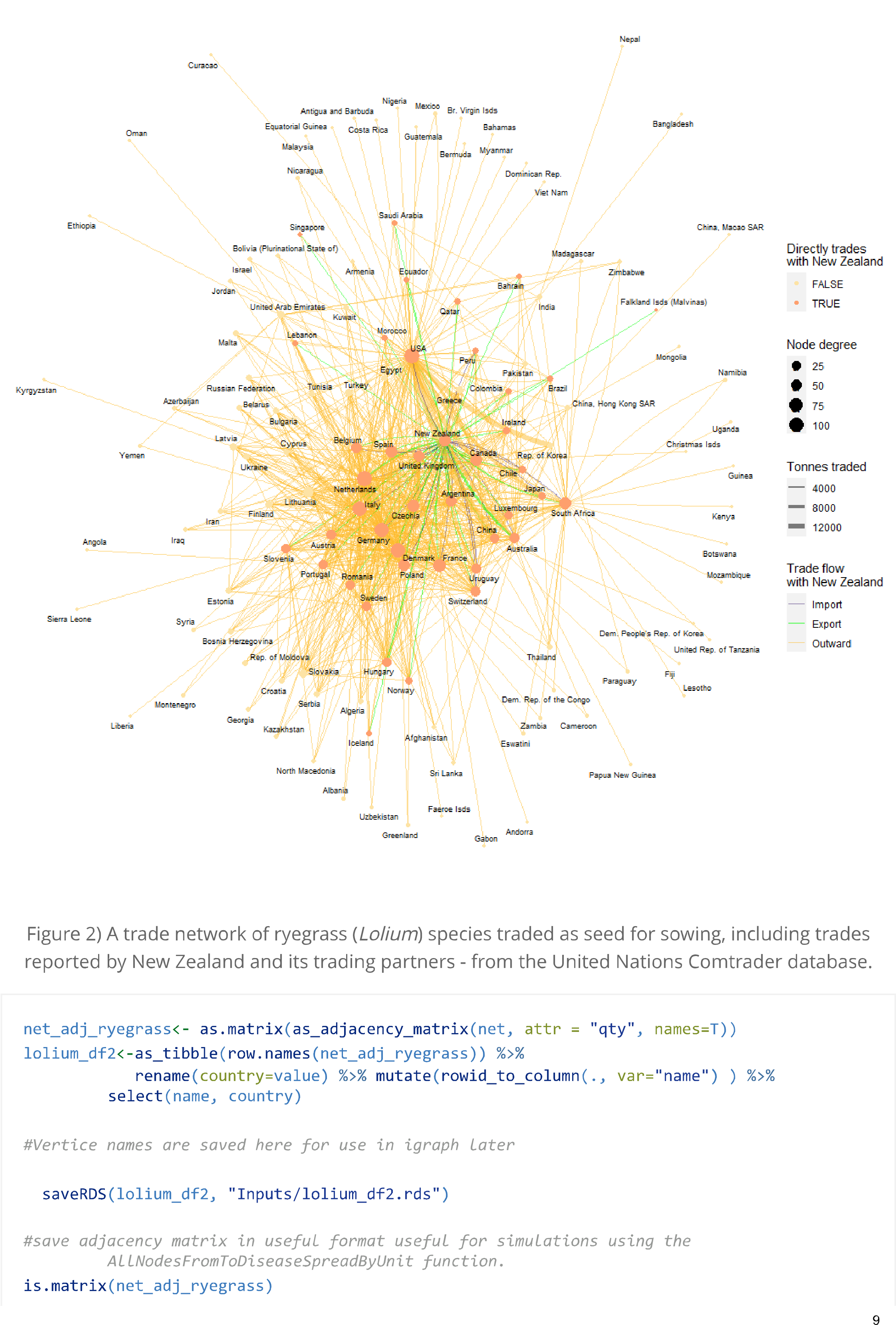


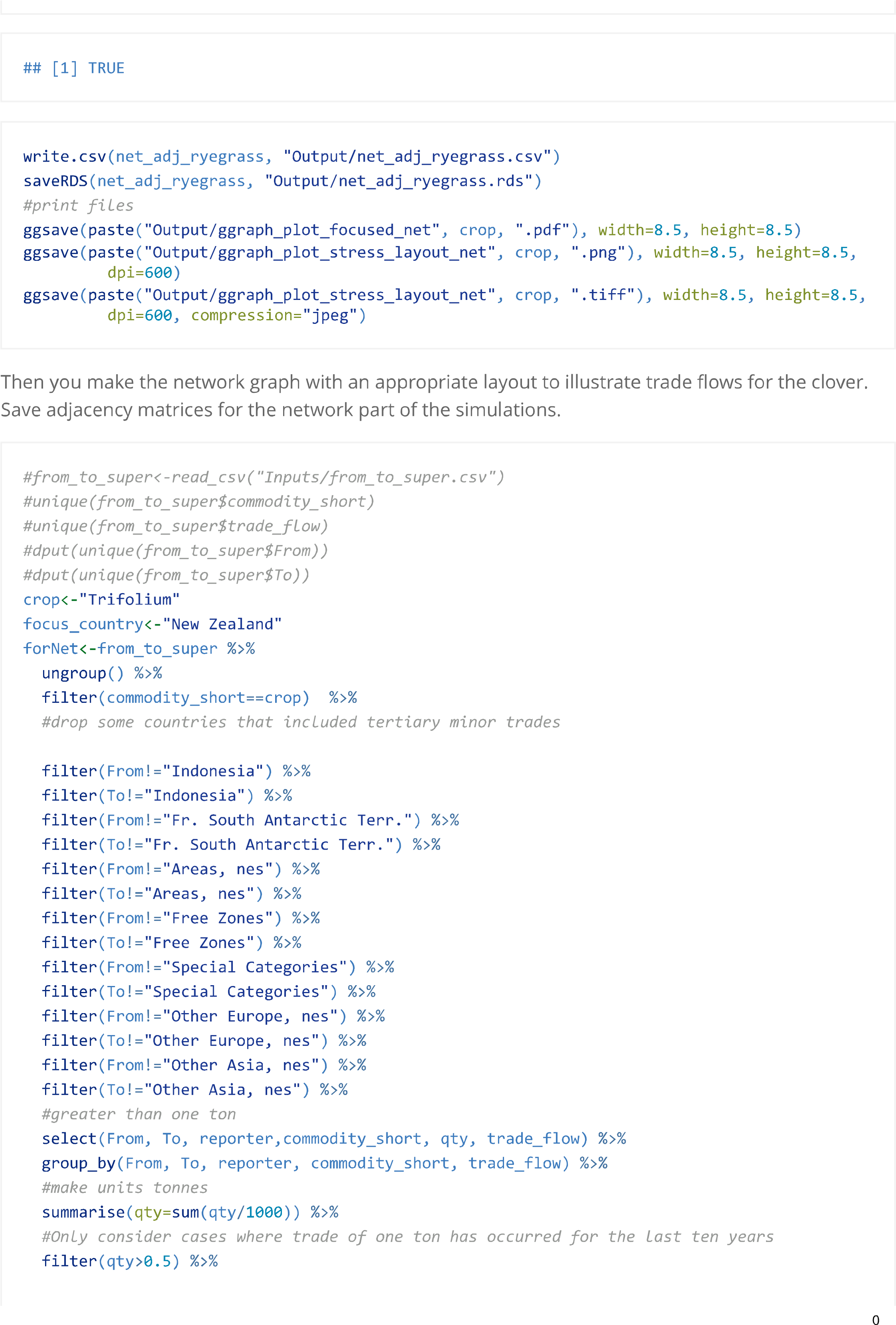


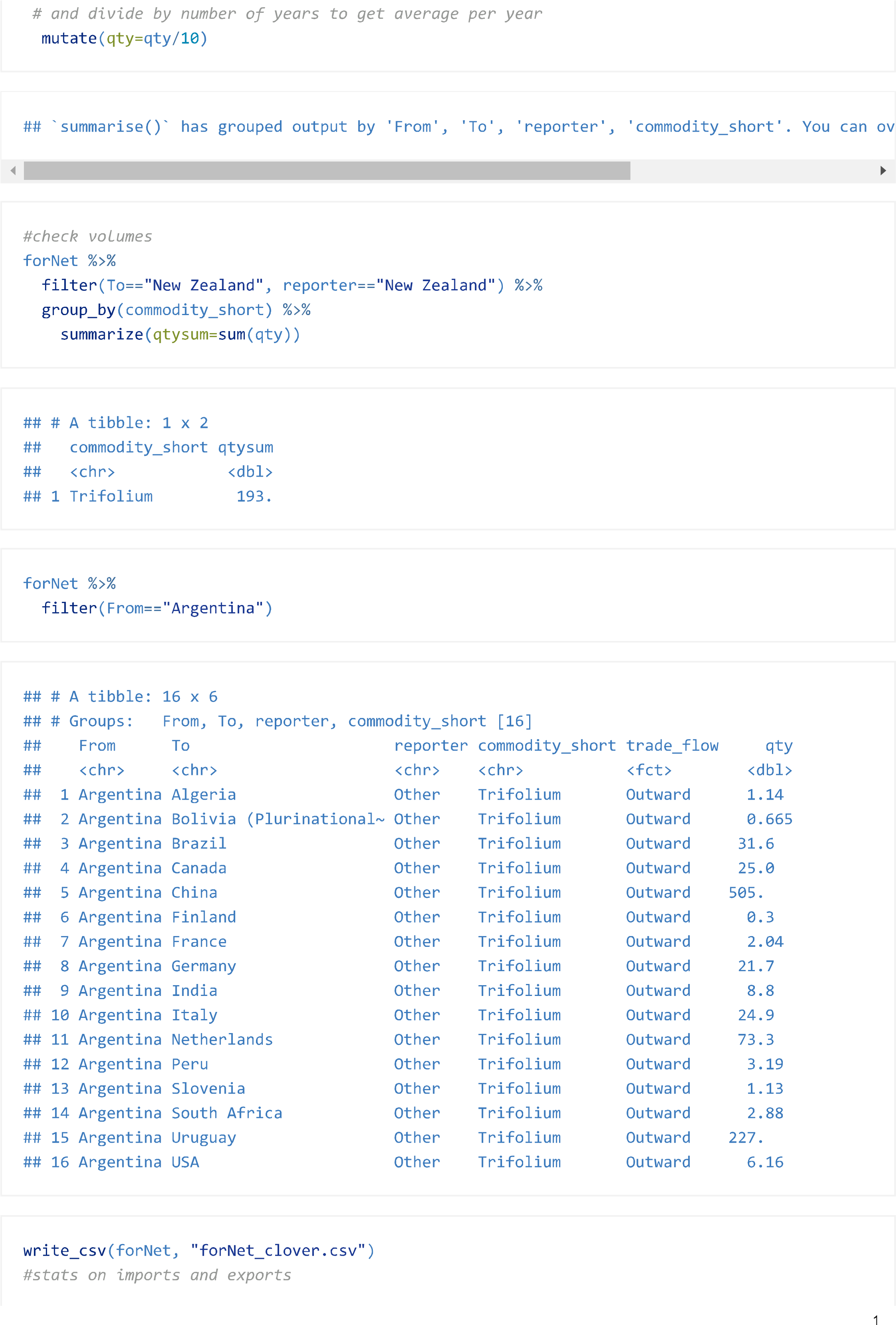


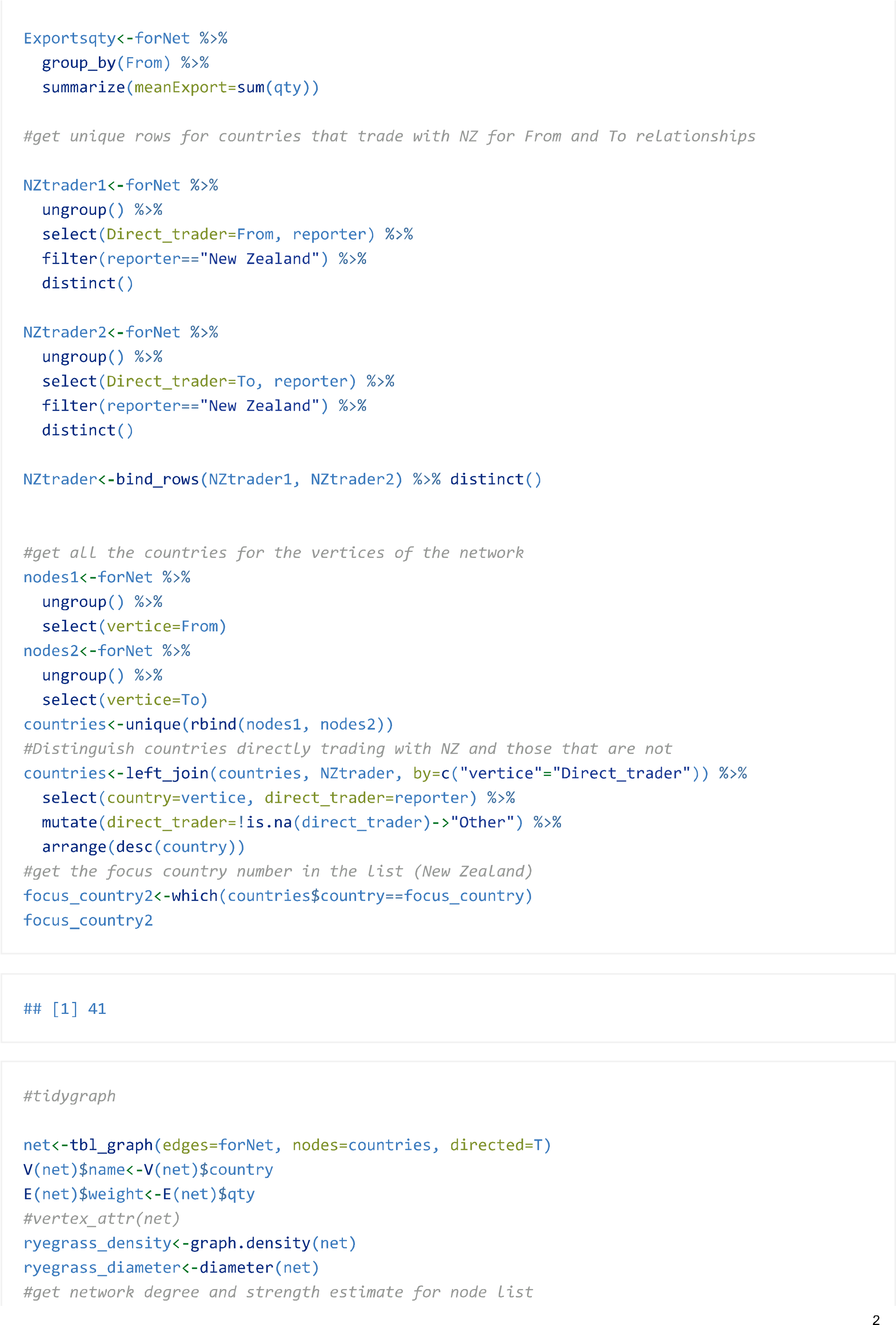


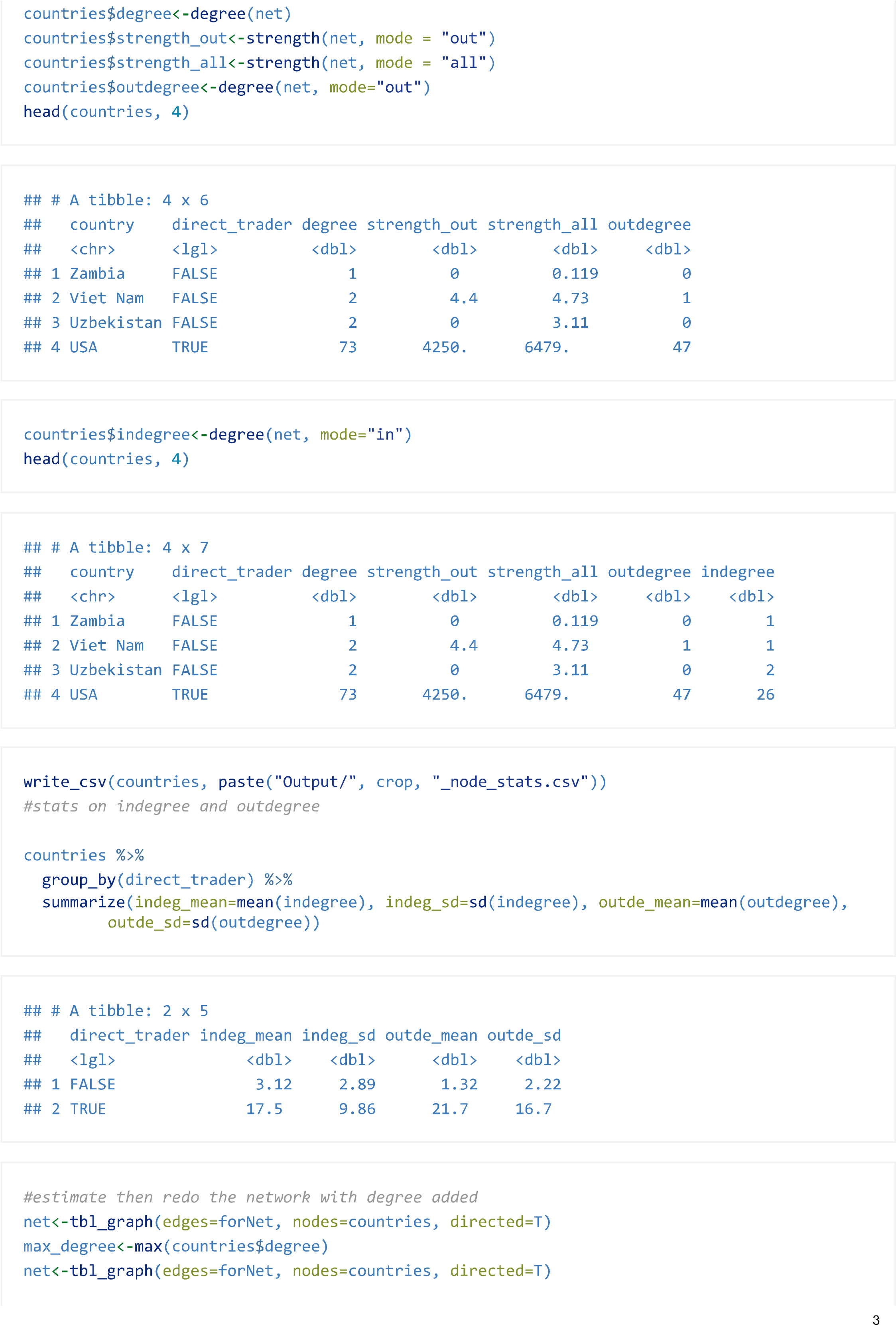


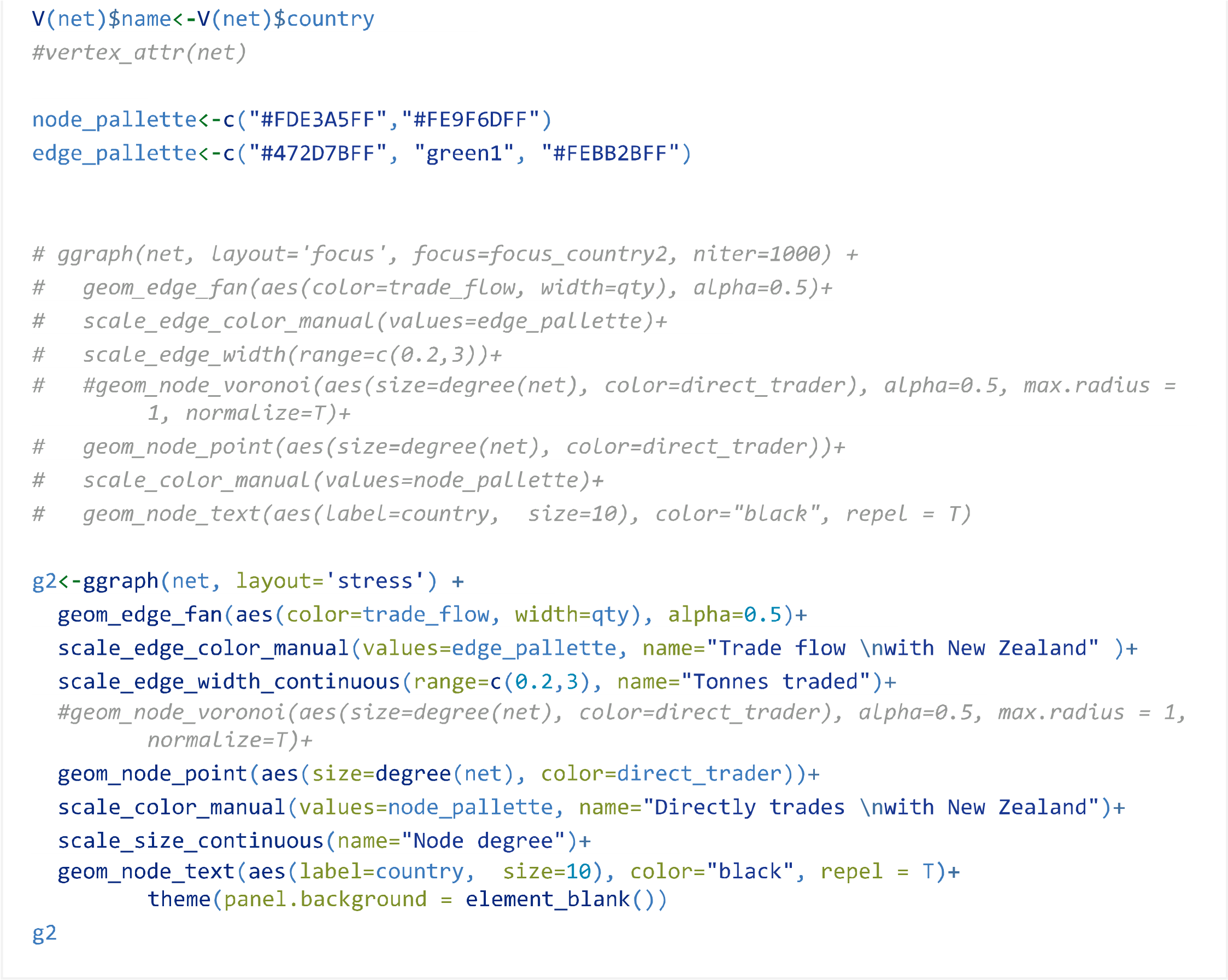


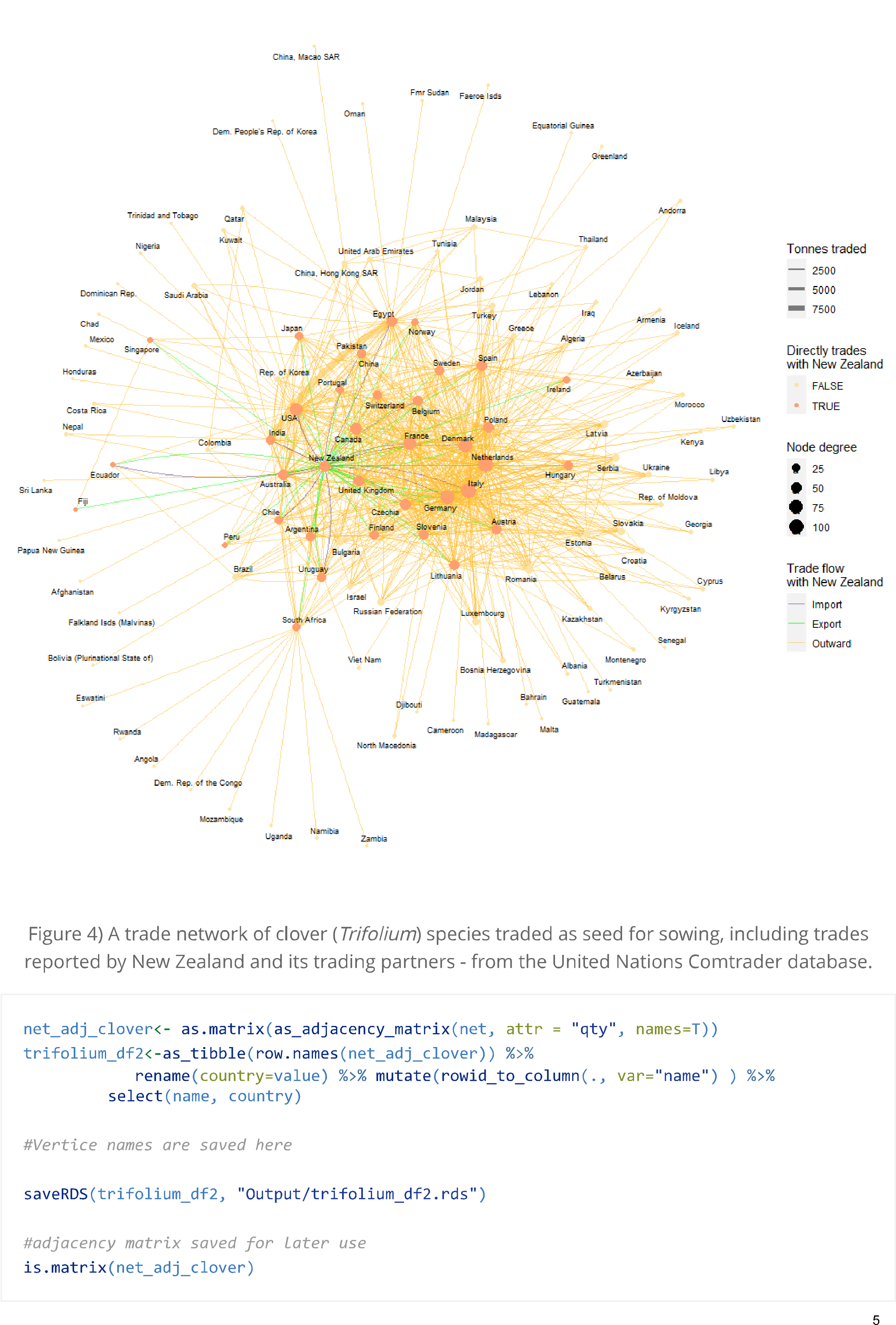


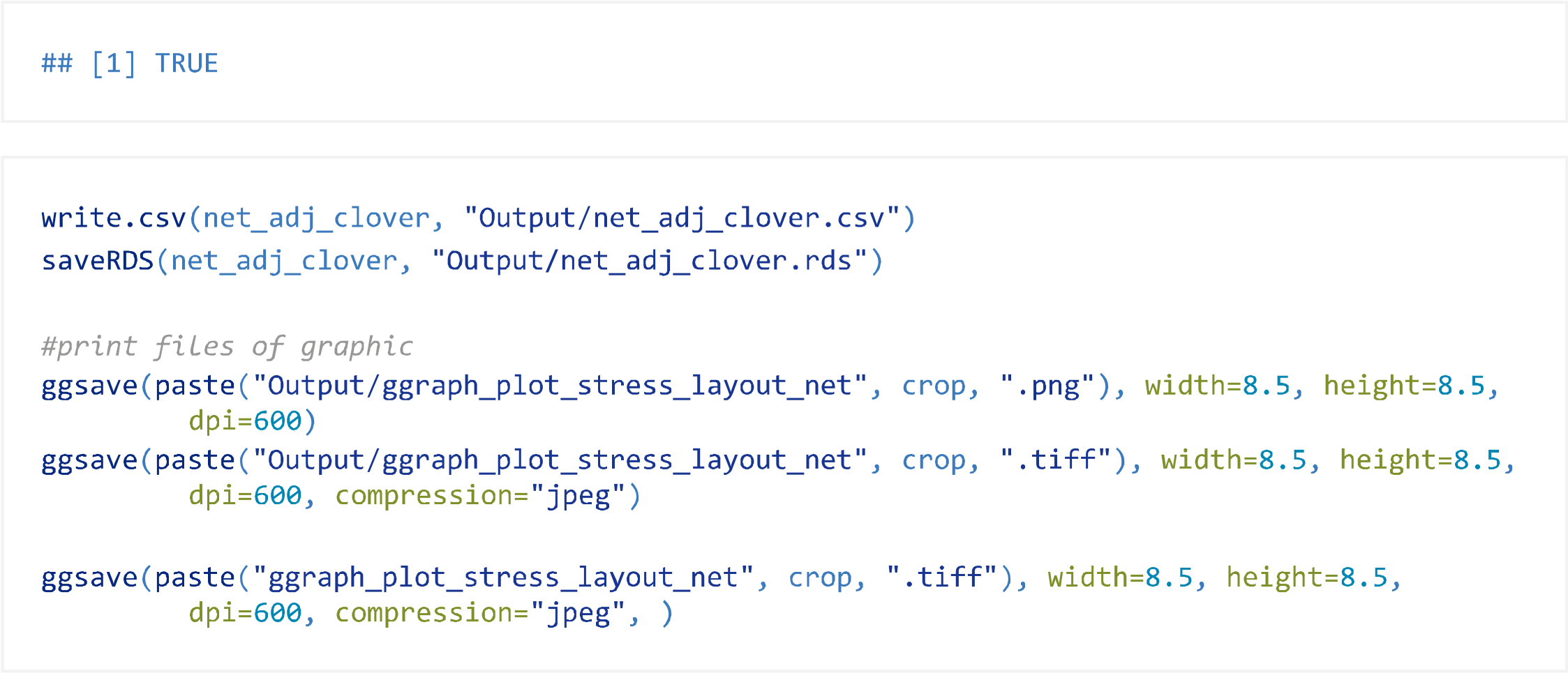


### Analyze NZ border inspection contaminant data for ryegrass and clover


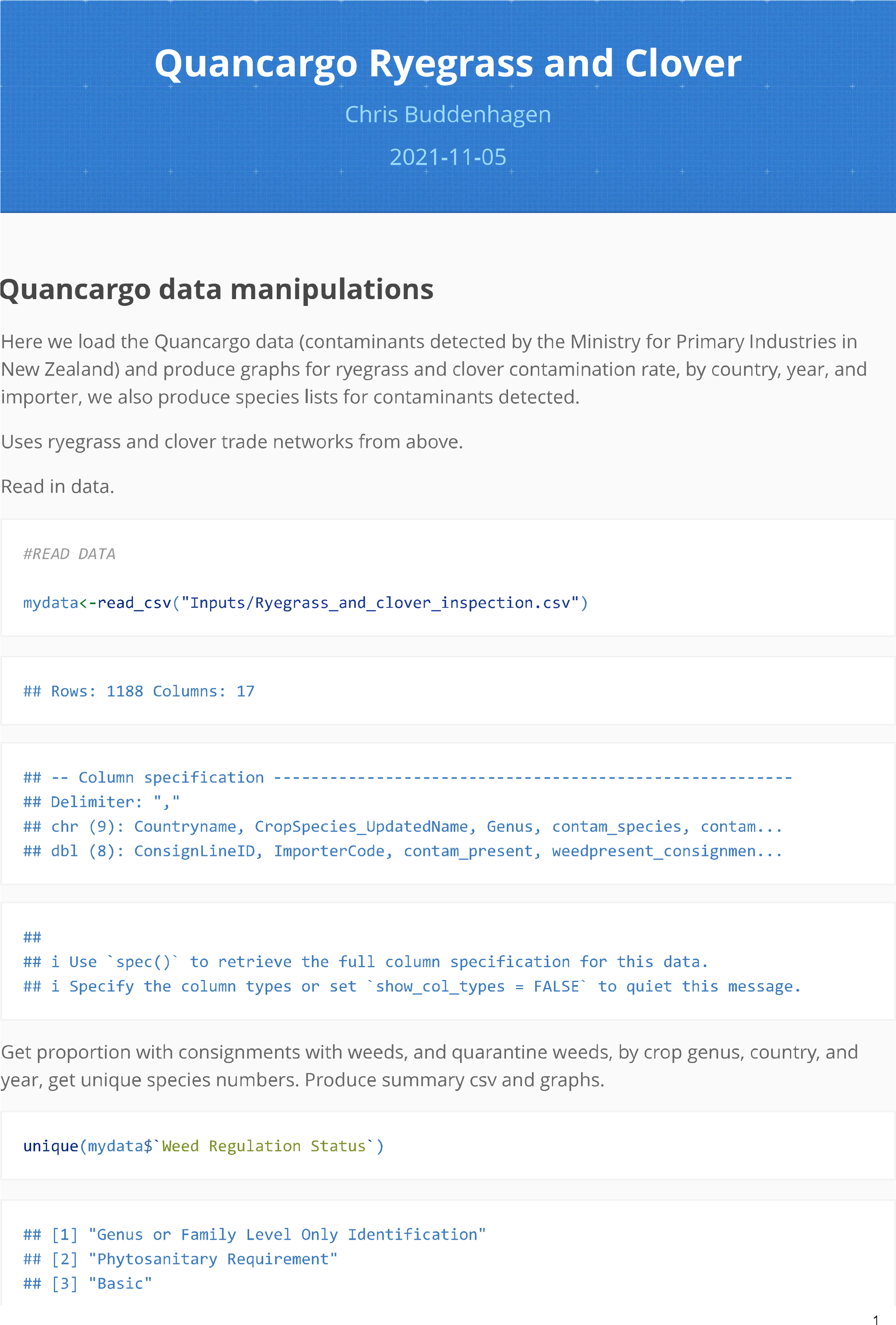


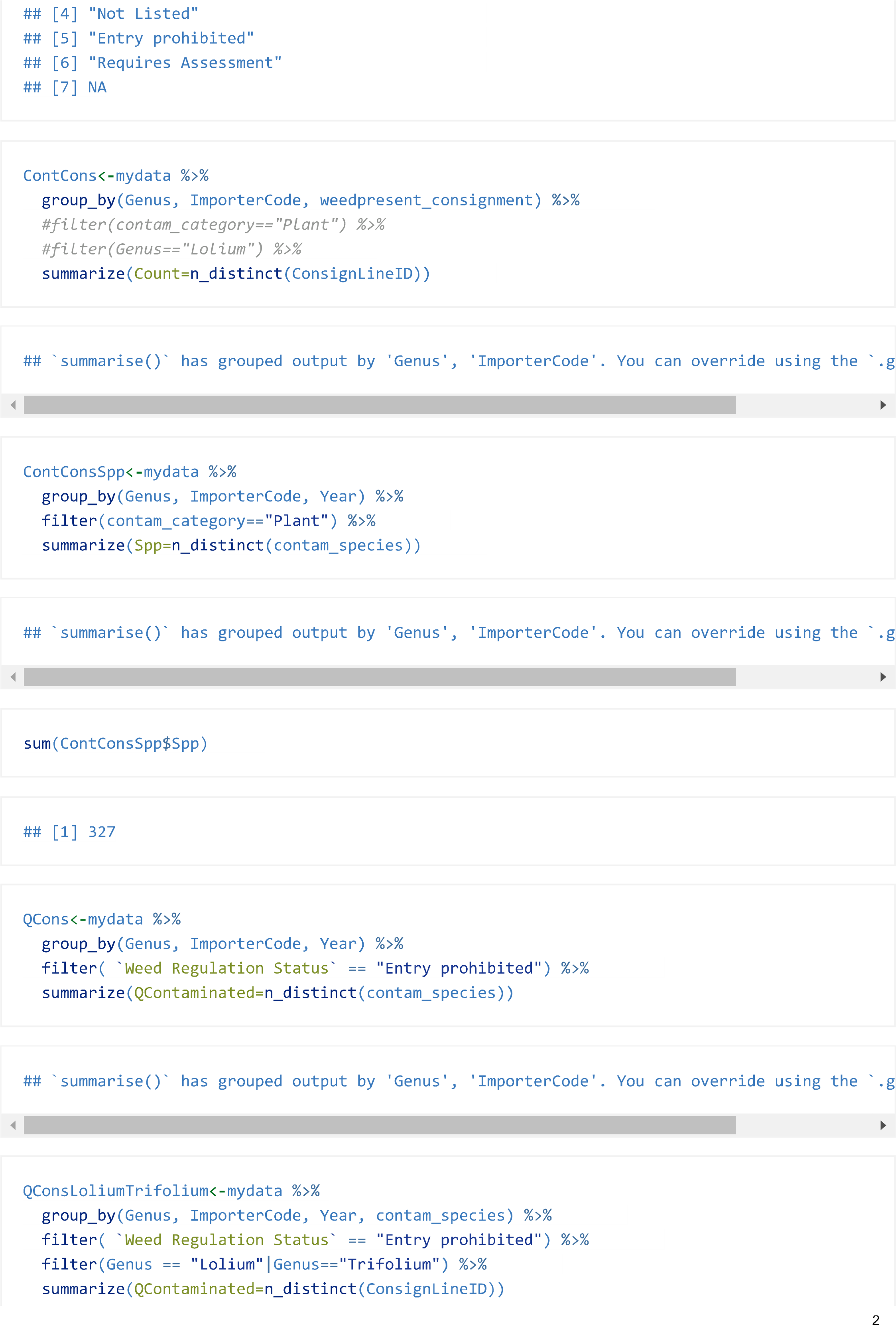


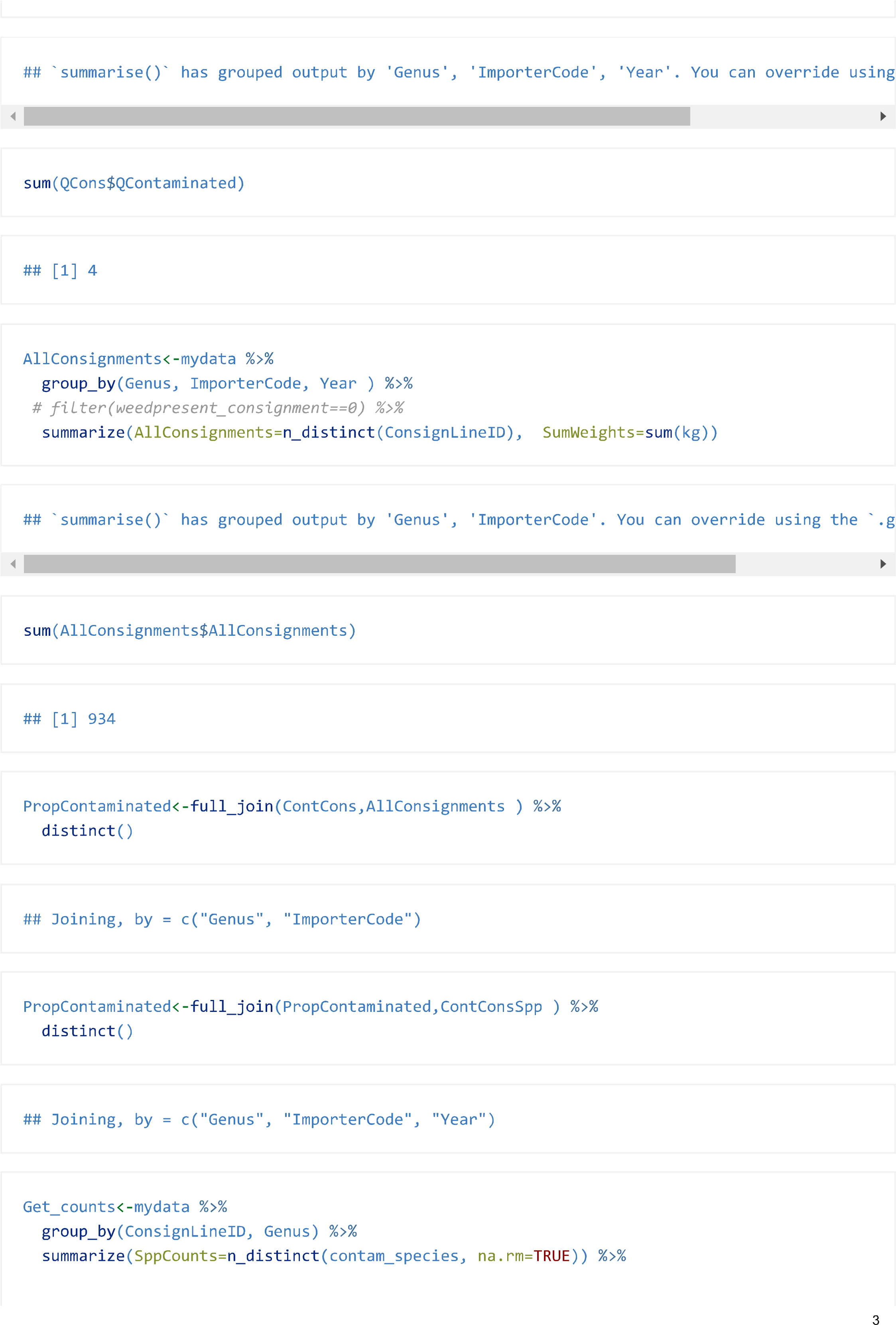


|  | | |
| --- | --- | --- |
|  | 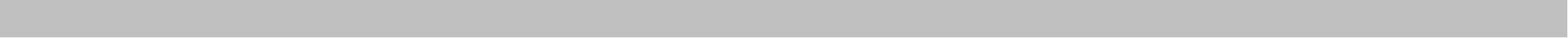 |  |


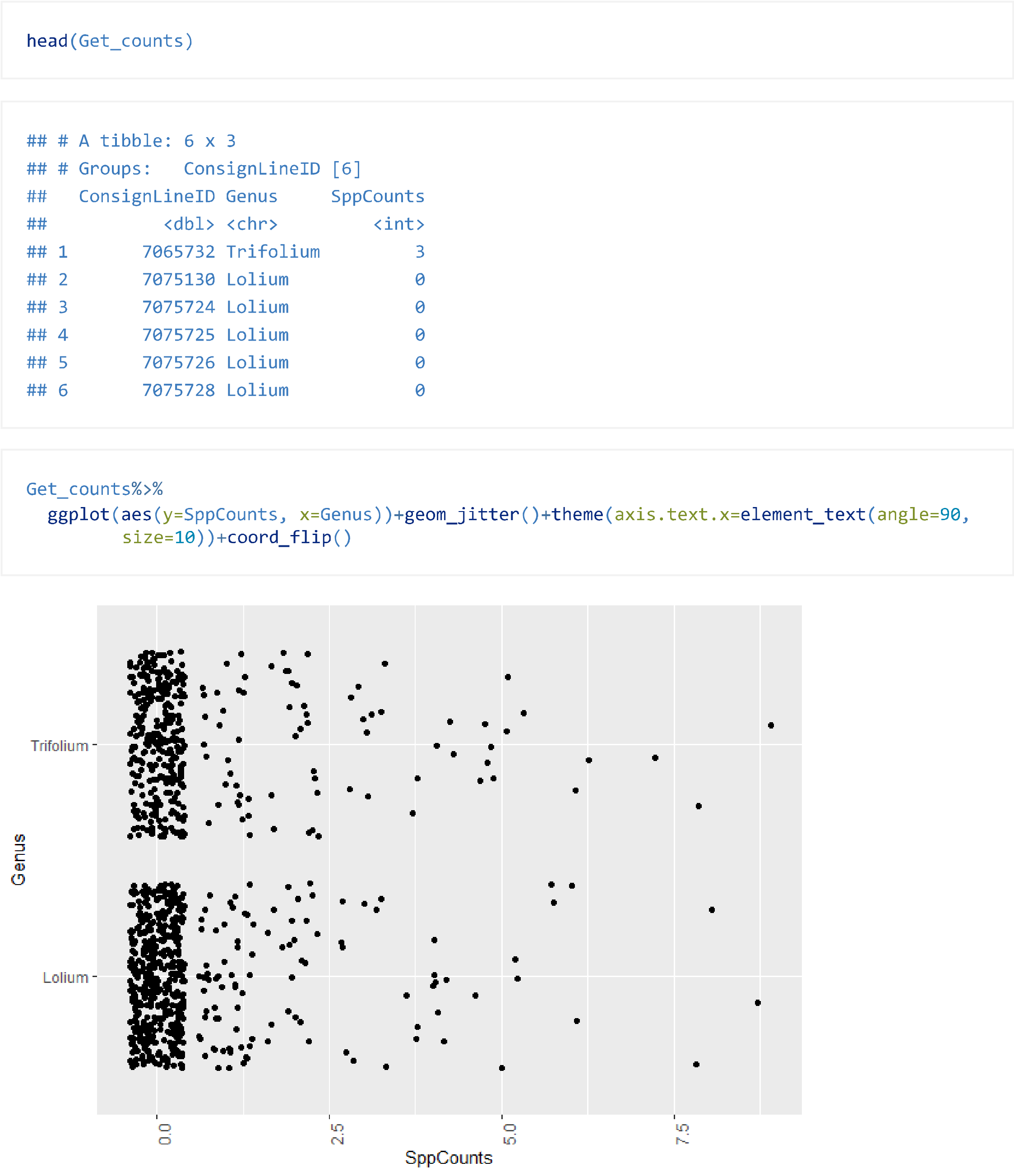


| \|  \|  \|  \|  \| \| --- \| --- \| --- \| --- \| \| |
| --- | --- | --- | --- | --- |
|  |


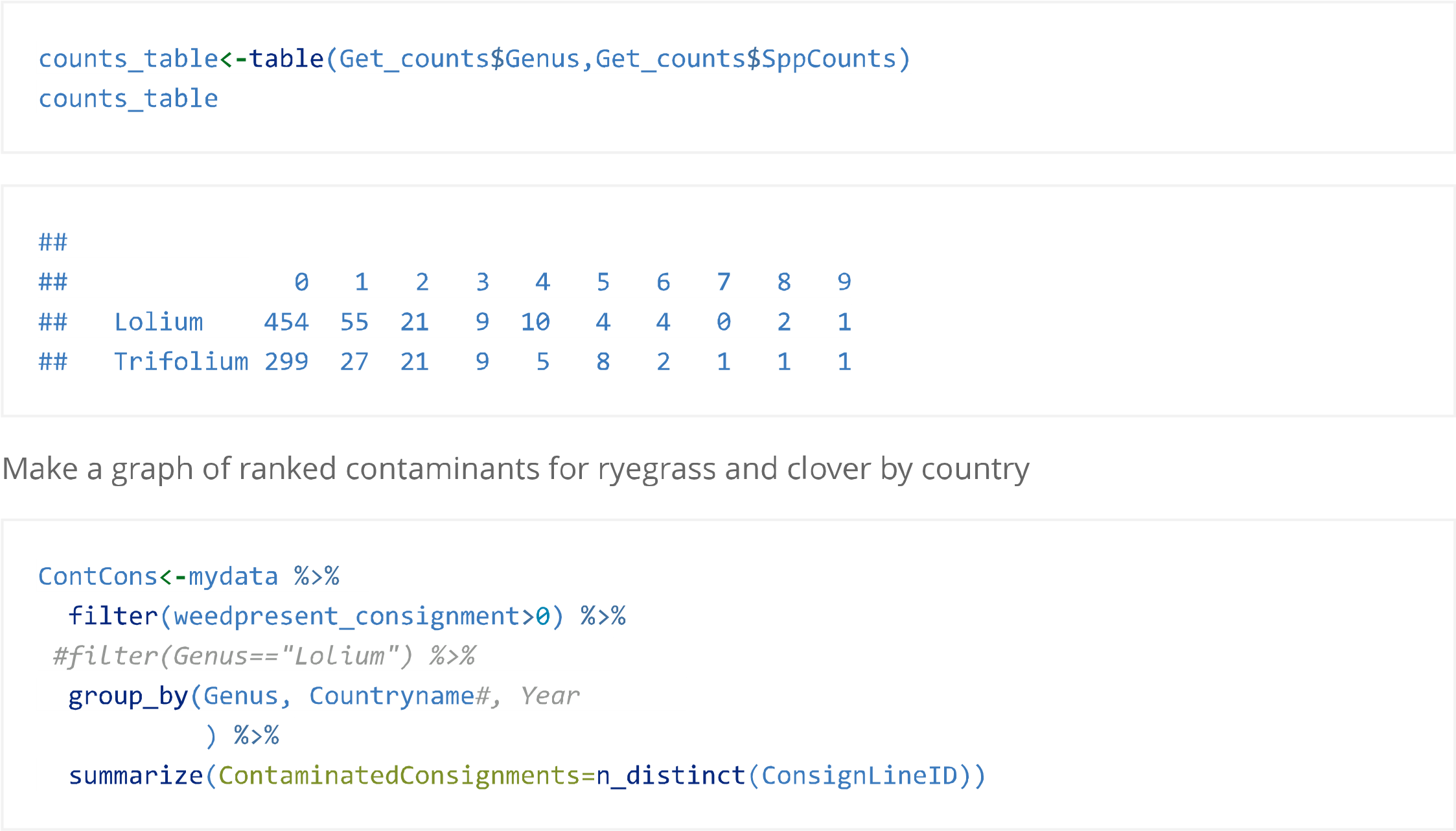


|  | | |
| --- | --- | --- |
|  | 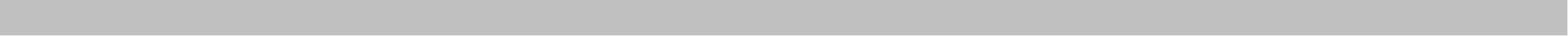 |  |


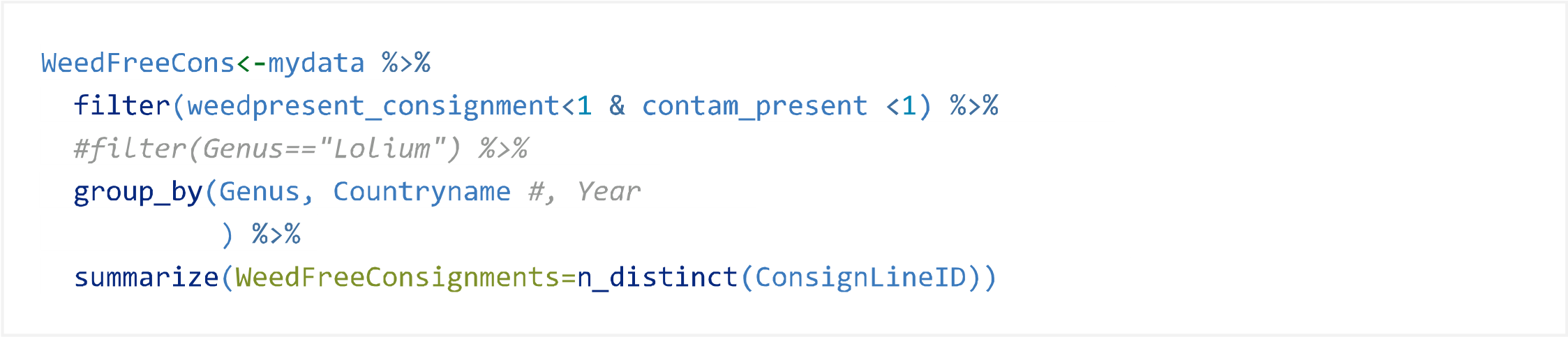


|  | | |
| --- | --- | --- |
|  | 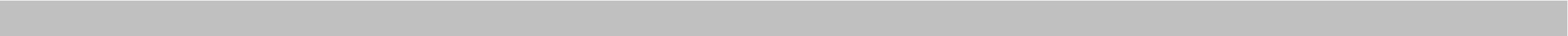 |  |


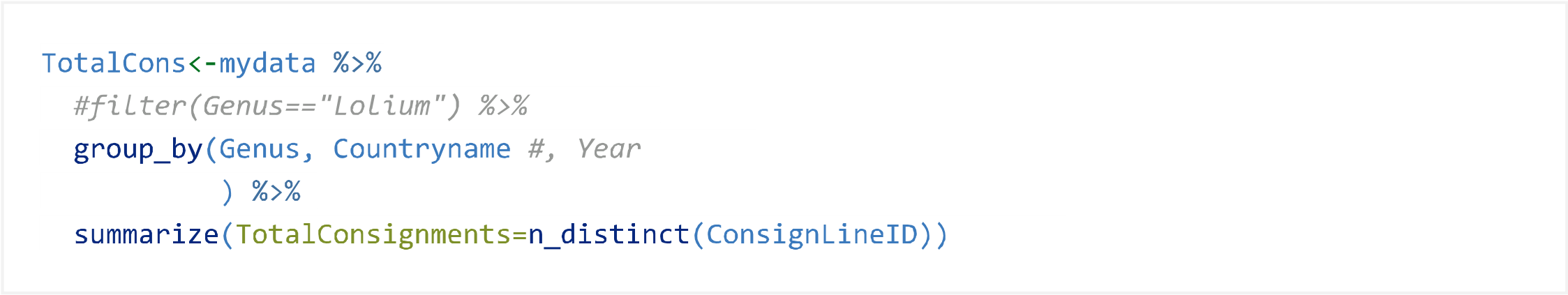


|  | | |
| --- | --- | --- |
|  | 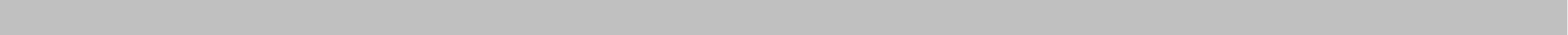 |  |


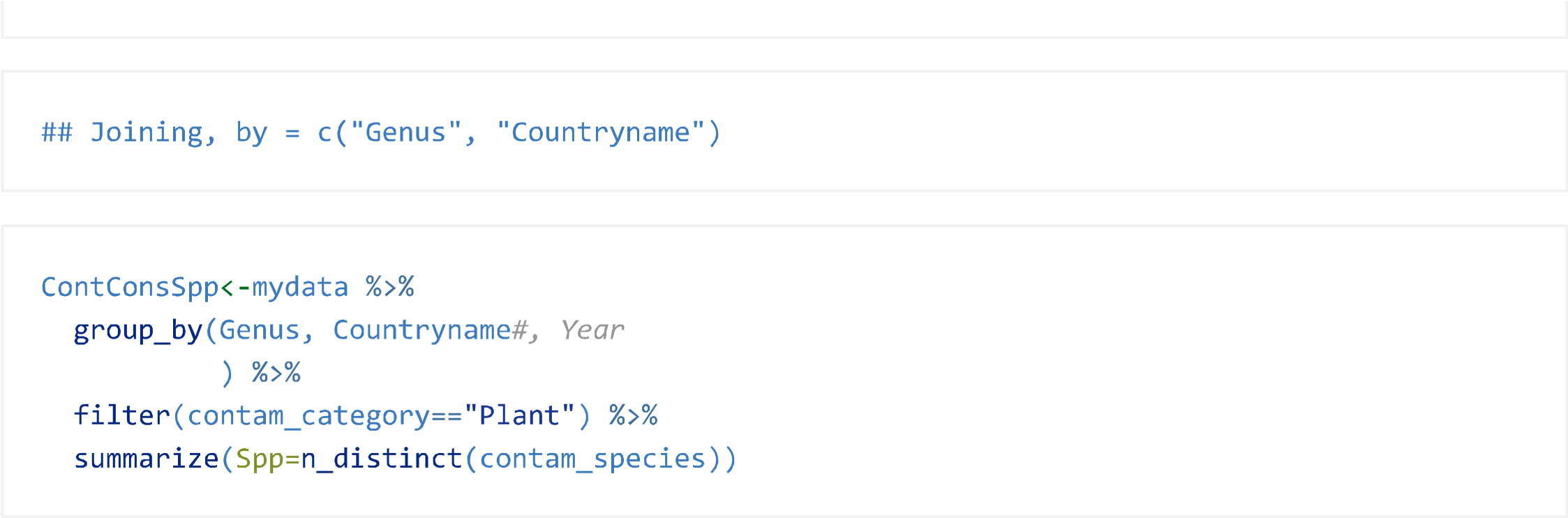


|  | | |
| --- | --- | --- |
|  | 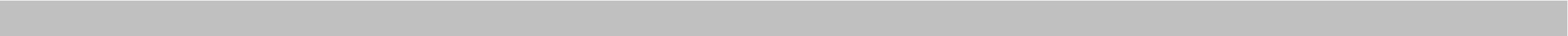 |  |


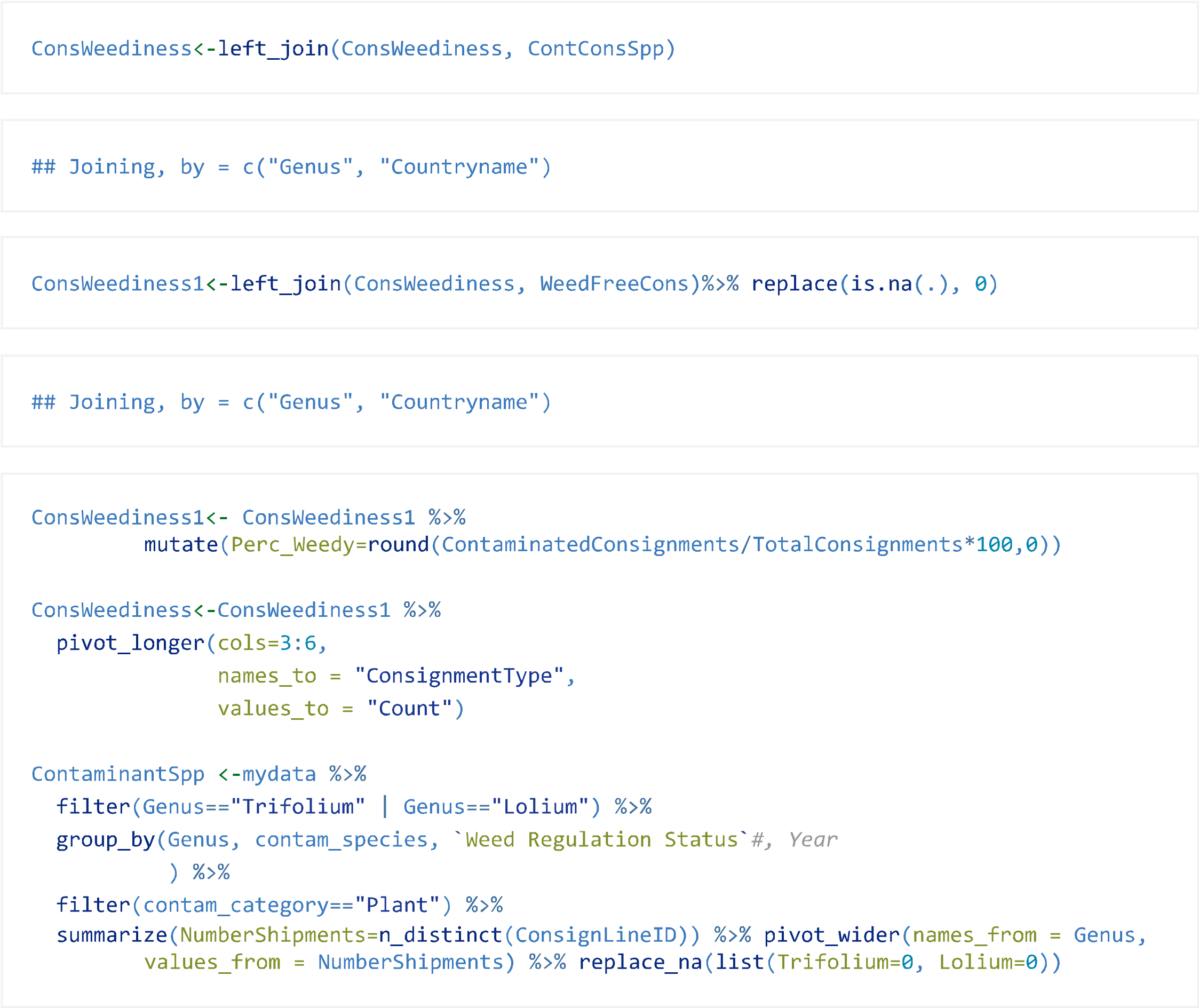


|  | | |
| --- | --- | --- |
|  | 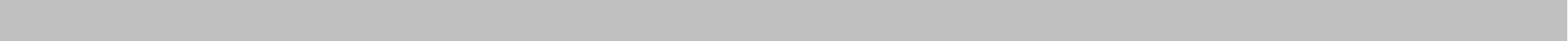 |  |


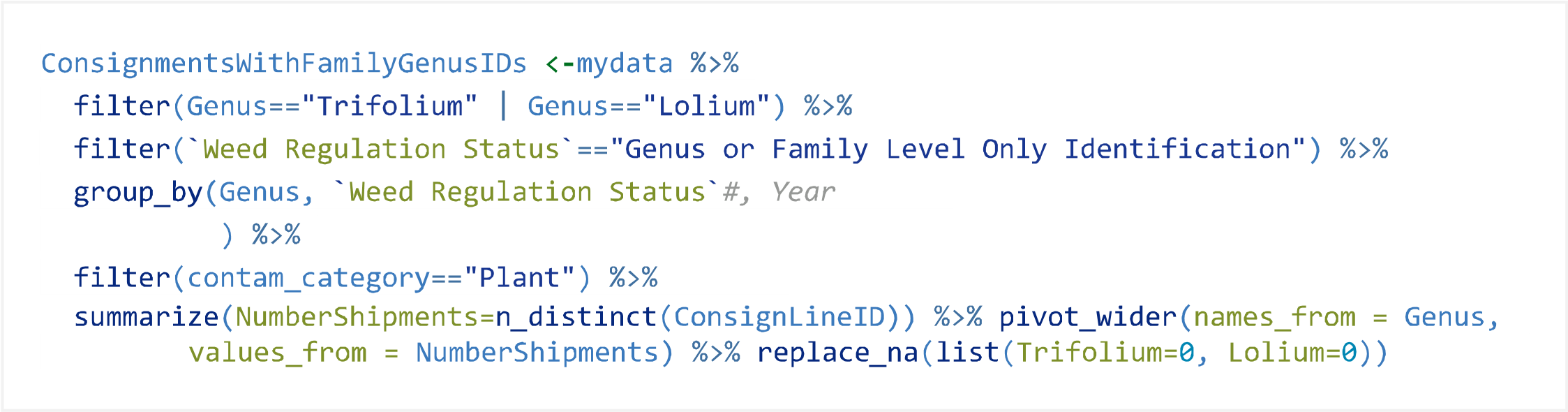


|  | | |
| --- | --- | --- |
|  | 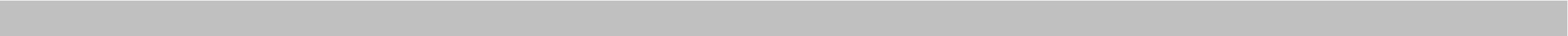 |  |


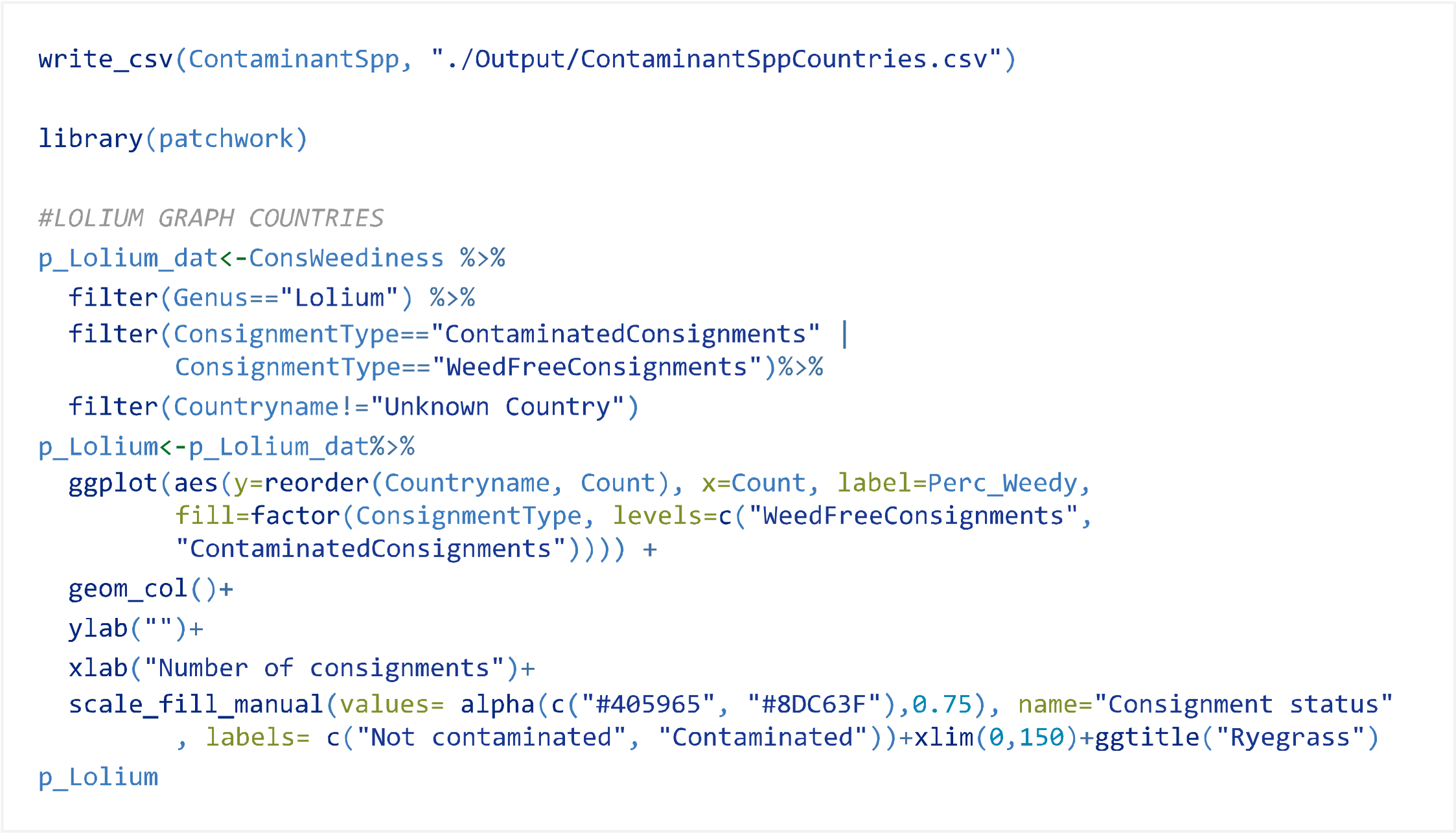


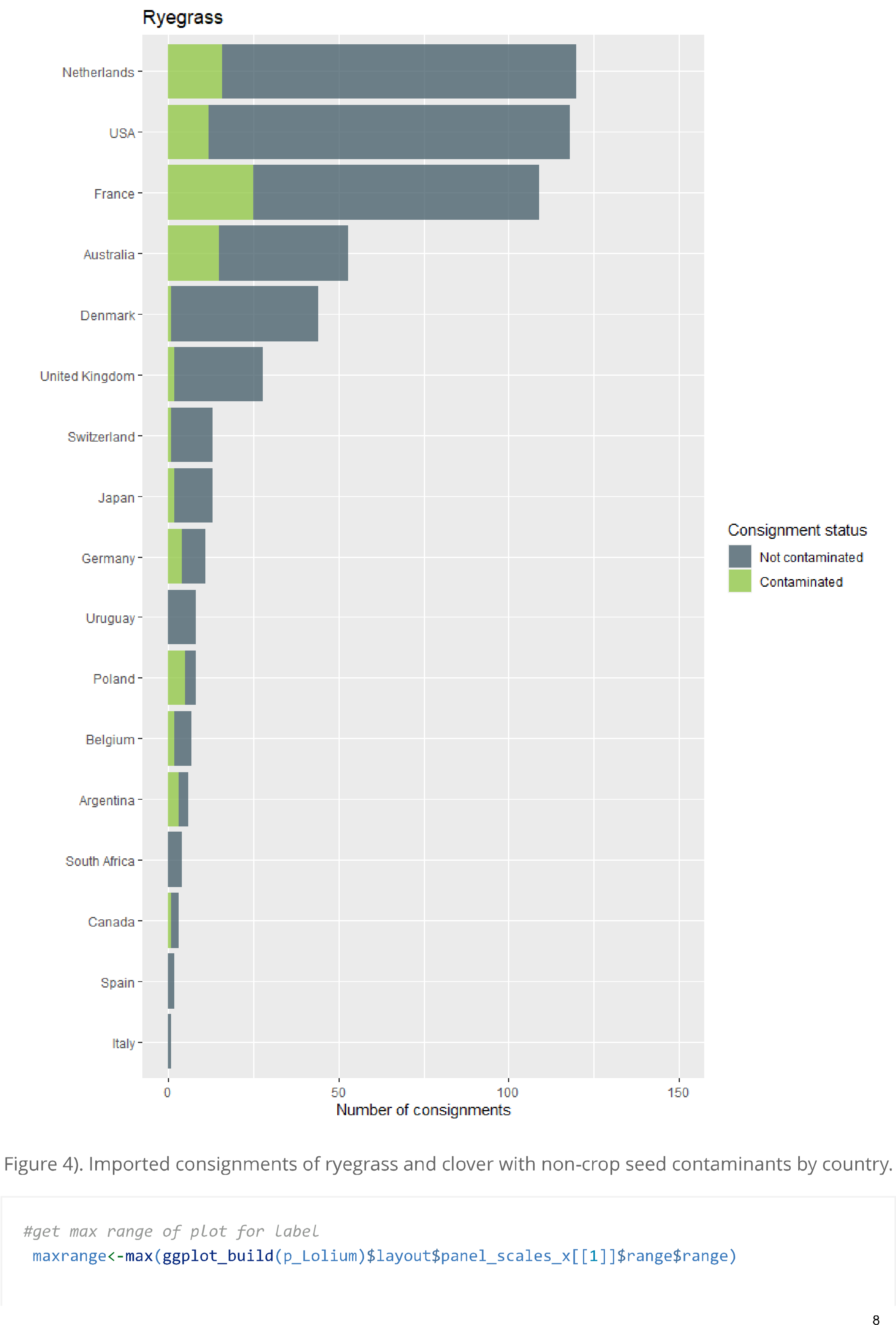


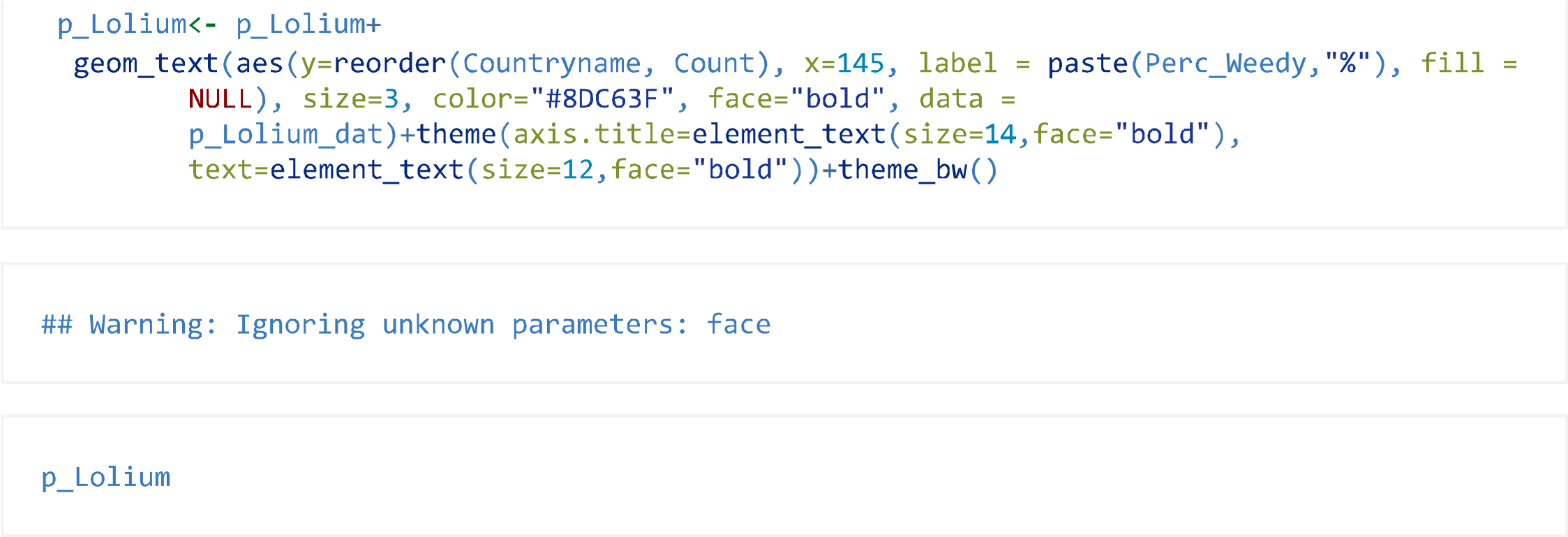


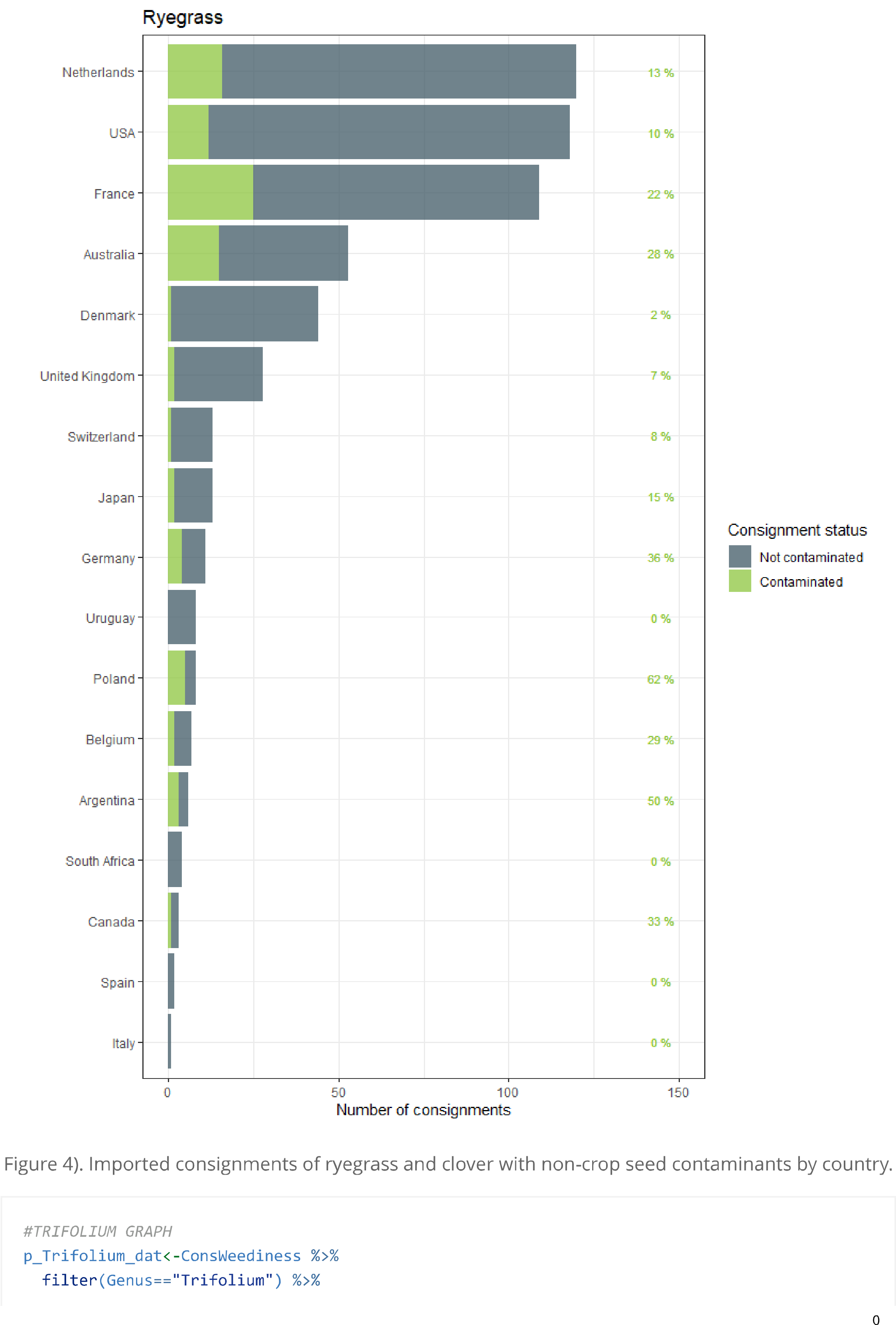


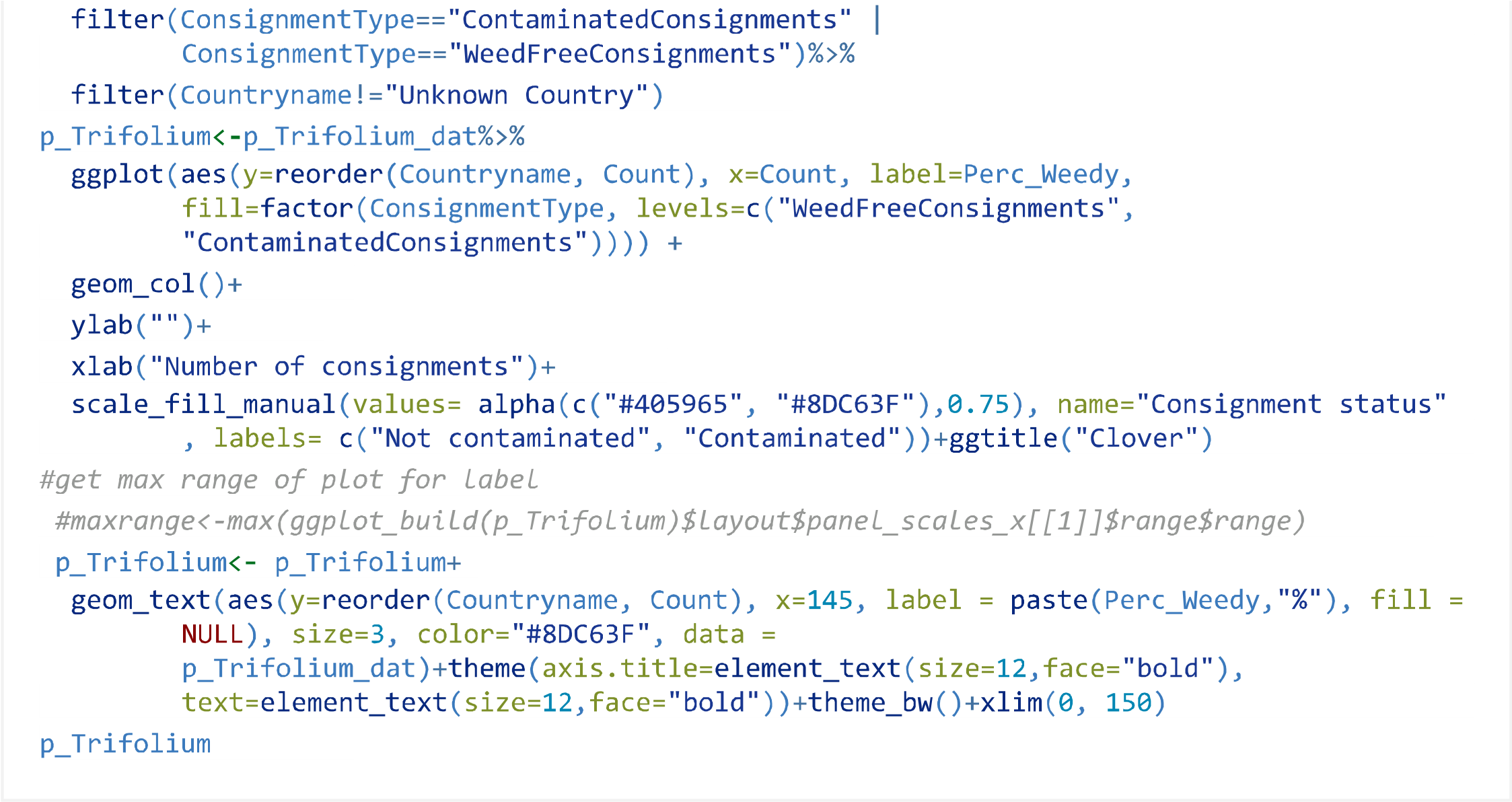


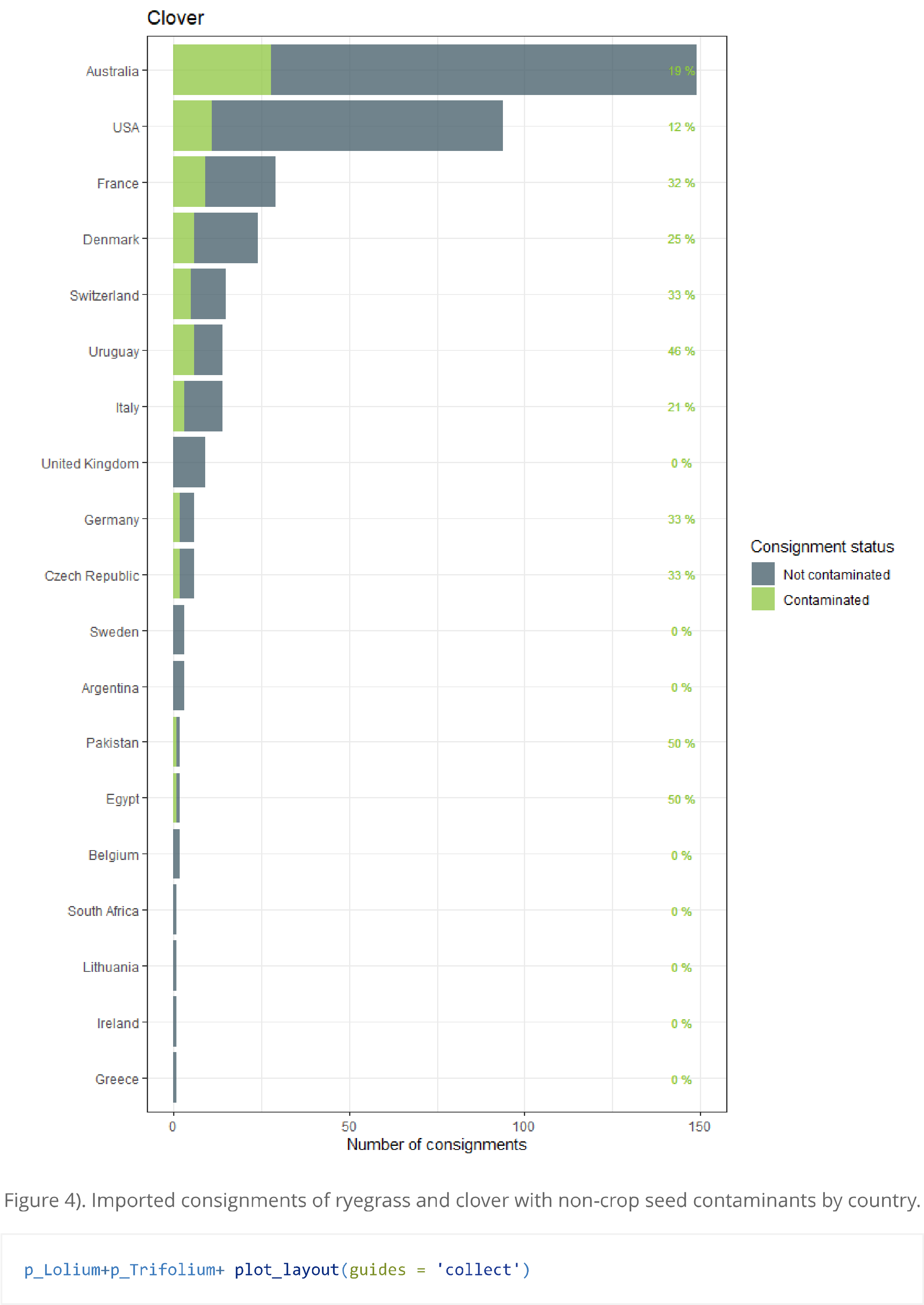


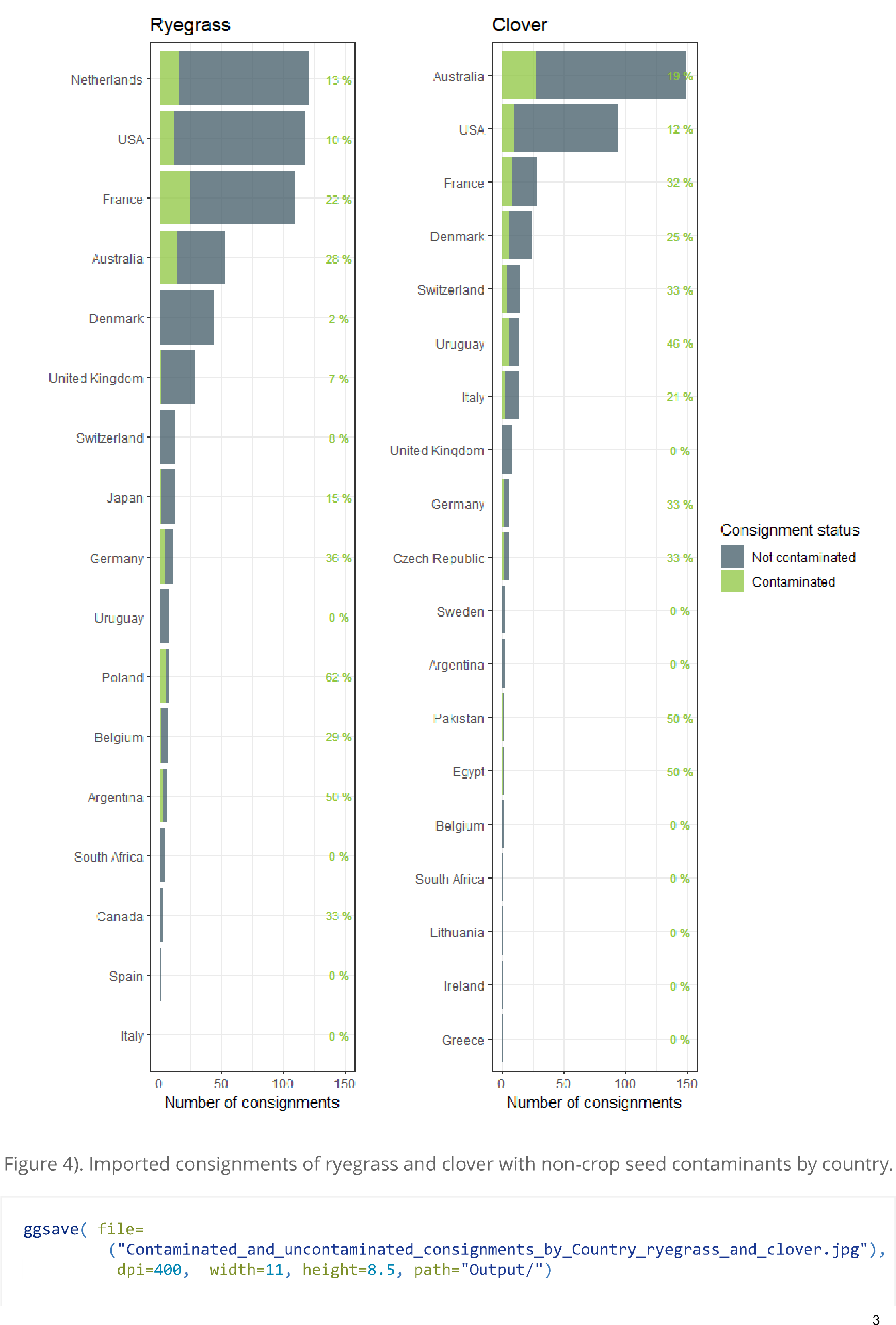


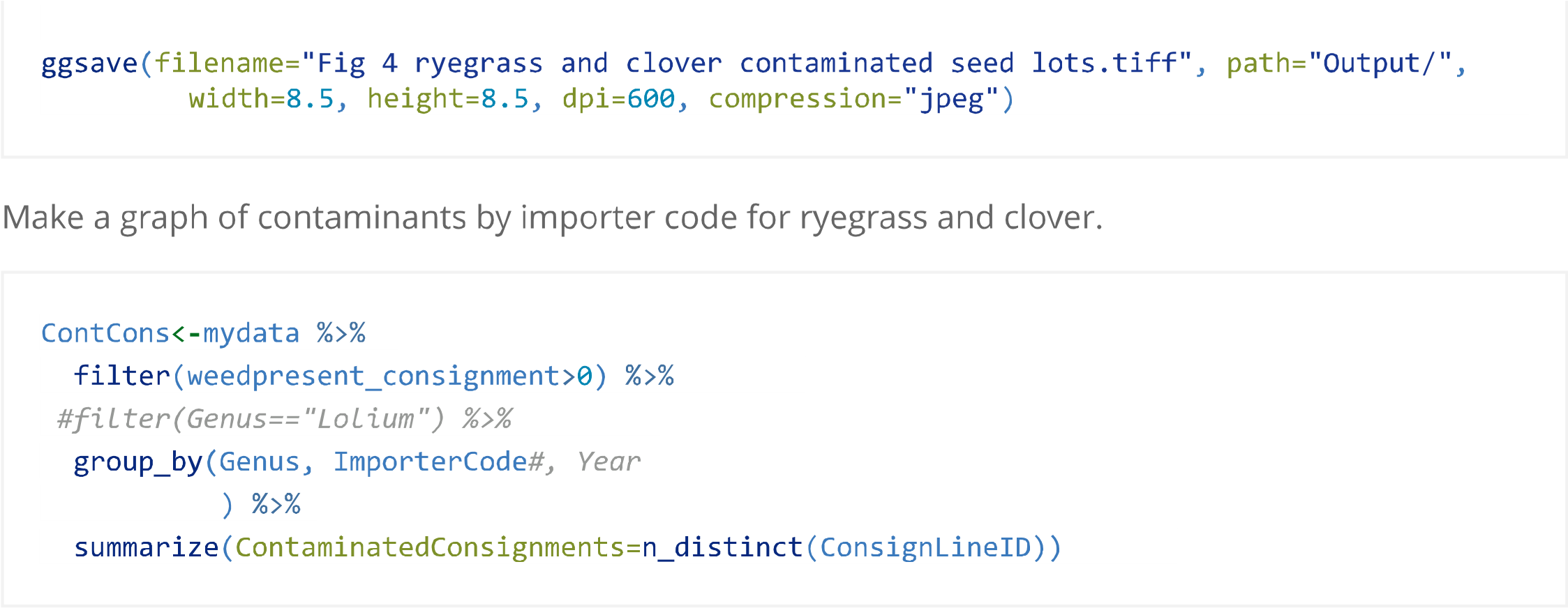


|  | | |
| --- | --- | --- |
|  | 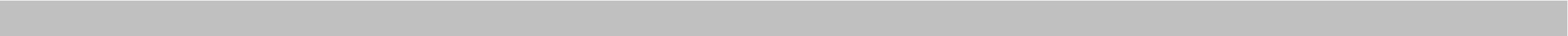 |  |


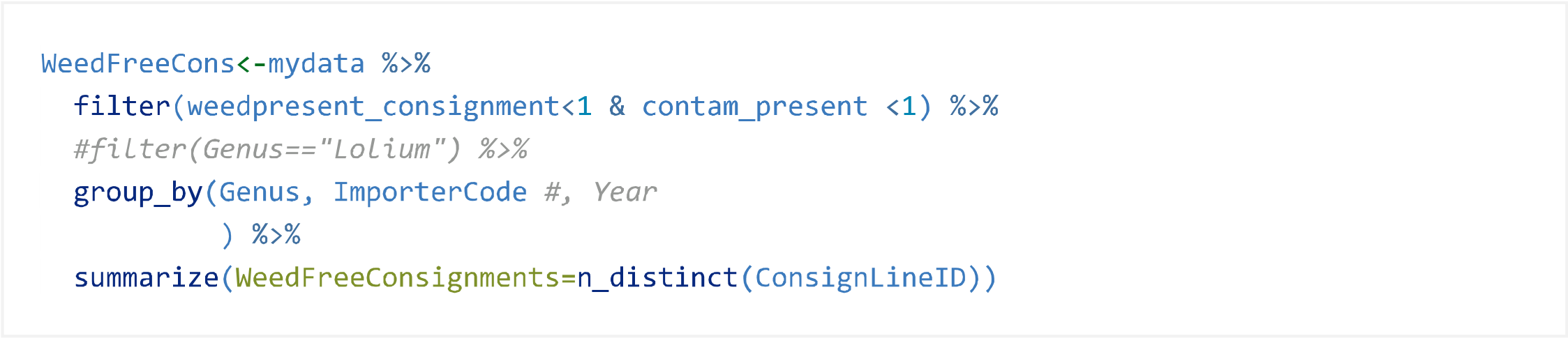


|  | | |
| --- | --- | --- |
|  | 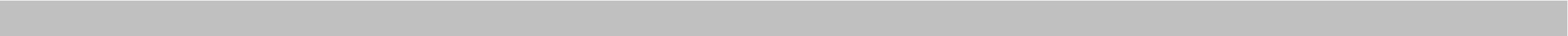 |  |


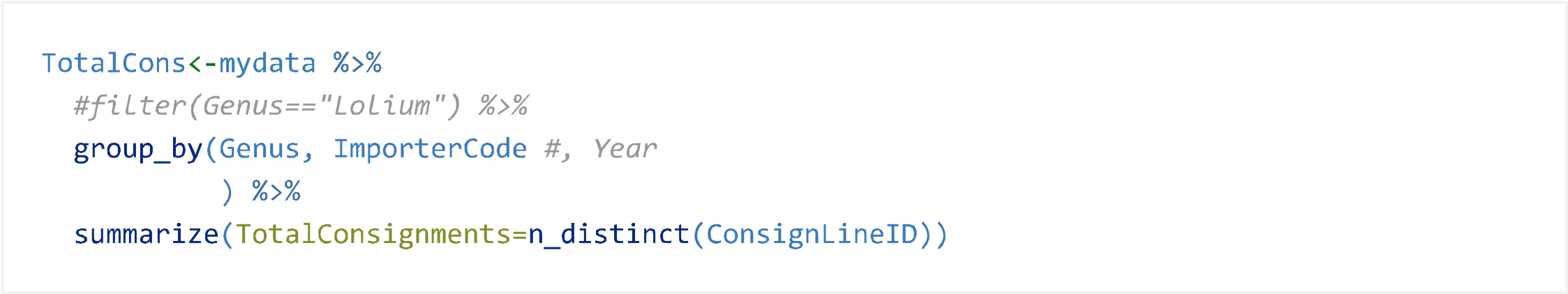


|  | | |
| --- | --- | --- |
|  | 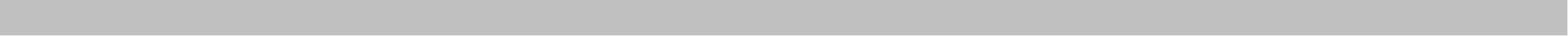 |  |


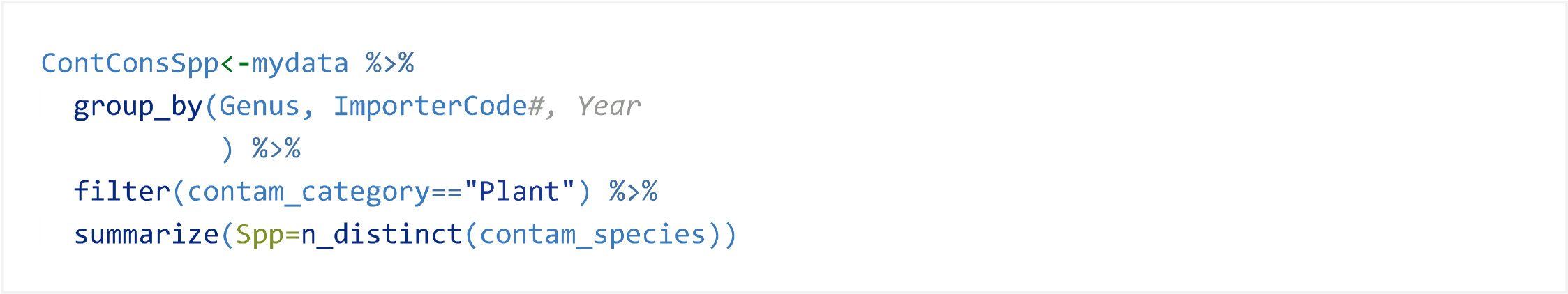


|  | | |
| --- | --- | --- |
|  | 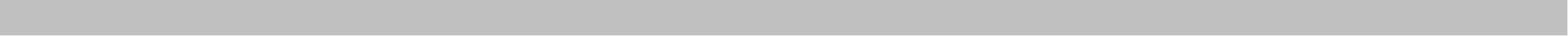 |  |


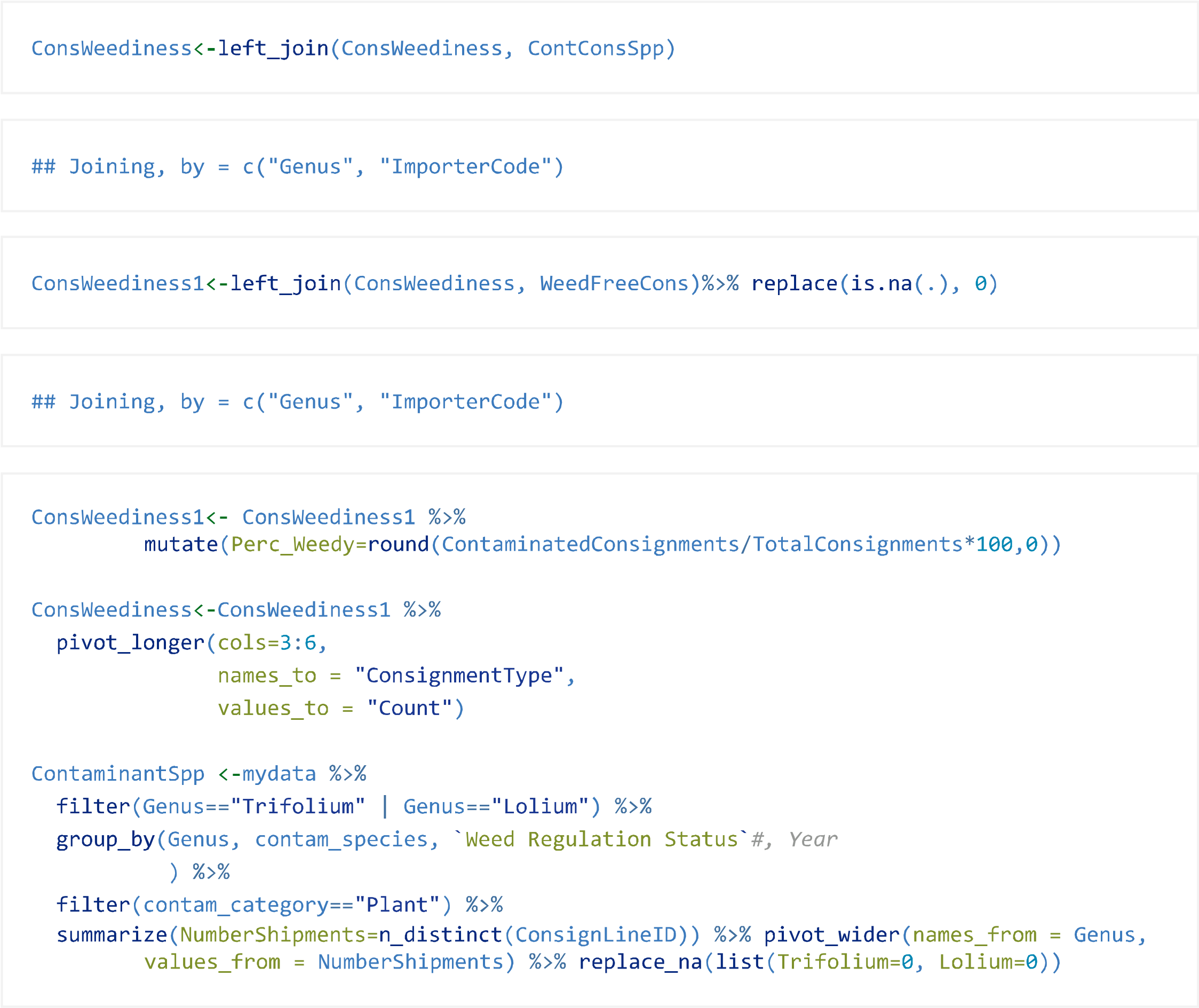


|  | | |
| --- | --- | --- |
|  | 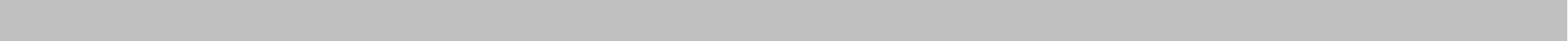 |  |


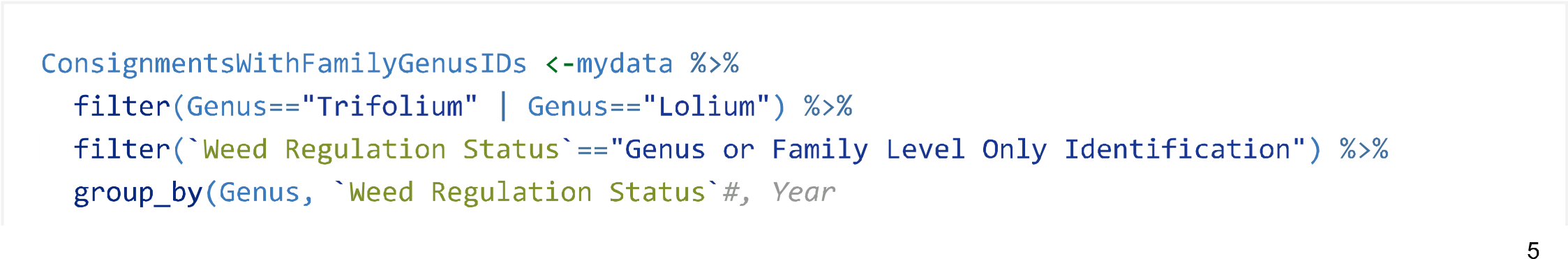


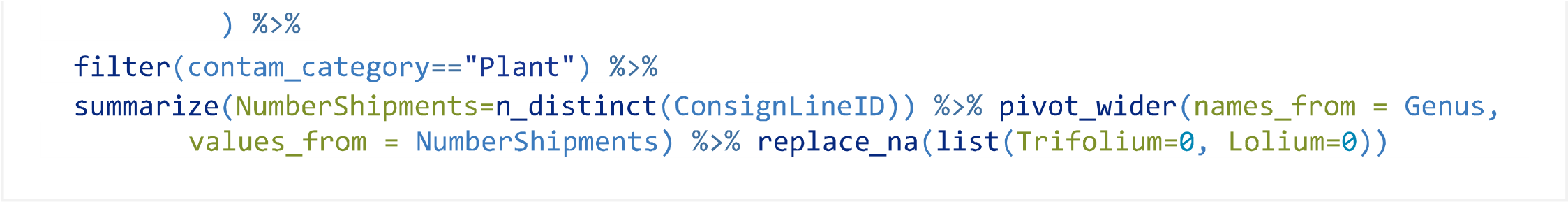


|  | | |
| --- | --- | --- |
|  | 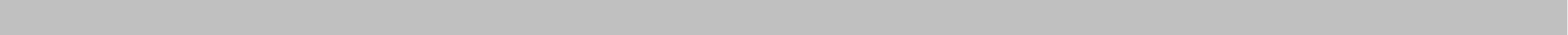 |  |


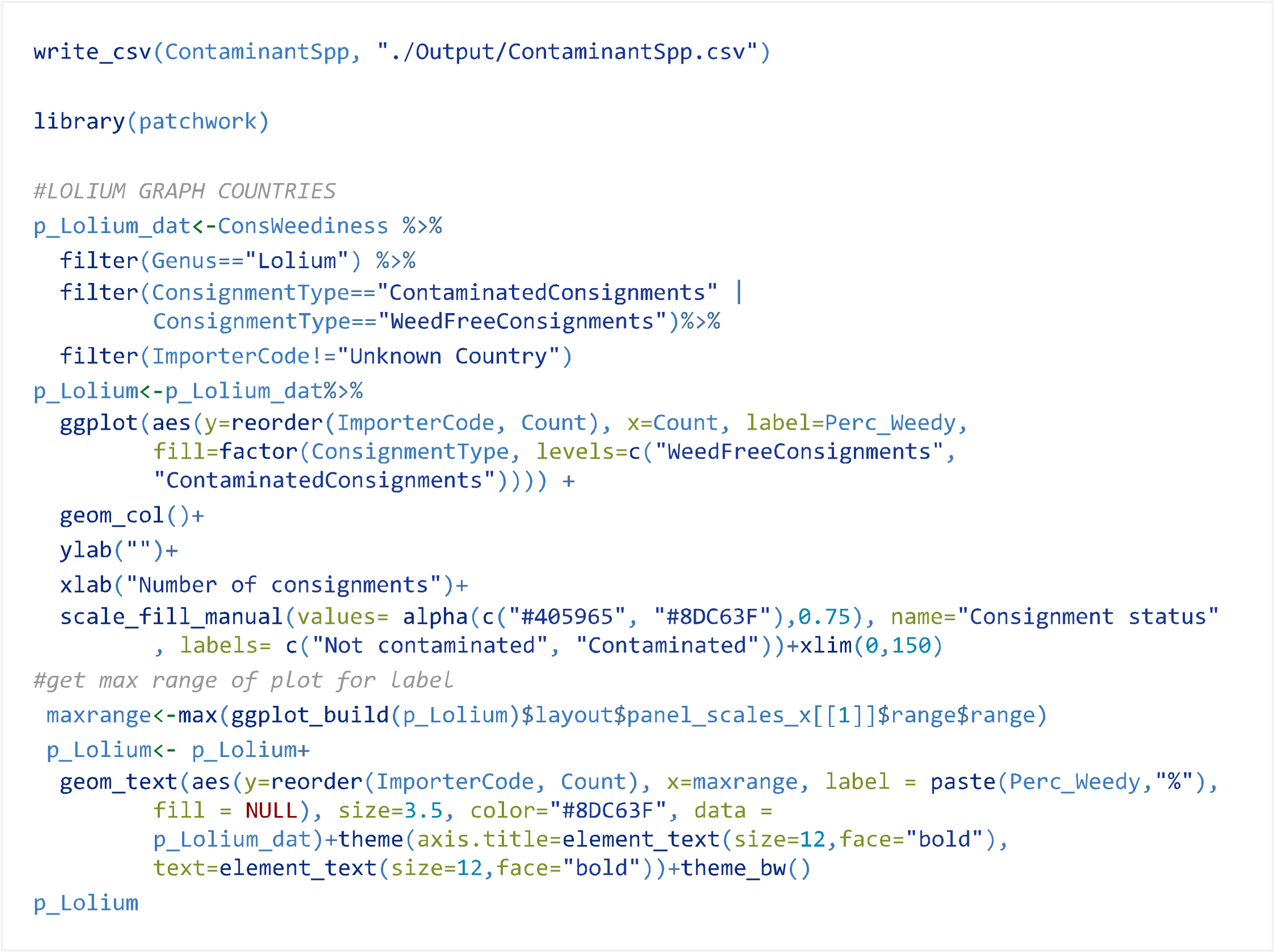


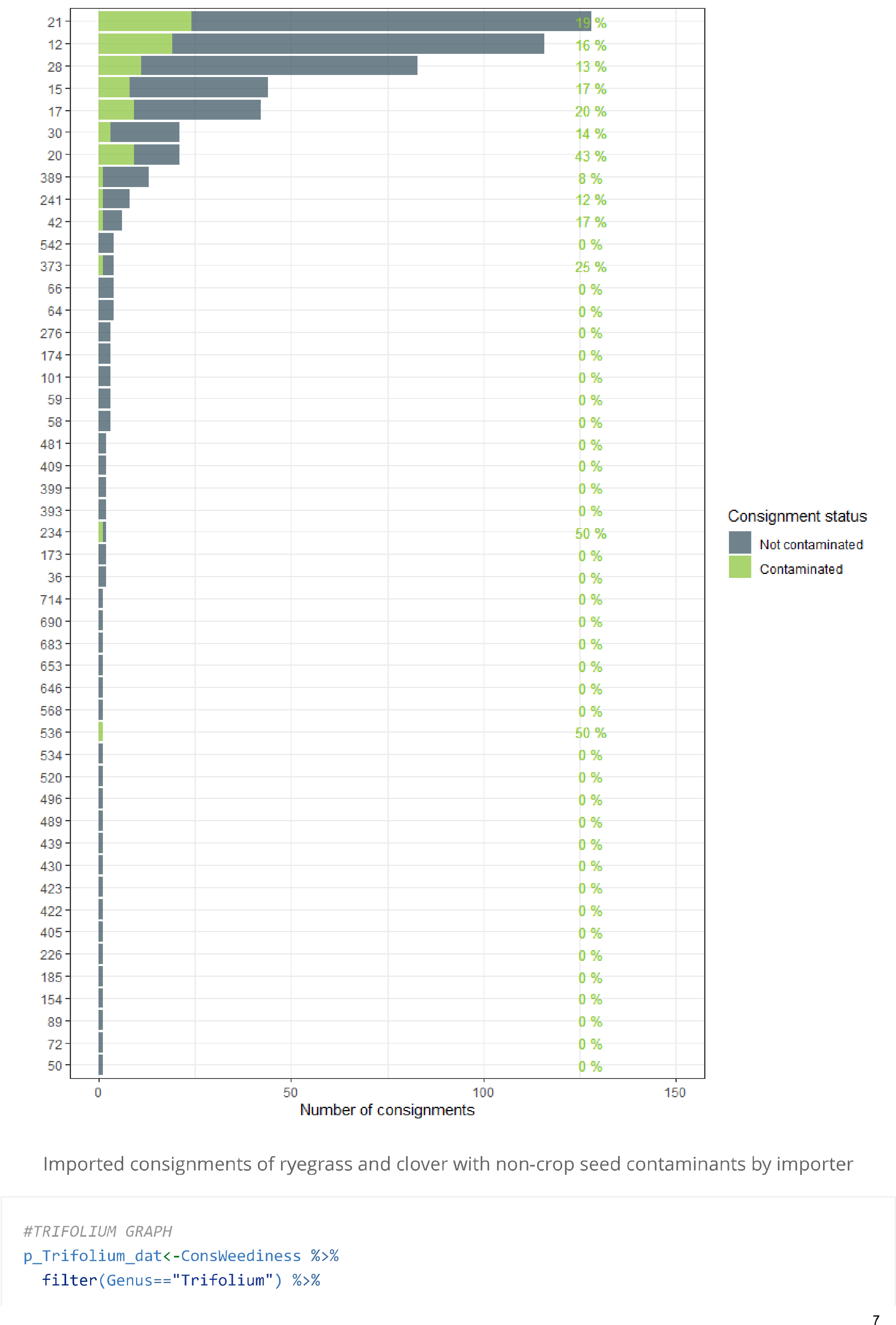


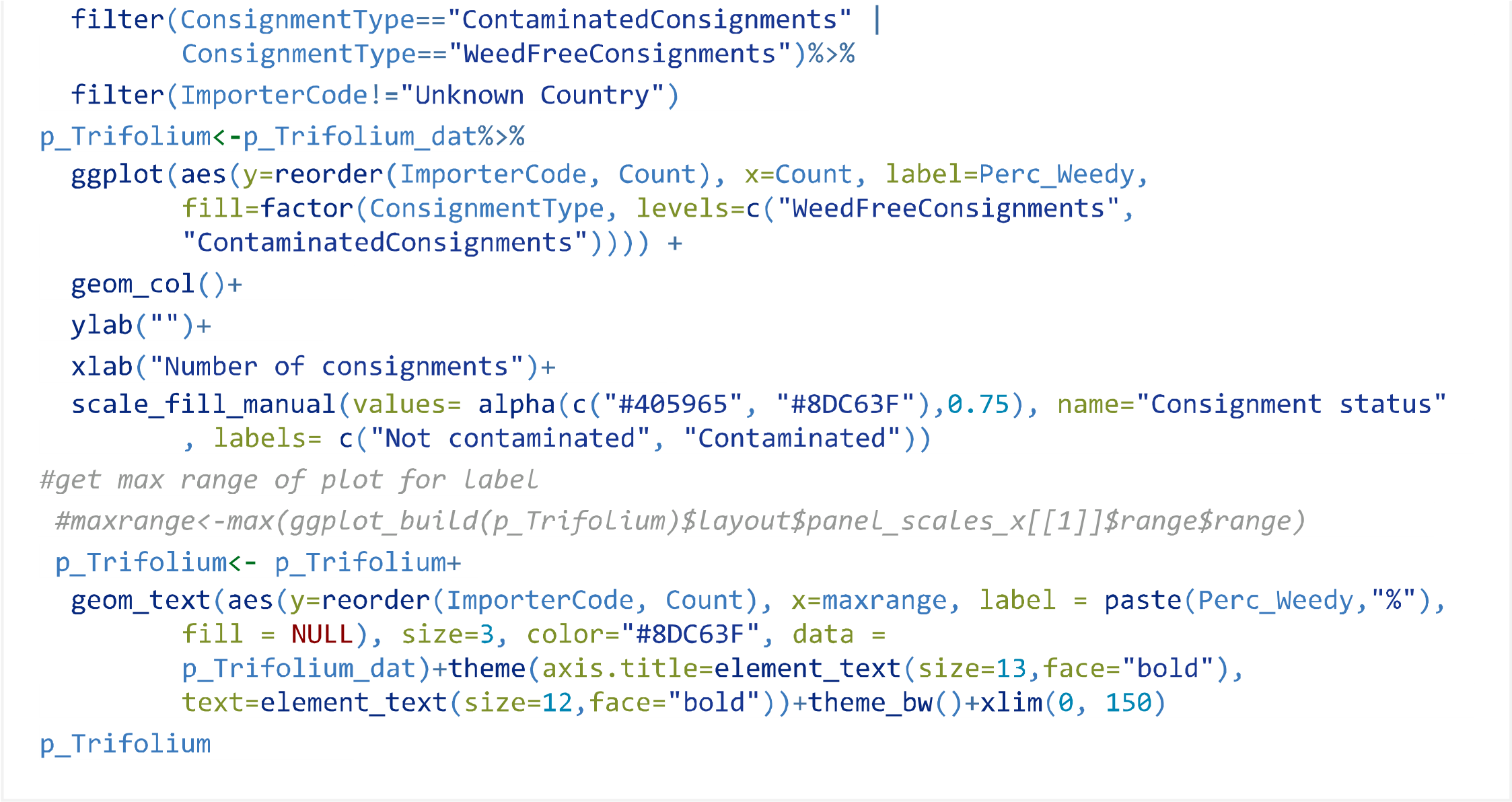


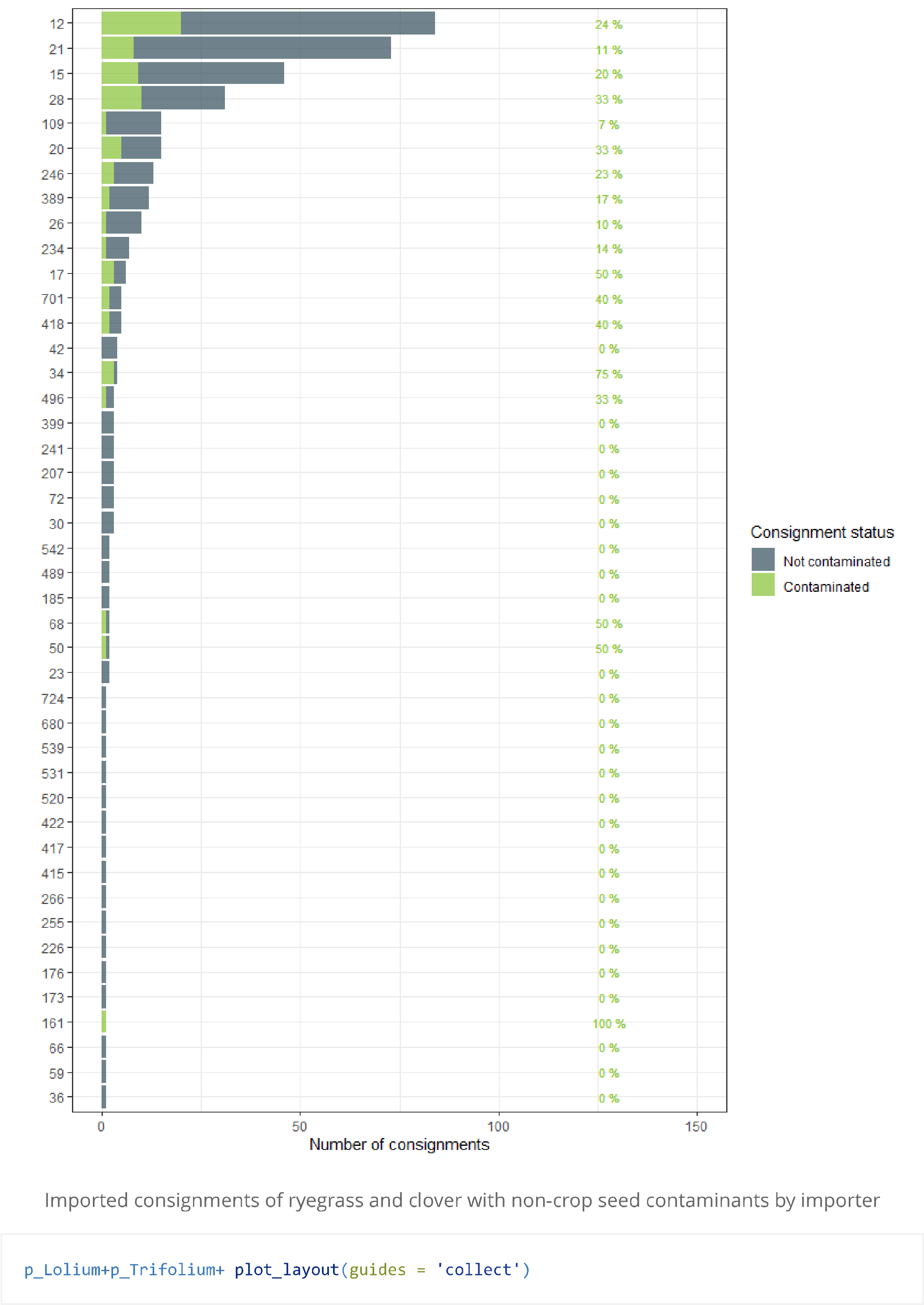


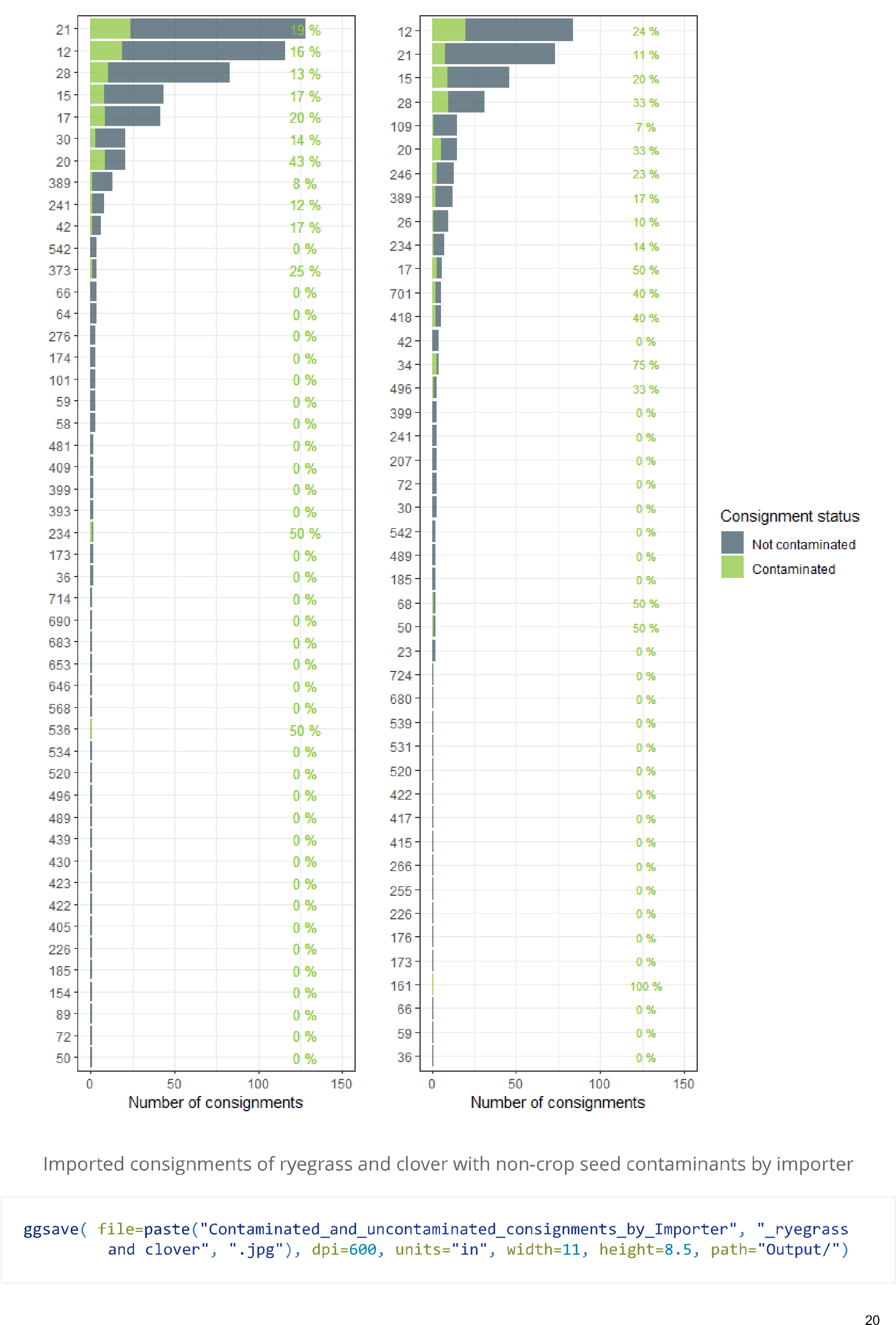


### Ryegrass hypothetical contaminant spread simulations.


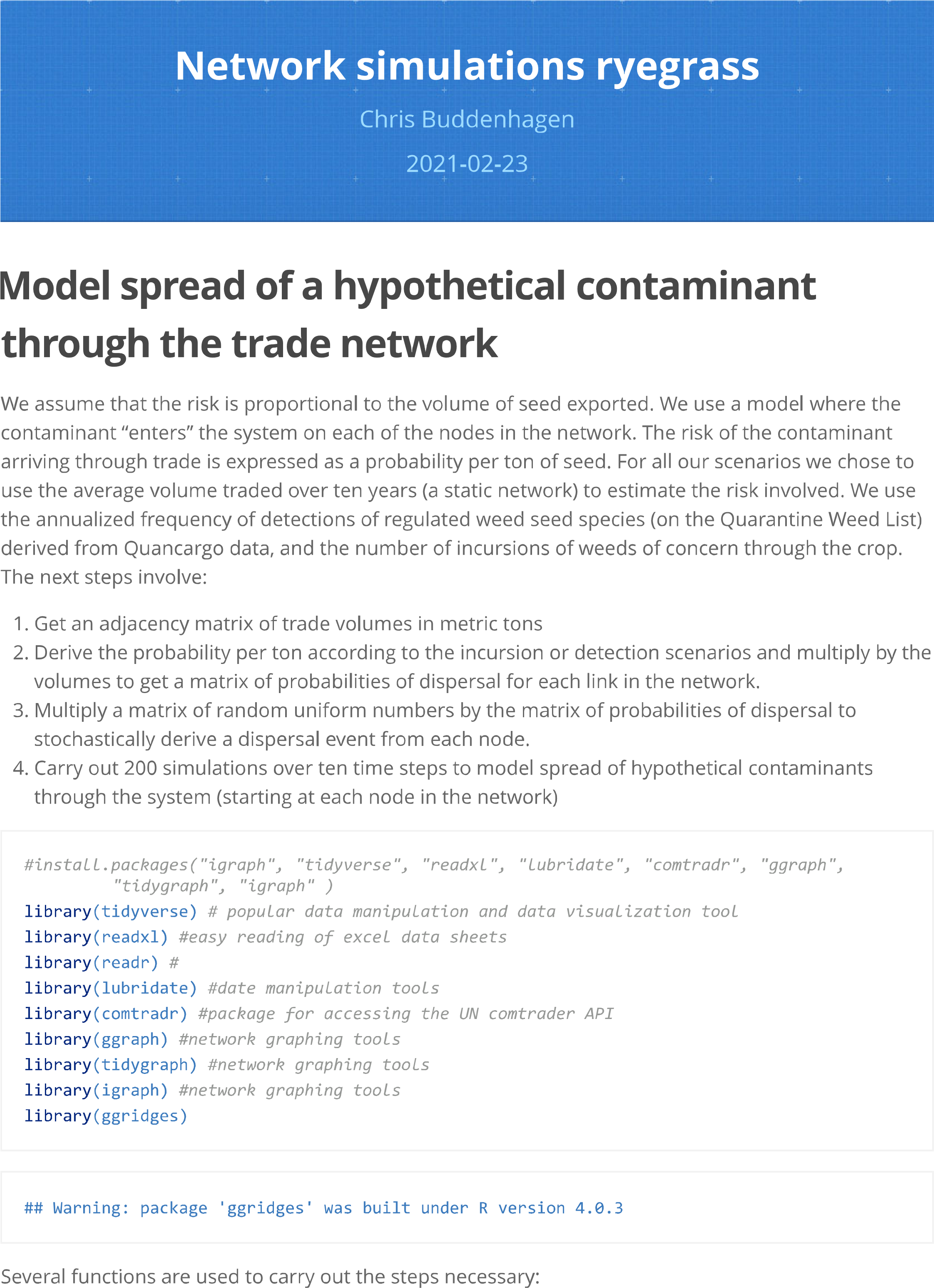


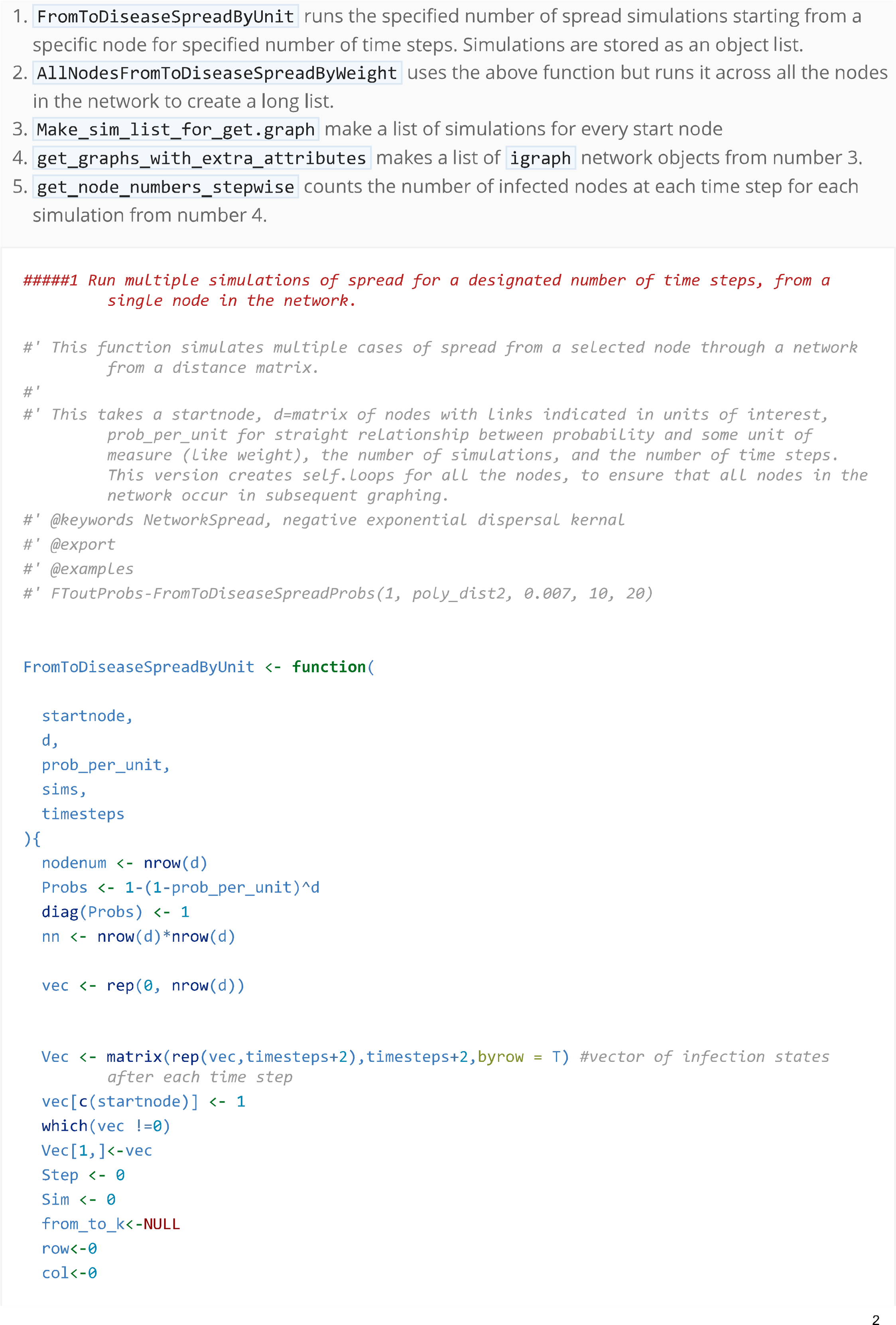


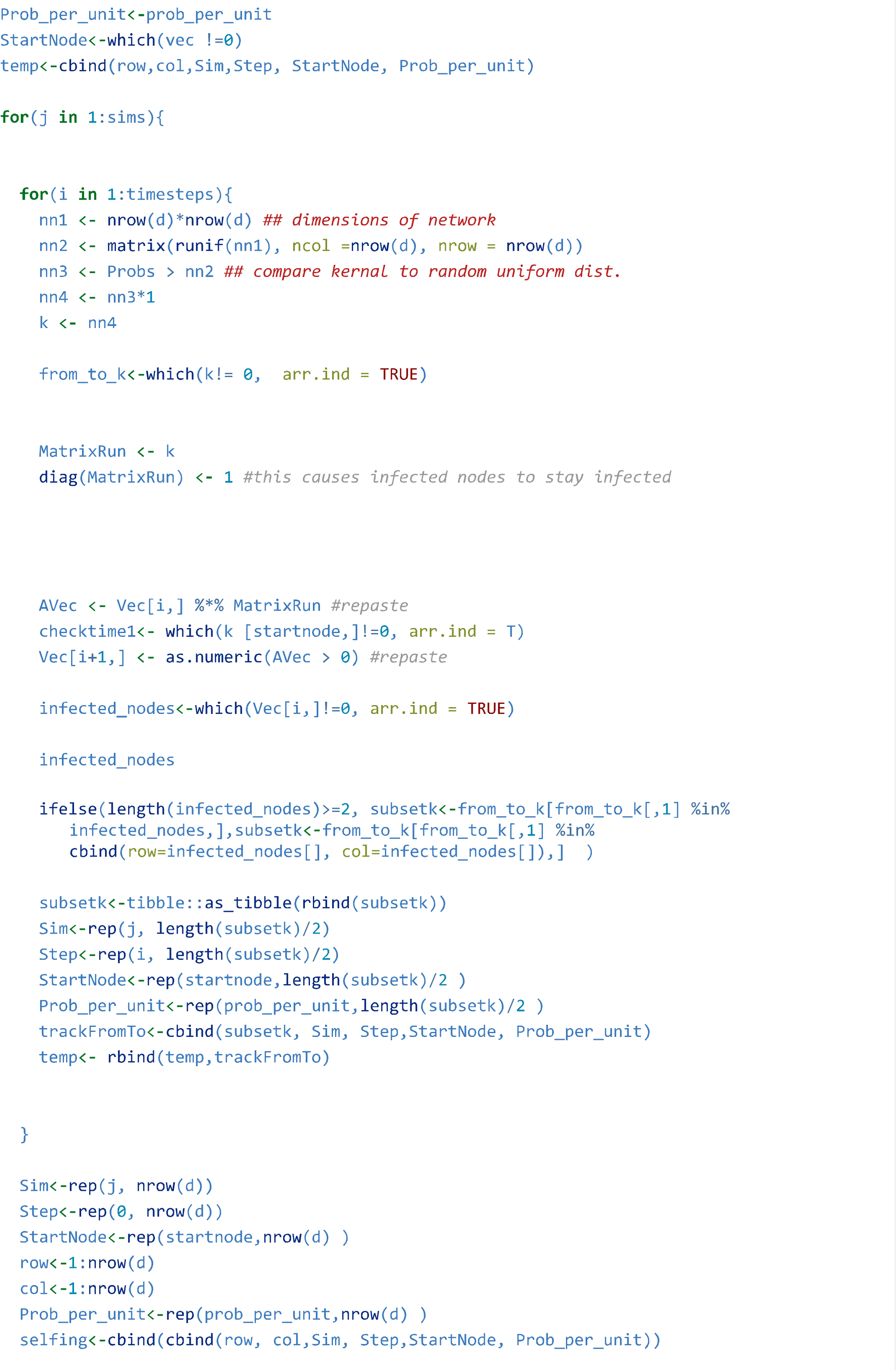


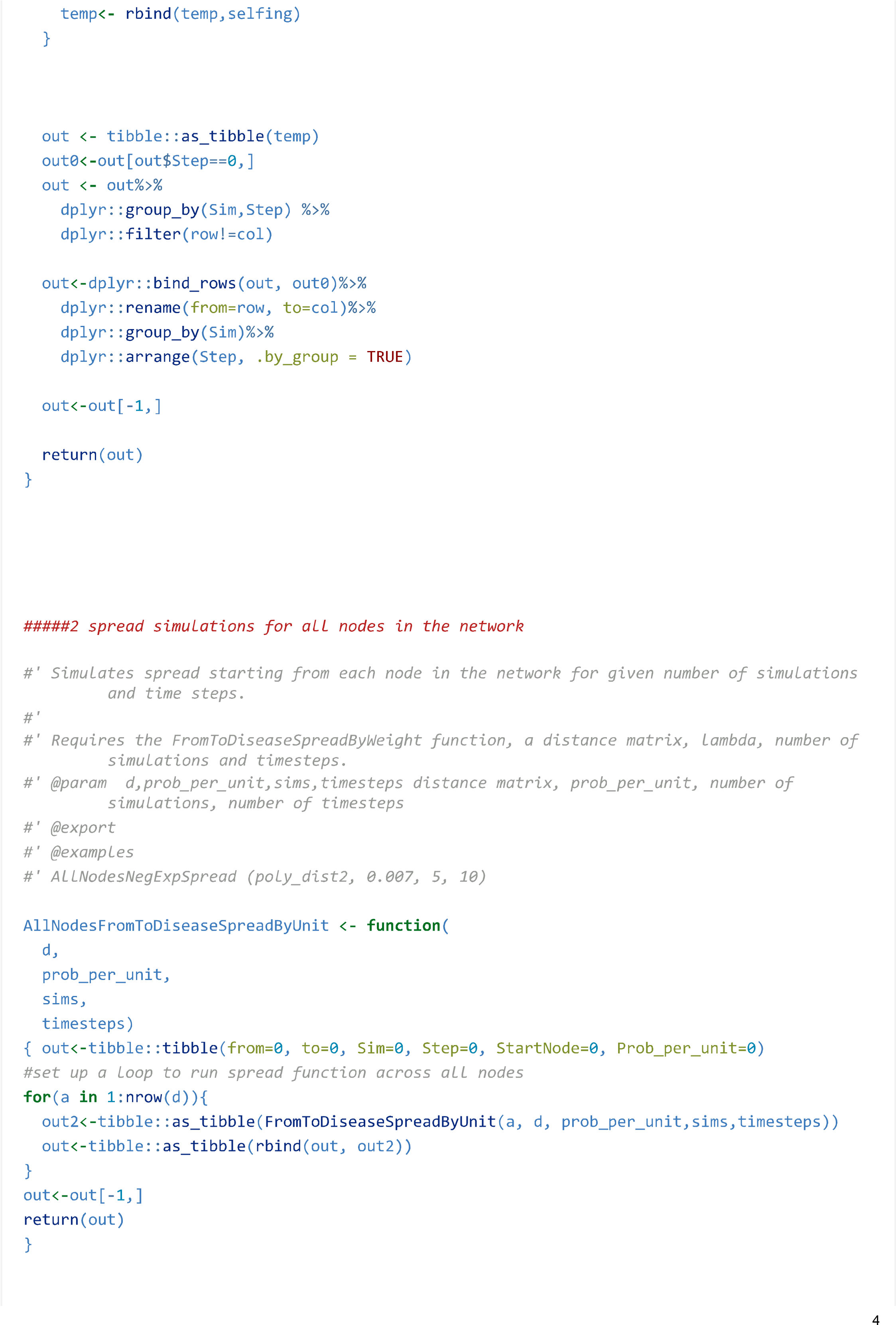


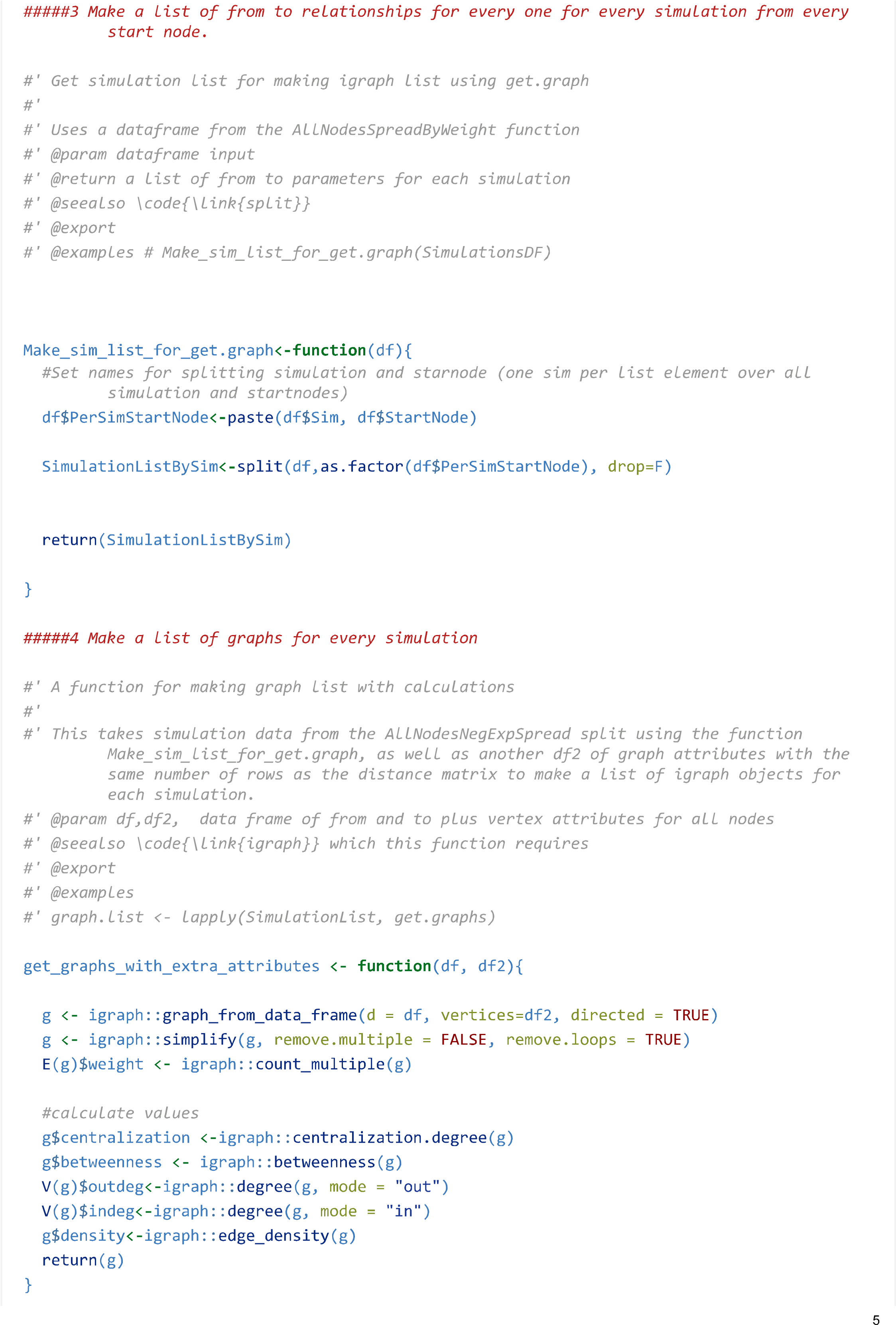


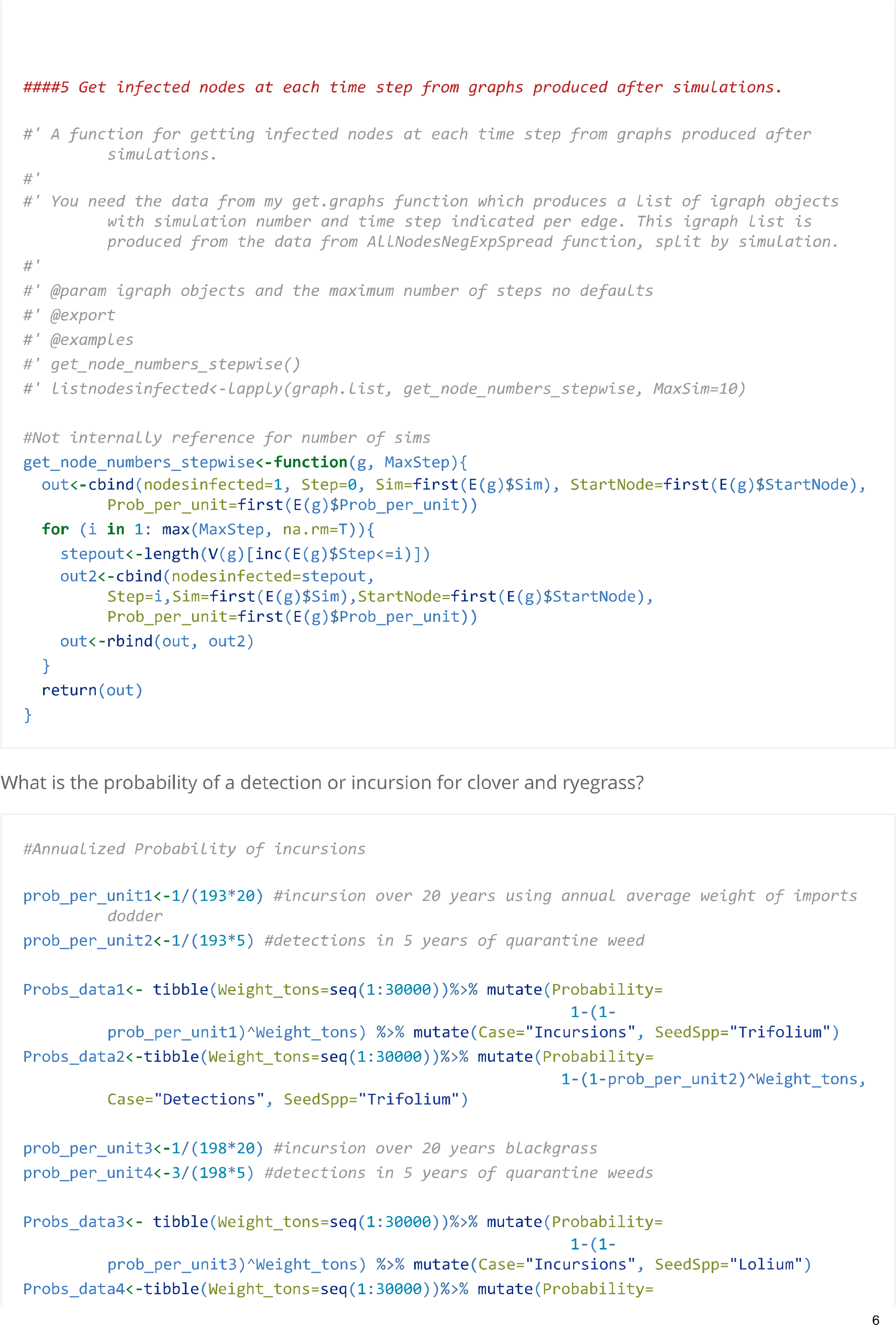


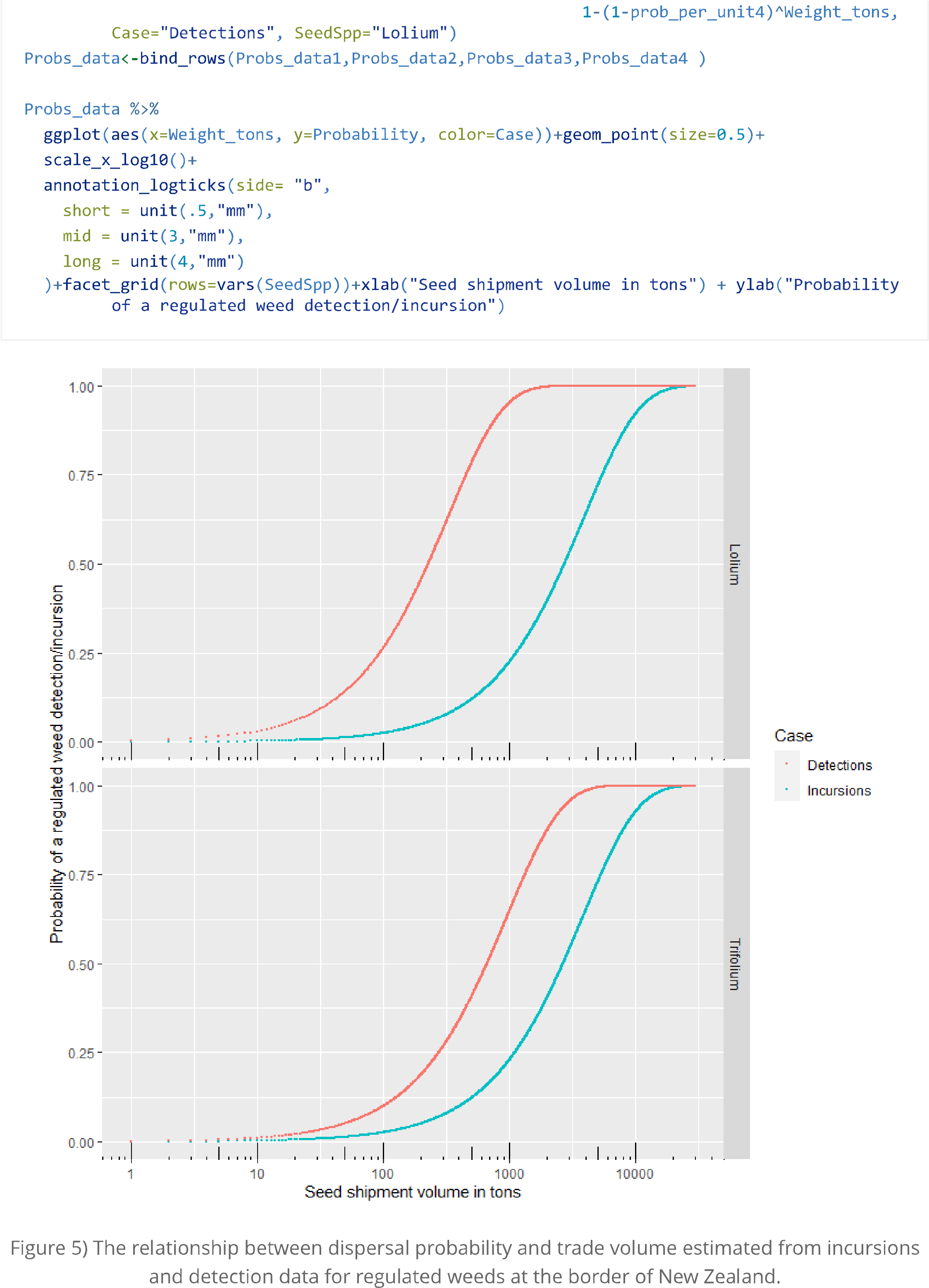


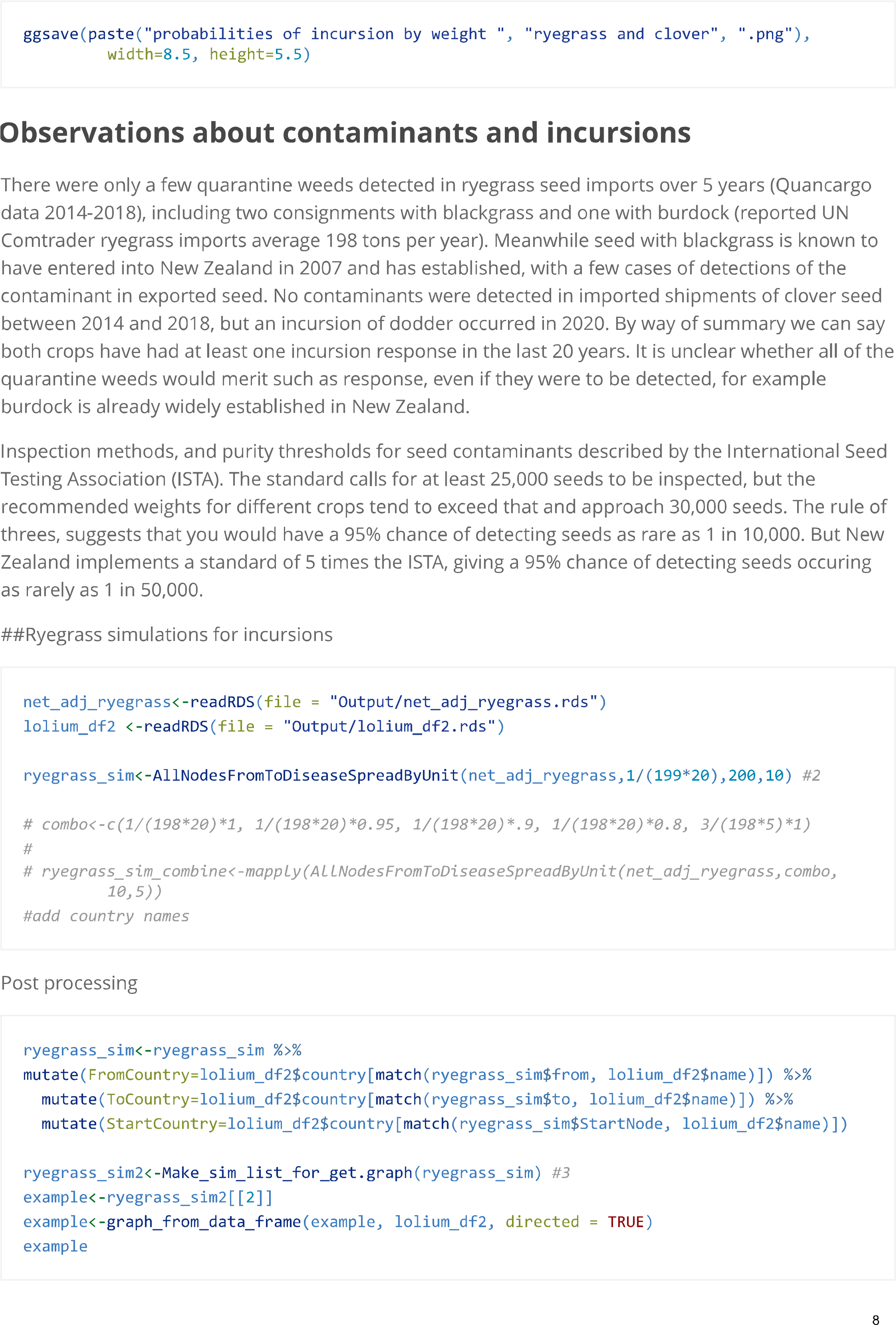


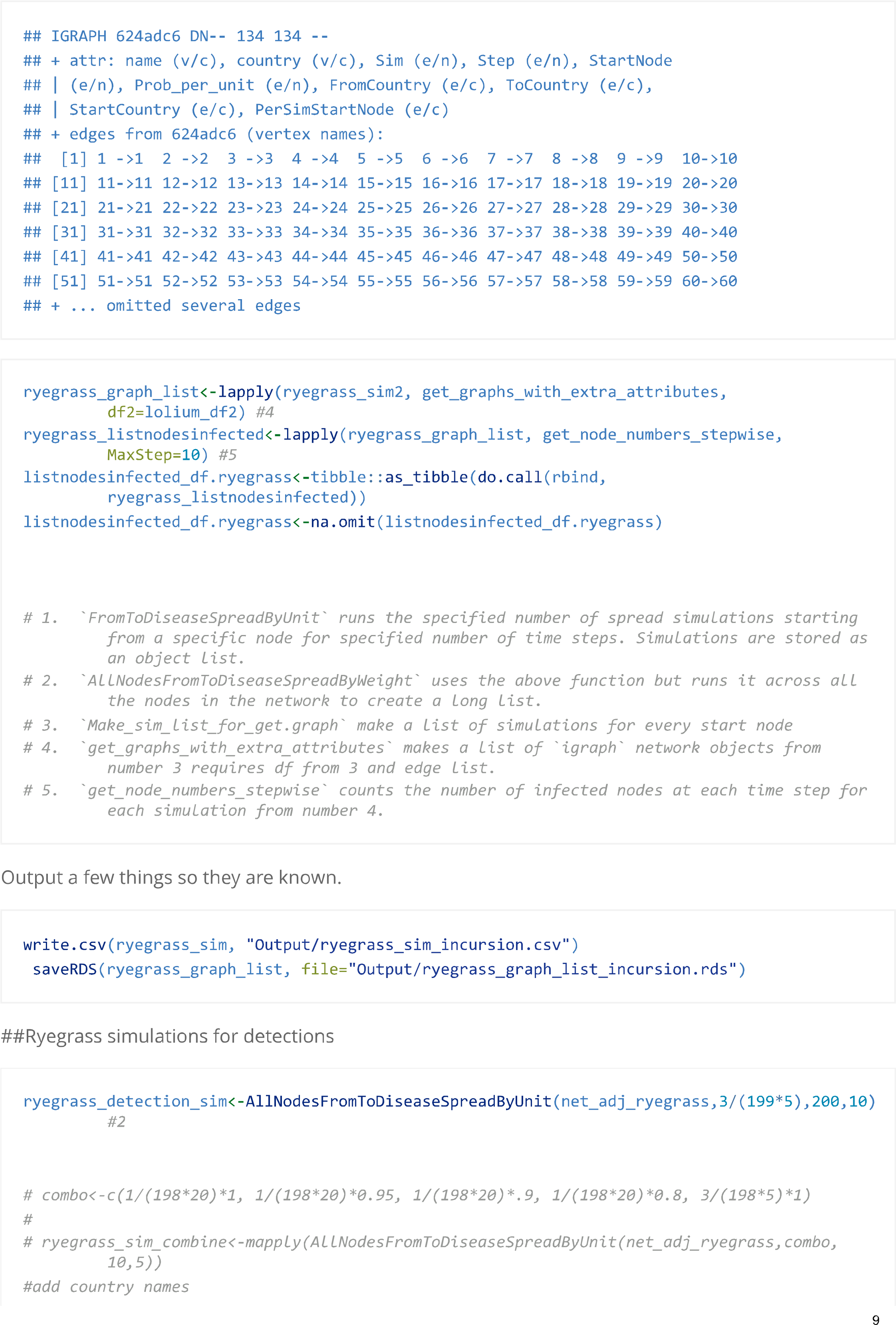


|  | | |
| --- | --- | --- |
|  | 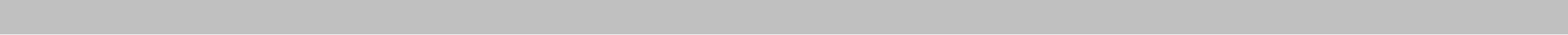 |  |


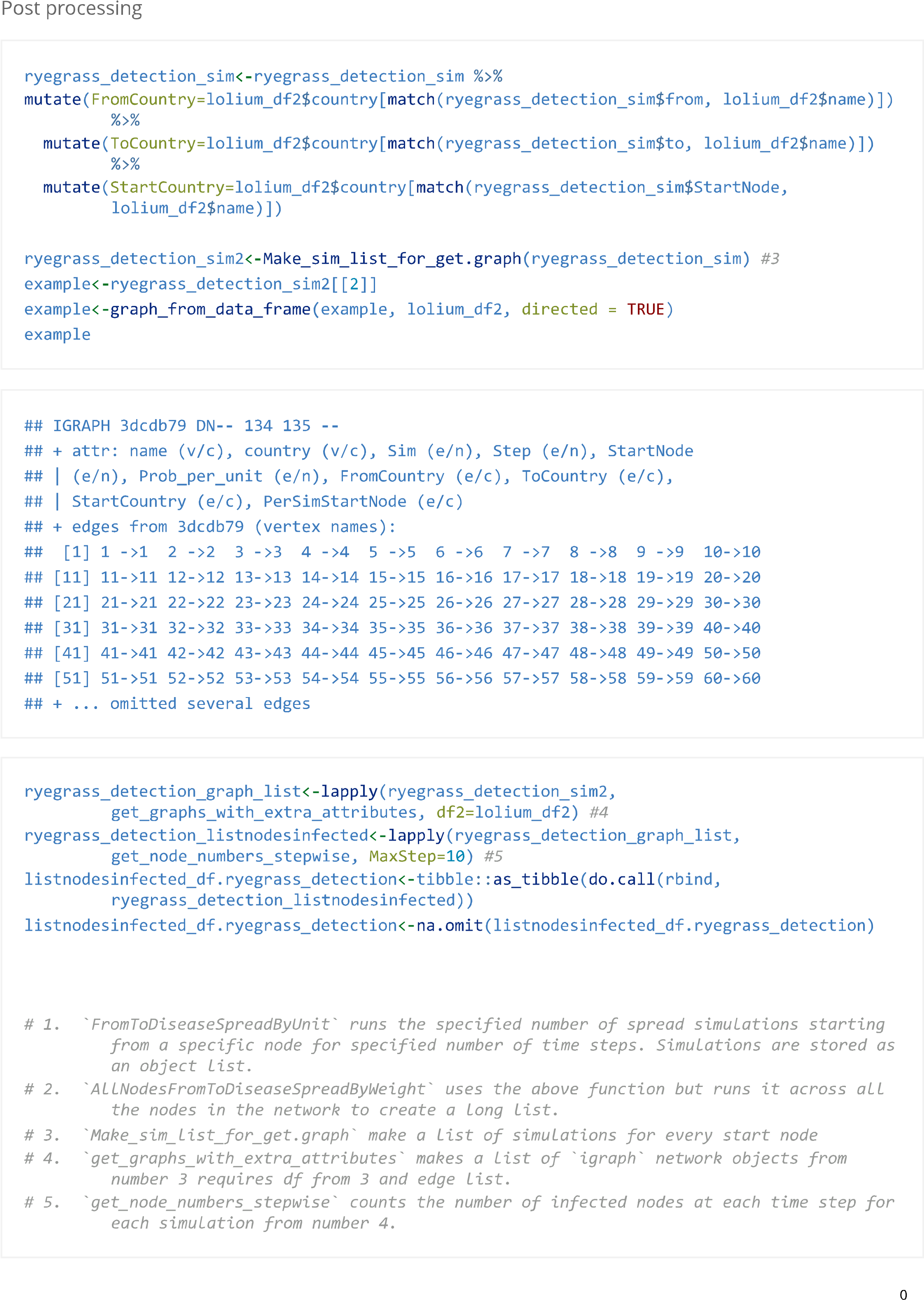


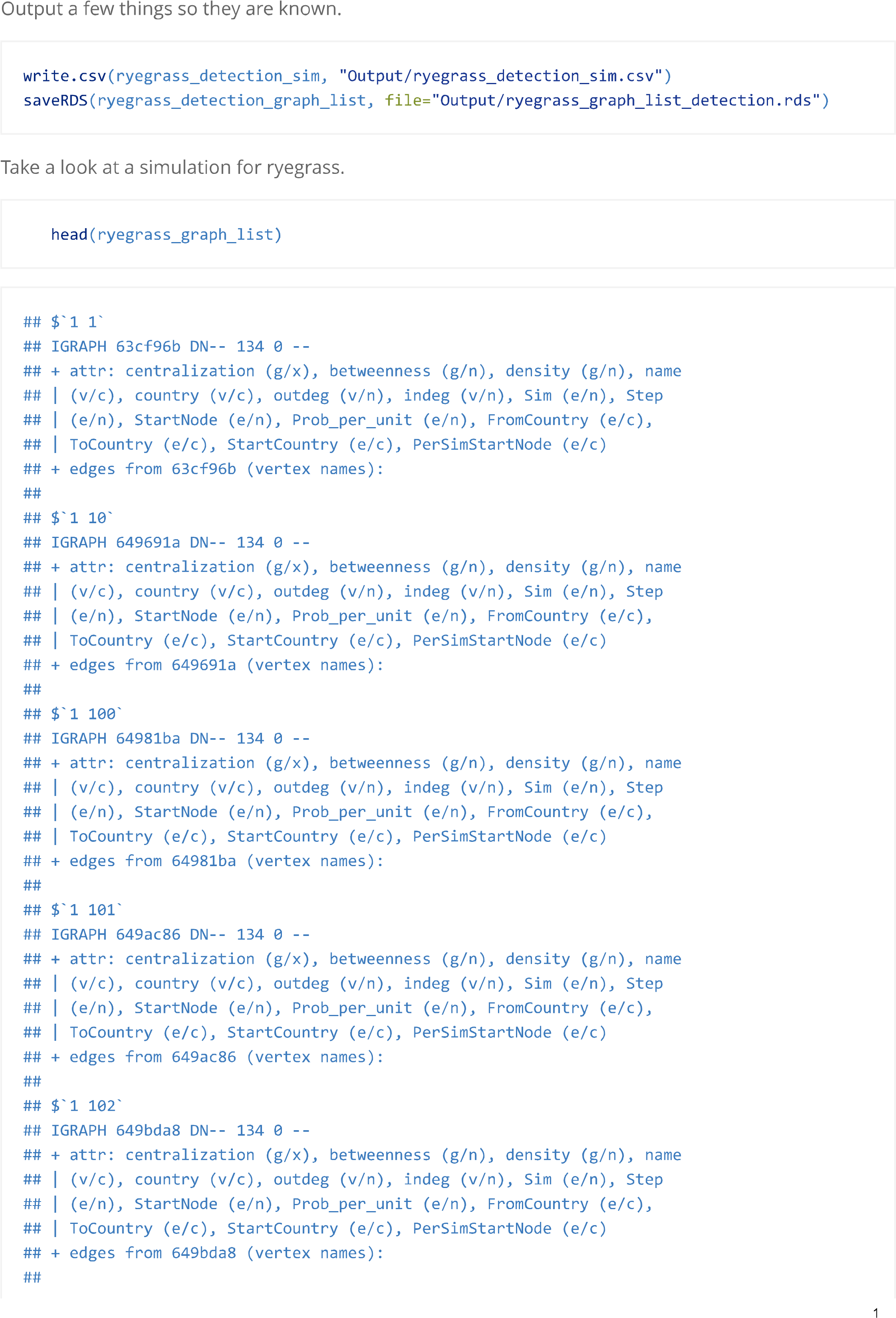


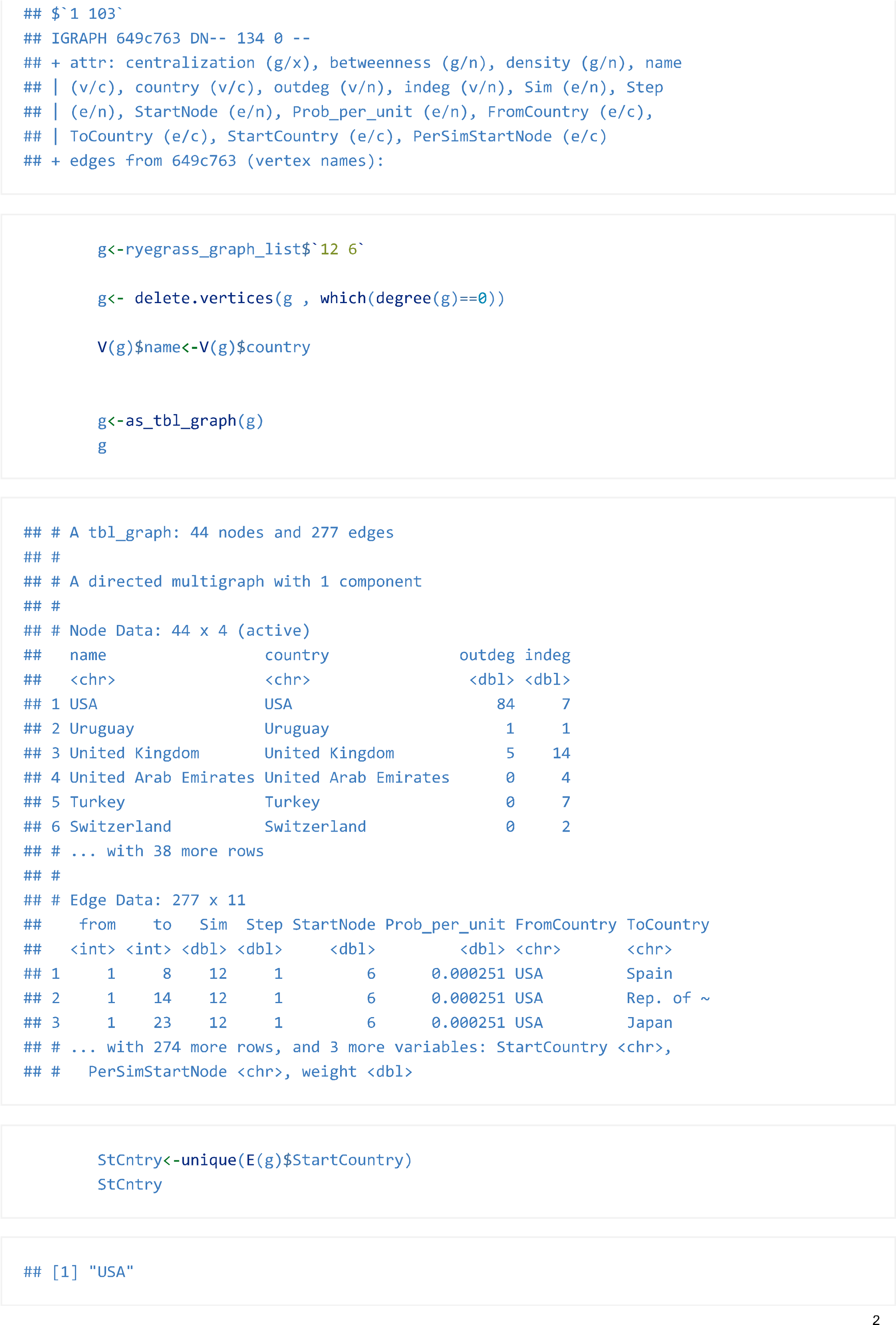


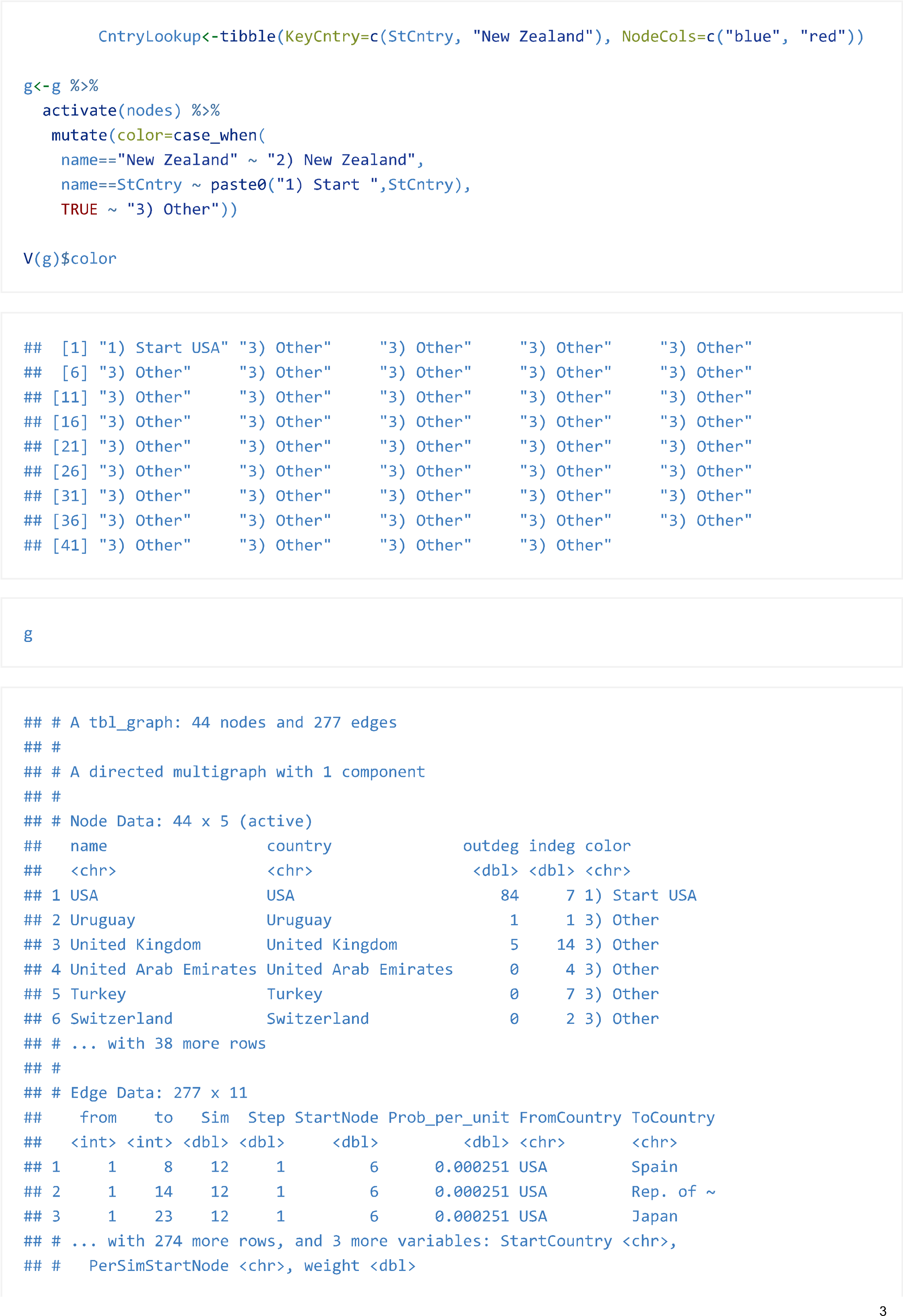


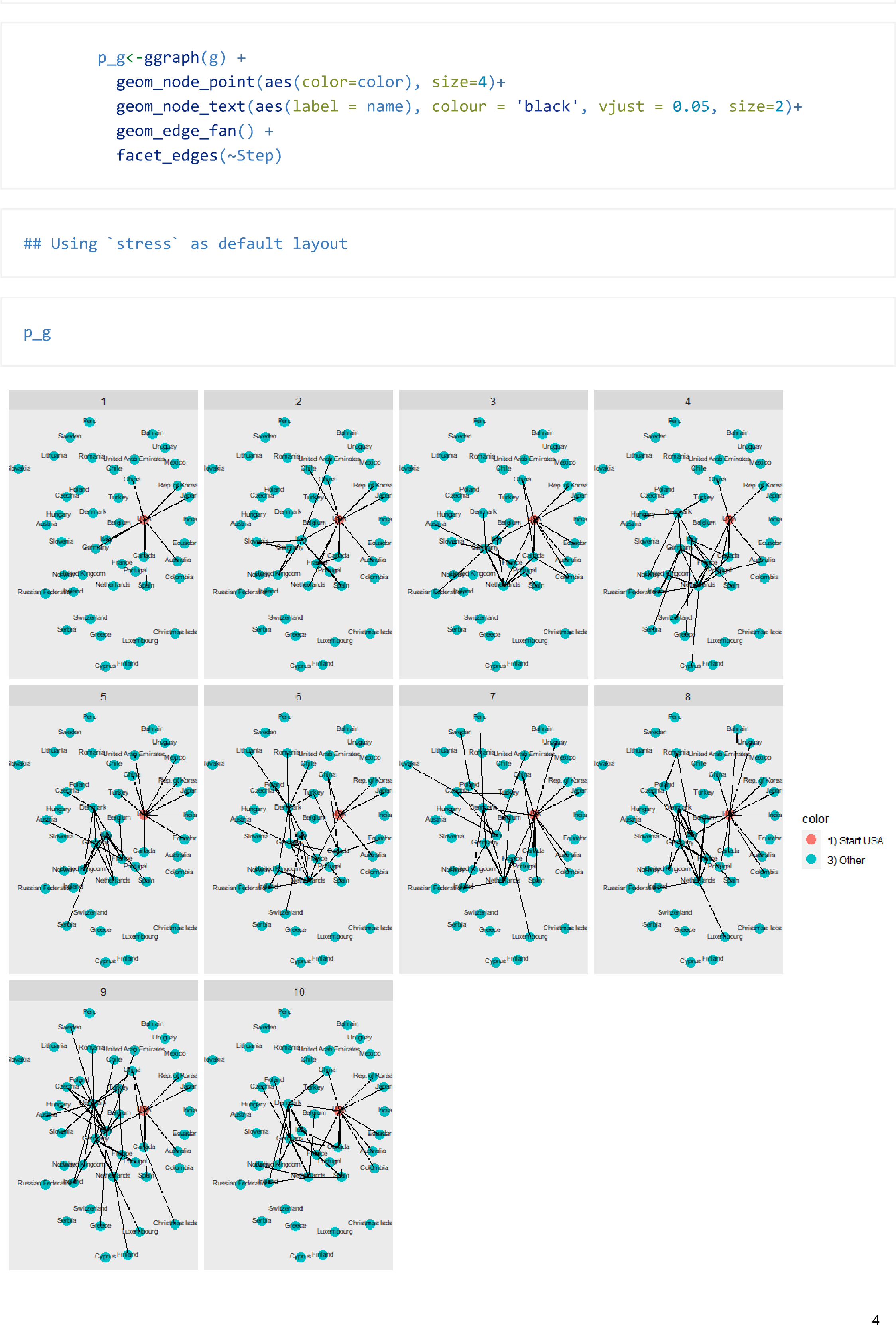


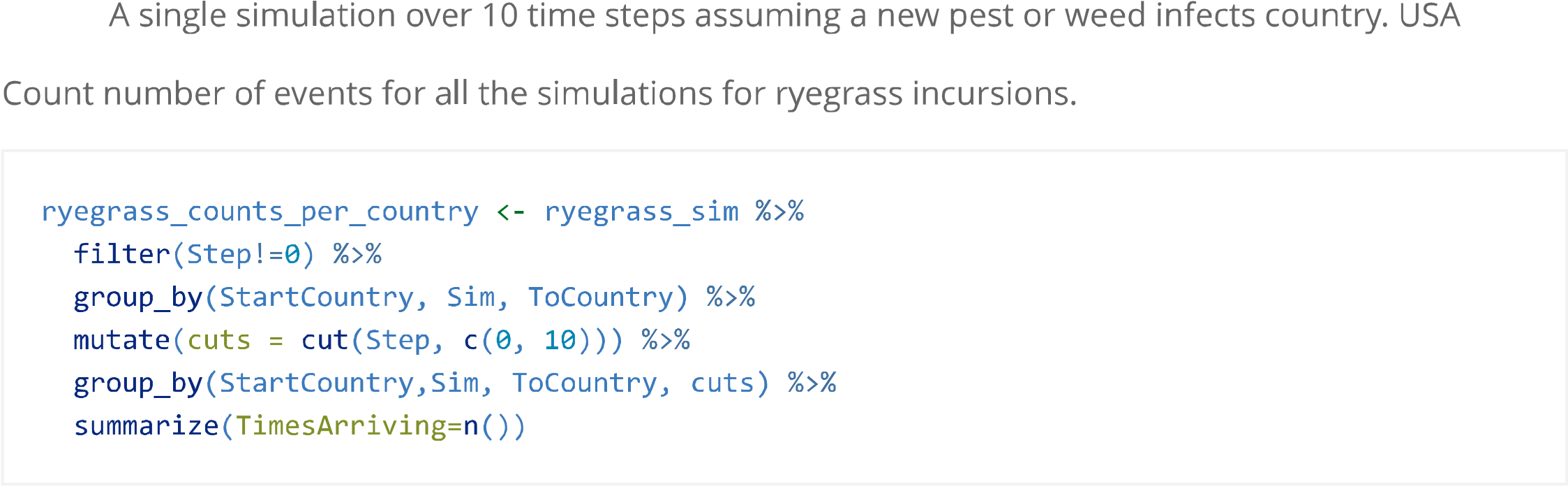


|  | | |
| --- | --- | --- |
|  | 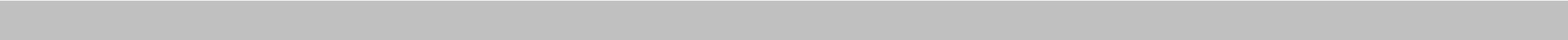 |  |


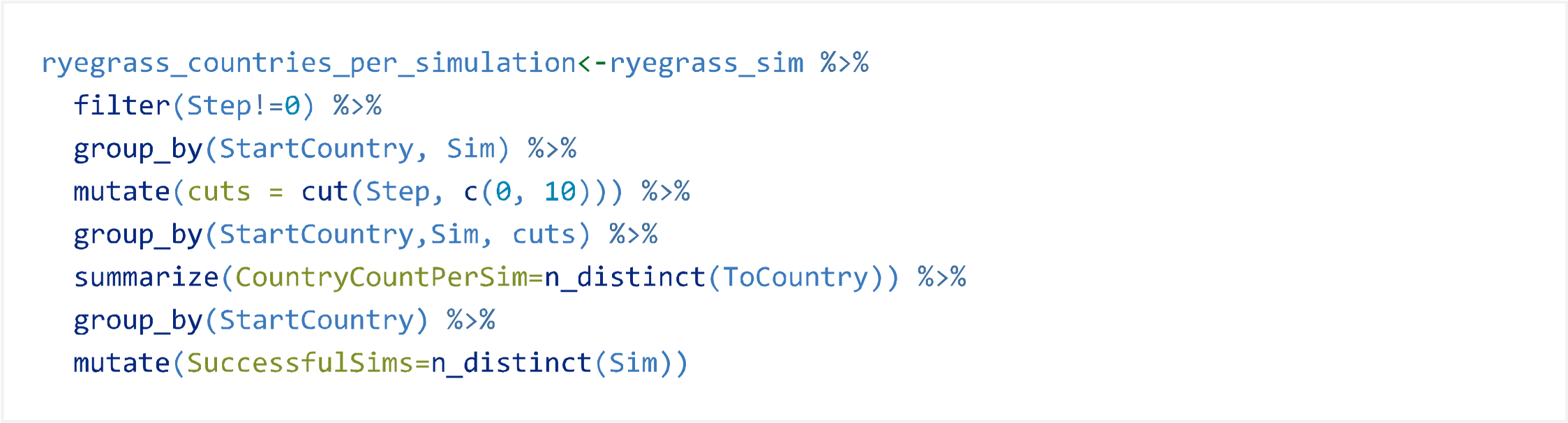


|  | | |
| --- | --- | --- |
|  | 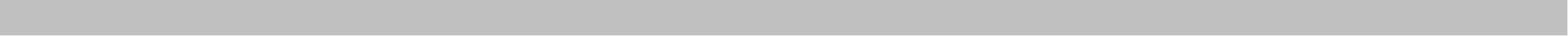 |  |


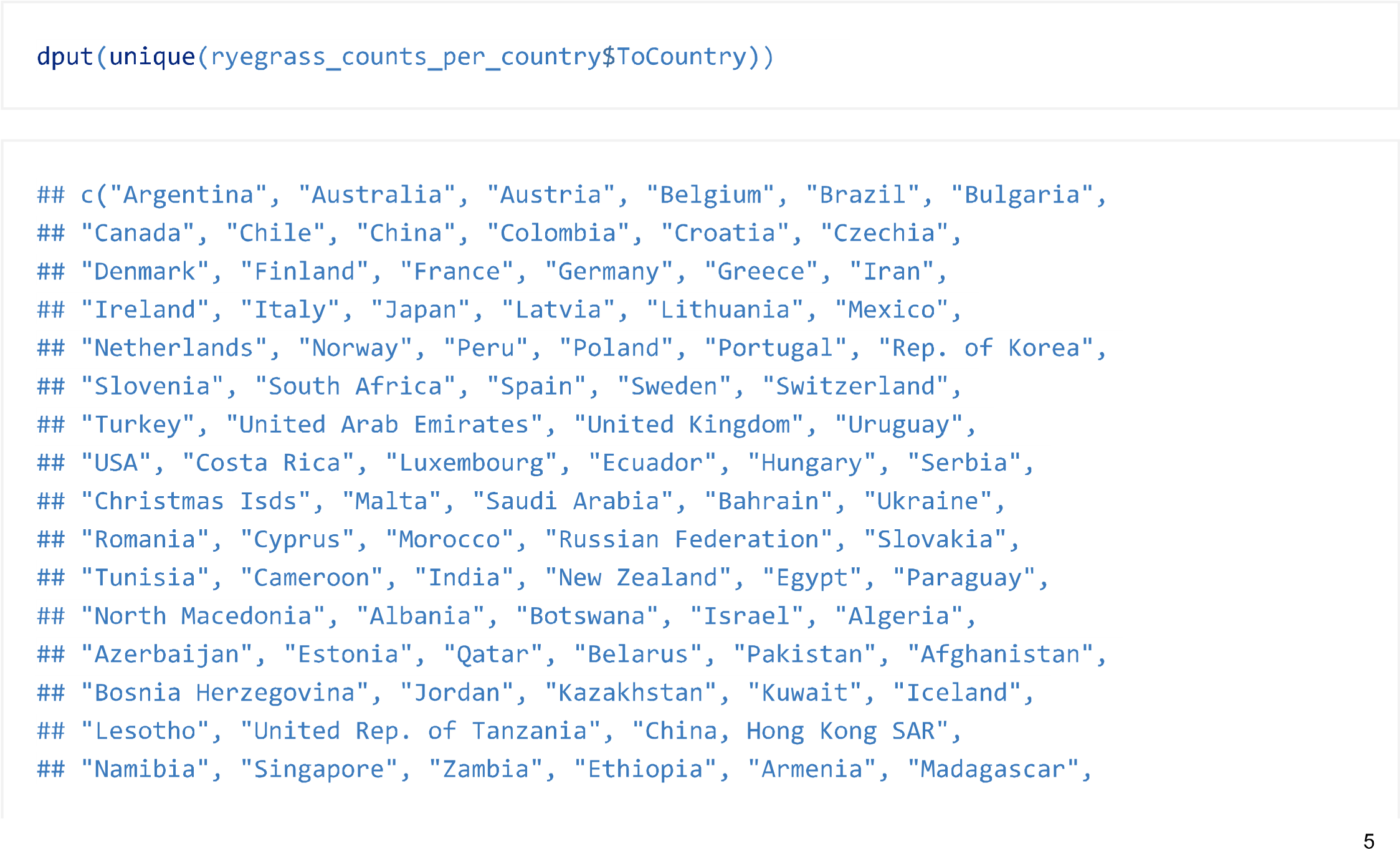


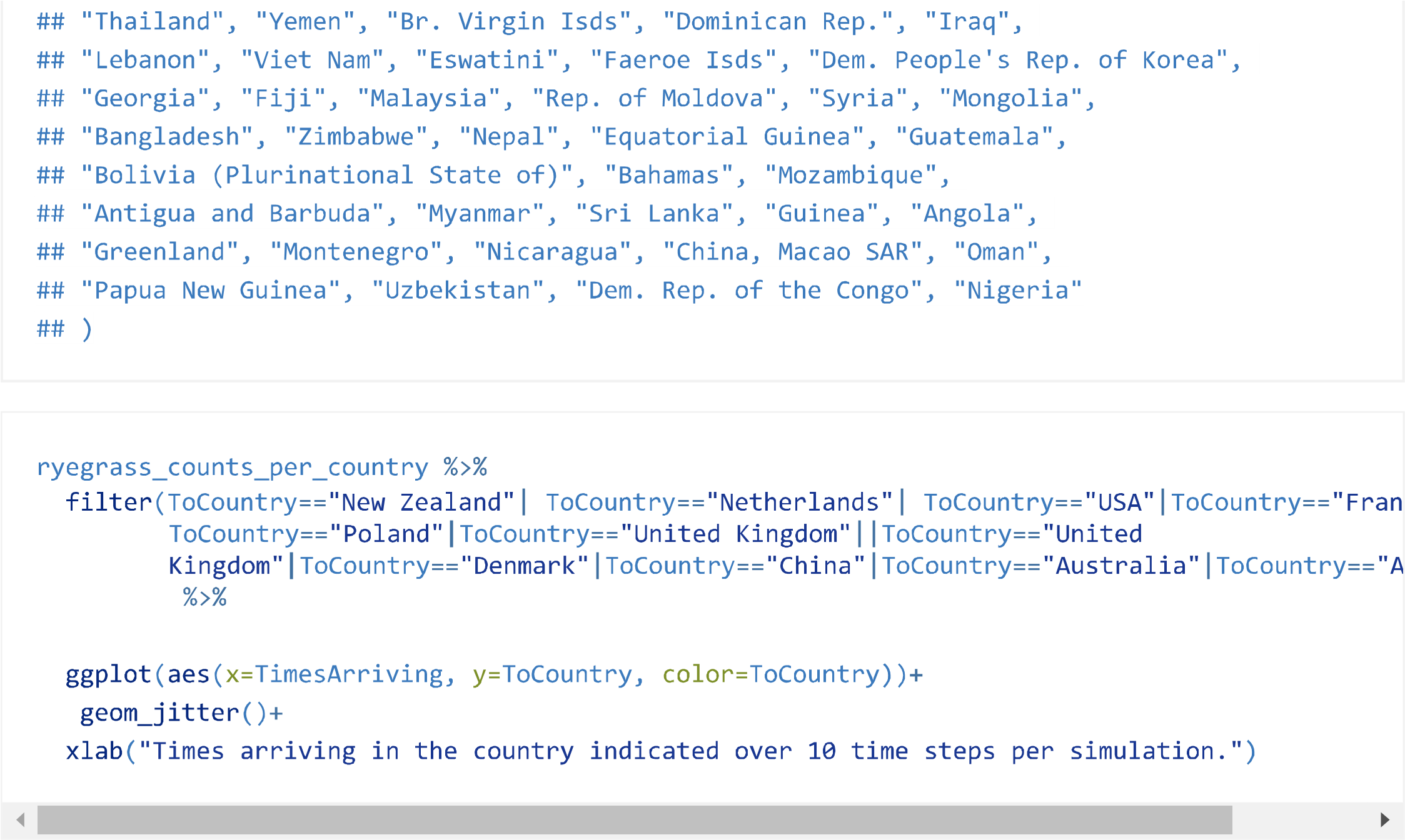


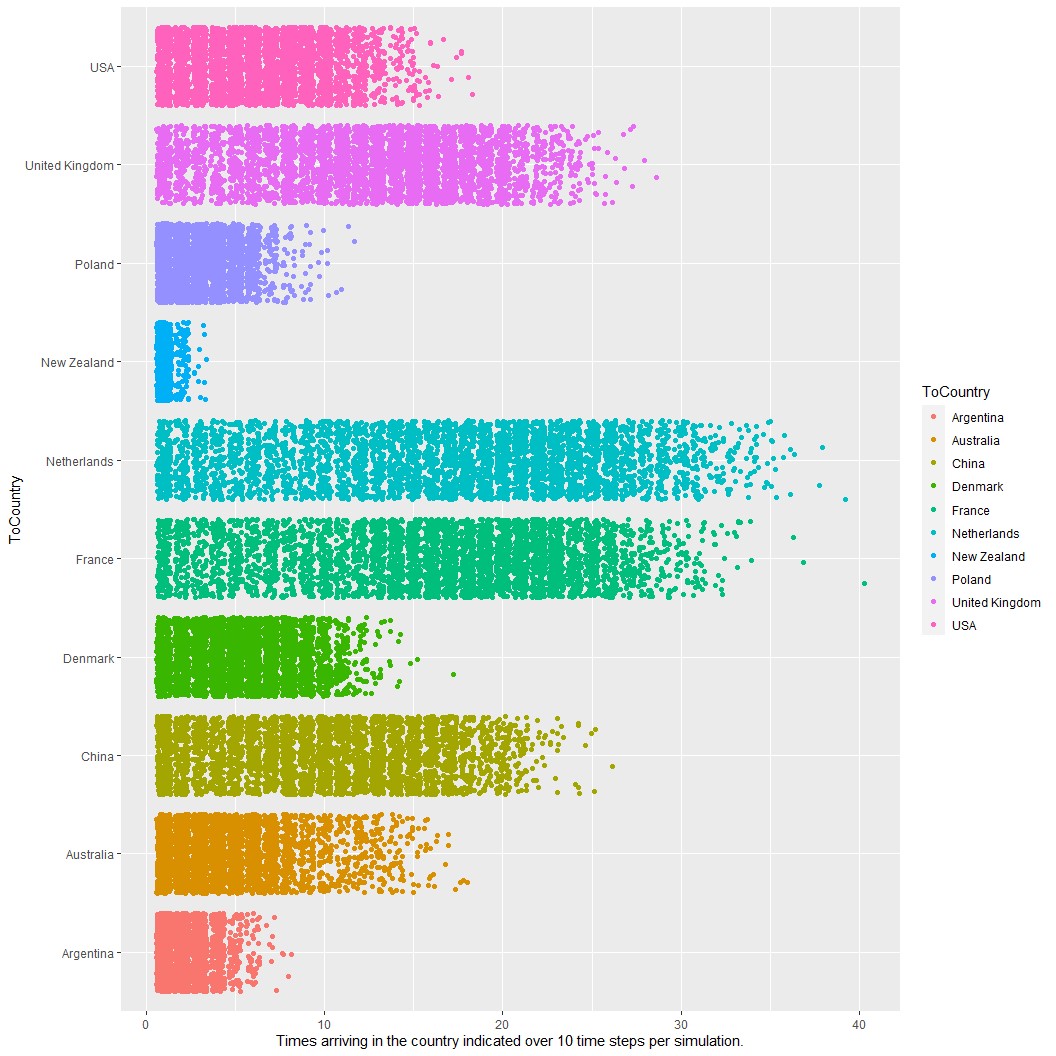


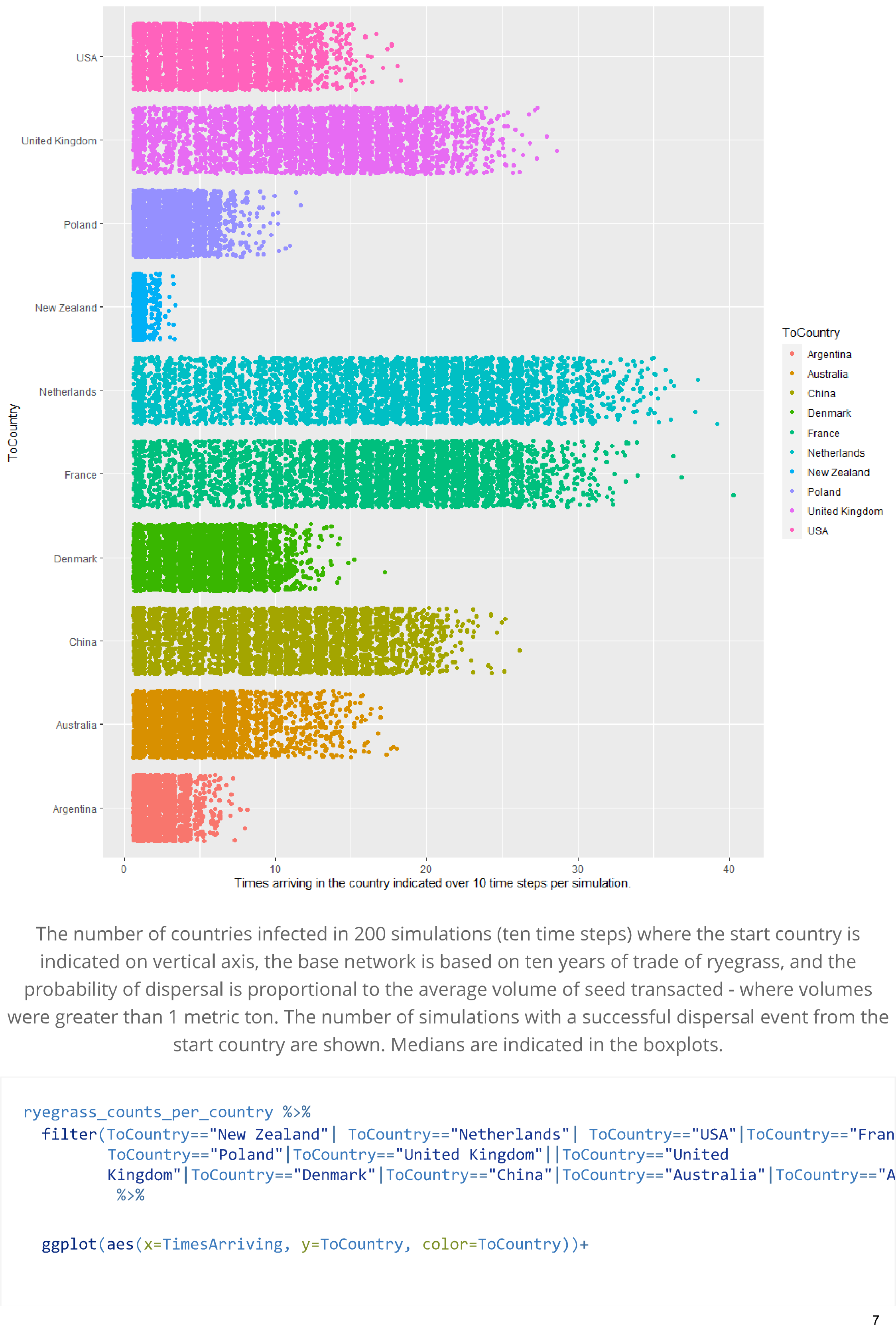


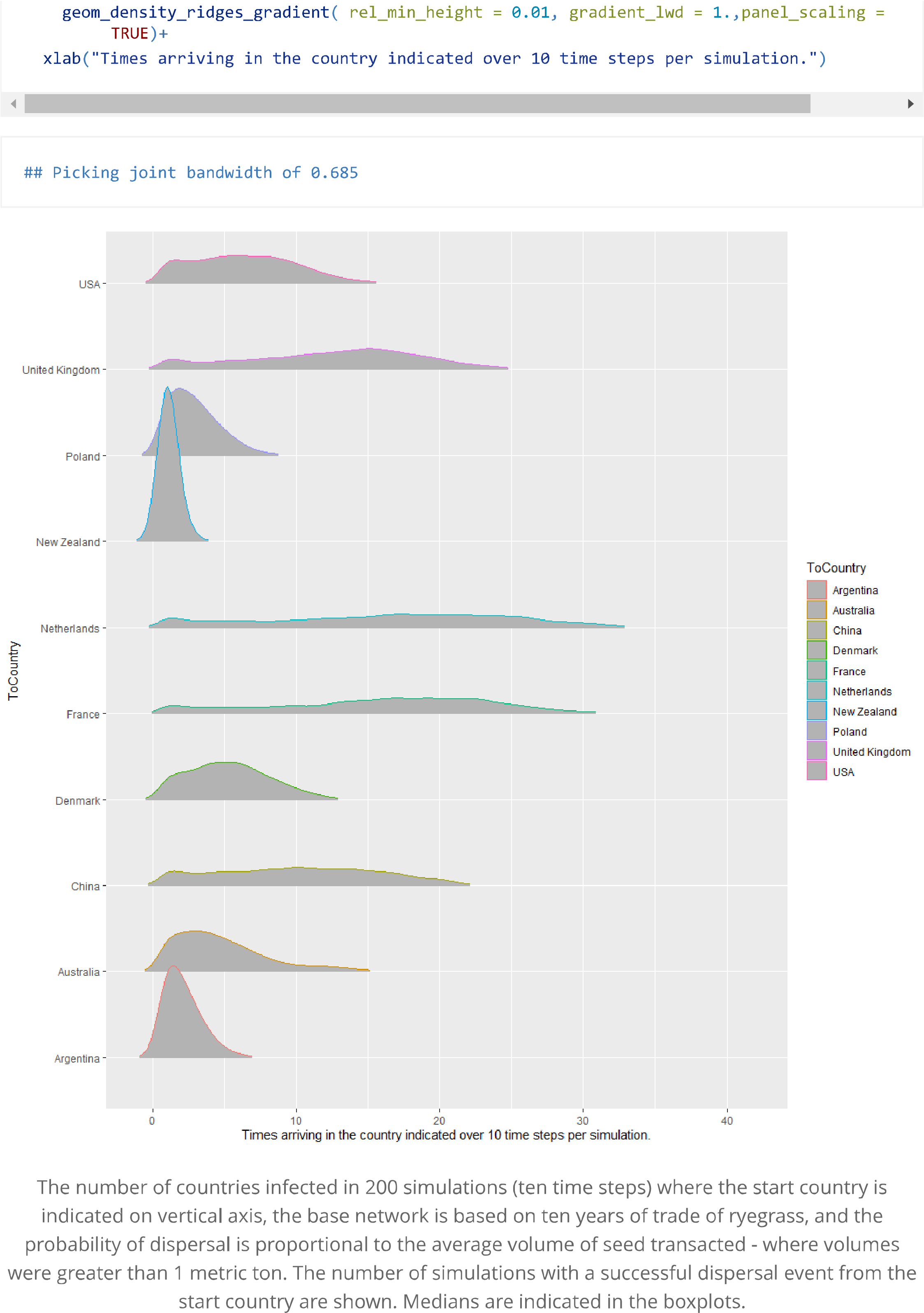


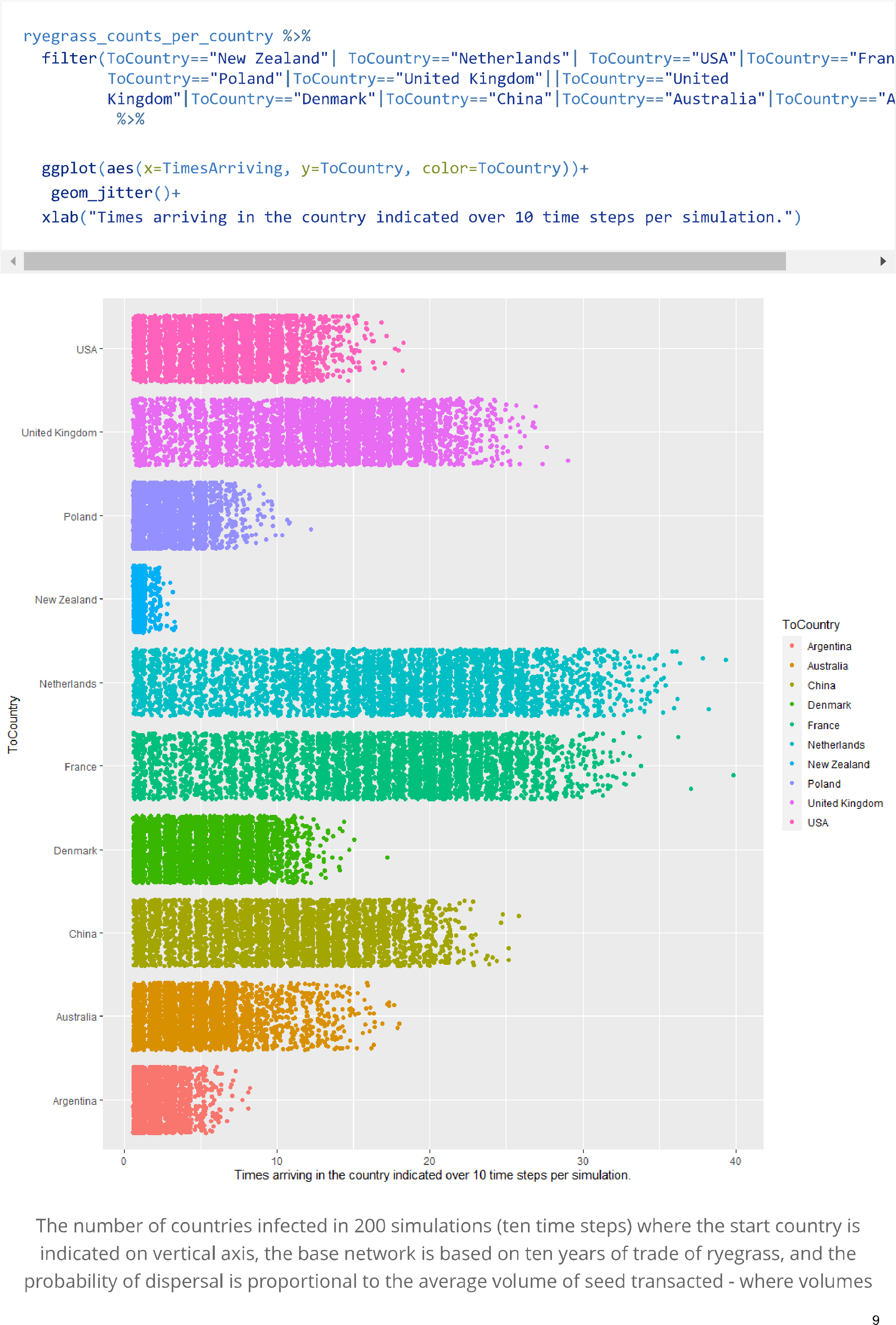


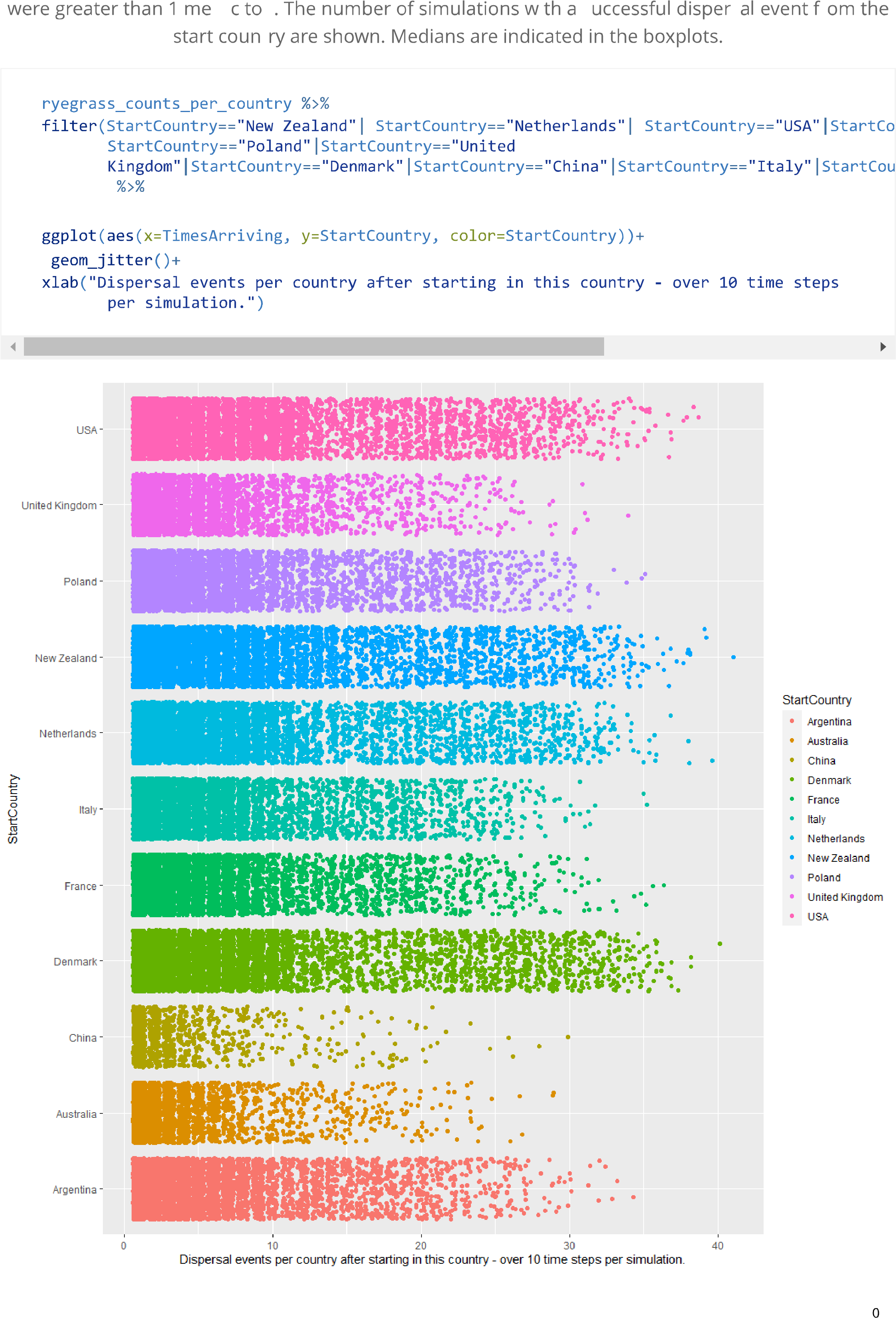


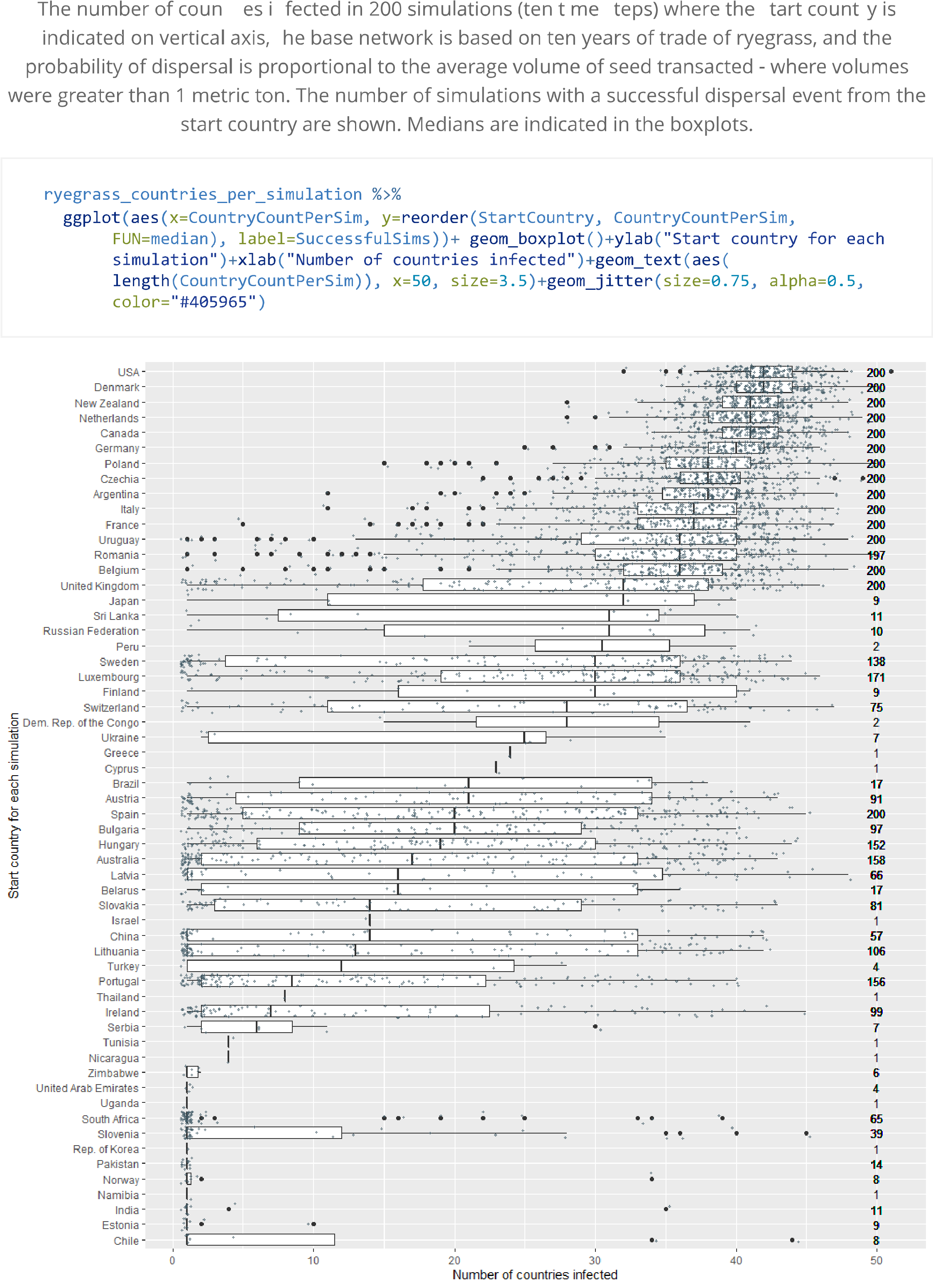


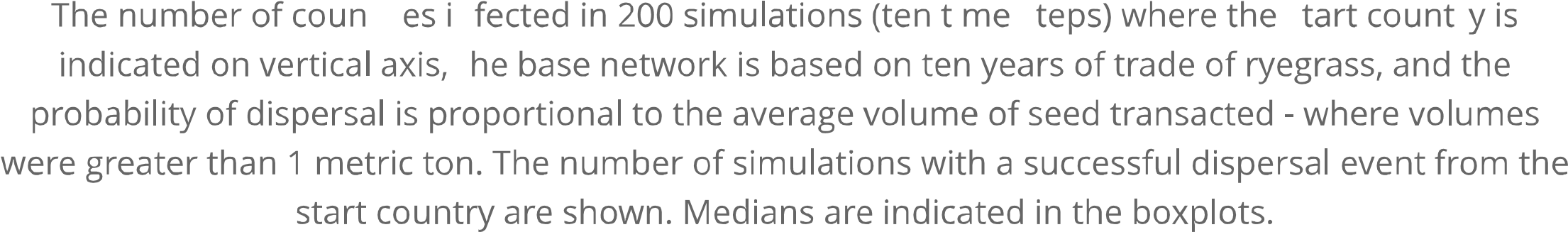


### Clover hypothetical contaminant spread simulations
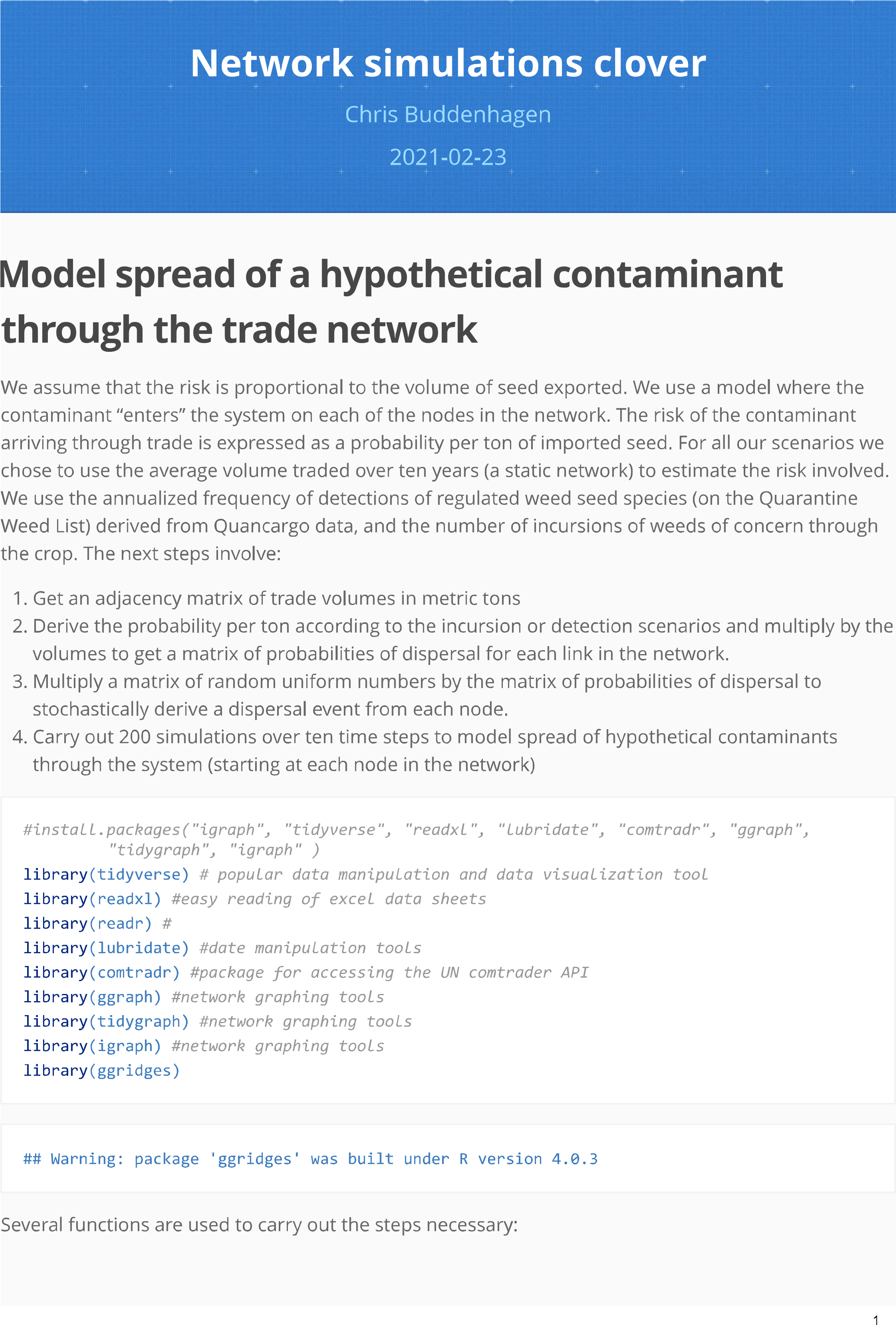


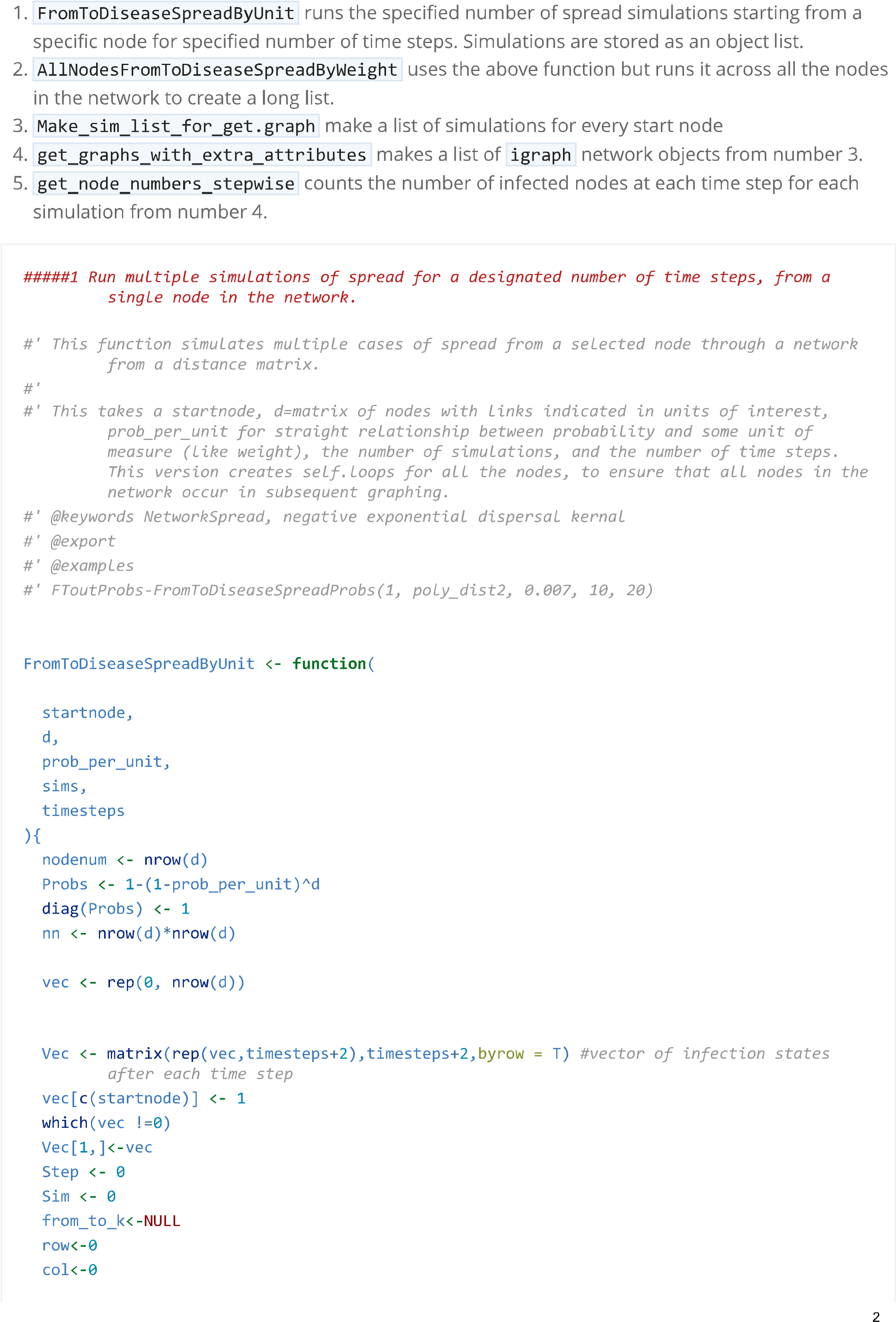


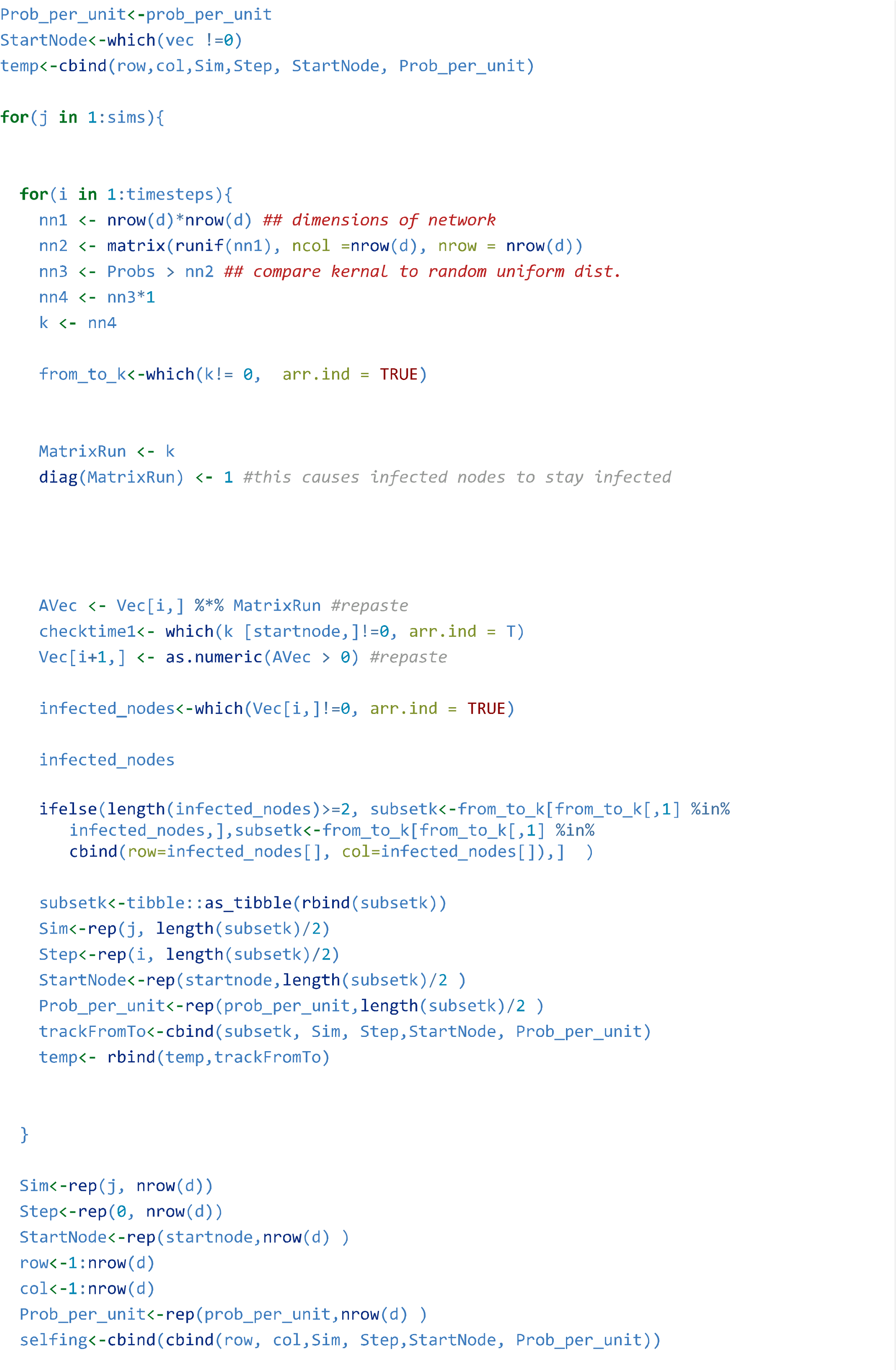


|  |
| --- |
|  |

|  | | |
| --- | --- | --- |
|  |  |  |

|  | | |
| --- | --- | --- |
|  |  |  |

### Heat map comparing simulation outputs to expected risk from trade volumes

### 6) Other outputs from the simulations
